# A systematic review of midwives’ training needs in perinatal mental health and related interventions

**DOI:** 10.3389/fpsyt.2024.1345738

**Published:** 2024-04-22

**Authors:** Marine Dubreucq, Corinne Dupont, Mijke P. Lambregtse-Van den Berg, Wichor M. Bramer, Catherine Massoubre, Julien Dubreucq

**Affiliations:** ^1^Centre referent de rehabilitation psychosociale, GCSMS REHACOOR 42, Saint-Étienne, France; ^2^University Claude Bernard Lyon1, Research on Healthcare Performance (RESHAPE) INSERM U1290, Lyon, France; ^3^AURORE Perinatal Network, Hospices civiles de Lyon, Croix Rousse Hospital, Lyon, France; ^4^Departments of Psychiatry and Child & Adolescent Psychiatry, Erasmus MC, University Medical Center Rotterdam, Rotterdam, Netherlands; ^5^Medical Library, Erasmus MC, University Medical Center Rotterdam, Rotterdam, Netherlands; ^6^University Hospital of Saint-Étienne & EA 7423 (Troubles du Comportement Alimentaire, Addictions et Poids Extrêmes (TAPE), Université Jean Monnet - Saint-Etienne), Saint-Etienne, France; ^7^University Hospital of Saint-Étienne, Department of Child and Adolescent Psychiatry, France & Marc Jeannerod Institute of Cognitive Sciences UMR 5229, CNRS & Claude Bernard University, Lyon, France

**Keywords:** midwifery, perinatal care, mental health services, education, attitude of health personnel, literature review

## Abstract

**Background:**

Midwives may be key stakeholders to improve perinatal mental healthcare (PMHC). Three systematic reviews considered midwives’ educational needs in perinatal mental health (PMH) or related interventions with a focus on depression or anxiety. This systematic review aims to review: 1) midwives’ educational/training needs in PMH; 2) the training programs in PMH and their effectiveness in improving PMHC.

**Methods:**

We searched six electronic databases using a search strategy designed by a biomedical information specialist. Inclusion criteria were: (1) focus on midwives; (2) reporting on training needs in PMH, perinatal mental health problems or related conditions or training programs; (3) using quantitative, qualitative or mixed-methods design. We used the Mixed Methods Appraisal Tool for study quality.

**Results:**

Of 4969 articles screened, 66 papers met eligibility criteria (47 on knowledge, skills or attitudes and 19 on training programs). Study quality was low to moderate in most studies. We found that midwives’ understanding of their role in PMHC (e.g. finding meaning in opening discussions about PMH; perception that screening, referral and support is part of their routine clinical duties) is determinant. Training programs had positive effects on proximal outcomes (e.g. knowledge) and contrasted effects on distal outcomes (e.g. number of referrals).

**Conclusions:**

This review generated novel insights to inform initial and continuous education curriculums on PMH (e.g. focus on midwives’ understanding on their role in PMHC or content on person-centered care).

**Registration details:**

The protocol is registered on PROSPERO (CRD42021285926)

## 1 Introduction

Perinatal Mental Health Problems (PMHPs) affect parents during pregnancy and the first year after childbirth and commonly consist of anxiety, non-psychotic depressive episode, psychotic episodes, post-traumatic stress disorder and adjustment disorder. Despite being often associated with poor parental and child outcomes (1), PMHPs remain predominantly unrecognized, undiagnosed and untreated (2).

Given their role in perinatal care providing multiple occasions to discuss perinatal mental health (3) - midwives may be key stakeholders to improve the detection, referral and management of PMHPs. Parents usually welcome midwives’ interest in their mental health and report to prefer discussing mental health issues with obstetric providers than with mental health providers (4, 5). Assessing perinatal mental health (PMH) and detecting symptoms of postpartum depression, anxiety and psychosis are part of the essential competencies for midwifery practice according to the International Confederation of Midwives (2019) (6). However, and despite being in general interested in assessing perinatal mental health (PMH) and wellbeing (7), midwives report feeling less comfortable with putting competencies related to PMH into practice compared to those related physical health (8, 9).

To our knowledge, three literature reviews have been conducted on midwives’ educational needs in perinatal mental health (7, 10, 11). These reviews reported a lack of knowledge, skills and confidence influential at different levels of the care pathway, e.g. detection, decision-making about referral and support. However, there remain some limitations to the current body of evidence. First, all reviews found low-to-moderate quality studies coming predominantly from high-income countries. Second, two out of three reviews (10, 11) - conducted in 2017 (n=17 articles) and 2022 (43 articles) - focused on perinatal depression or perinatal anxiety and did not cover the full range of PMHPs as well as related conditions (e.g. substance use disorder, serious mental illness (SMI)) or autism). The third review (7) conducted in 2017 (n=22 articles) covered a wider range of PMHPs using an integrative review design, the other two (10, 11) being systematic reviews. Third, previous reviews (7, 10, 11) focused on midwives’ knowledge, skills and attitudes and context-related factors. However, it remains unclear whether improvements in these areas translate into in routine clinical practice (e.g. improved detection of PMHPs or facilitated decision-making about referral to mental health providers). Fourth, case identification - using formal or informal screening methods - have contrasted effects on referral rates (7) and patient outcomes [e.g. limited effects of screening on depressive symptoms (12, 13)]. Fifth, two systematic reviews reported on training programs in perinatal depression [n=7 studies (10), n=12 studies (14)]. However, these reviews included mixed samples [e.g. 37% midwives in Wang et al., 2022 (14) and 54% midwives in Legere et al., 2017 (10)] and did not target the same set of skills [e.g. improving knowledge and detection (10); providing evidence-based interventions (14)]. Reviews either investigated midwives’ training needs (7, 11) or training interventions (10, 14). The literature on training programs in PMH for student midwives and midwives remains scarce [n=4 studies (10)]. A synthesis of evidence before this study is presented on **Table 1**.

**Table 1:** Evidence before this study.

Source and country	Type of review	Inclusion criteria	Articles included (N, population and conditions considered)	Proportion of midwives	Main results	Quality rating of included articles	Limitations identified by the authors
Branquinho et al., 2022 (11)	Systematic review (6 electronic databases: e Ovid Medline, PsycInfo, Embase, Cochrane Database of Systematic Reviews, Scopus and SveMed+)	-articles related to health providers’ perinatal depression literacy -quantitative, qualitative or mixed method studies -no limit on publication year -studies published in English	-N=43 studies included published between 1994 and 2020 (n=6755 participants) -Perinatal depression (from pregnancy to the first year after childbirth) n=25 on postpartum depression; n=16 on both antenatal and postnatal depression, n=2 on antenatal depression -health providers: GP, obstetrics/gynecologists, pediatricians, nurses, midwives, family practitioners, psychologists, social workers, psychiatrists Exclusion of pharmacists, medical or nursing students, volunteer workers, religious leaders, community leader and birth attendants - n=4 studies included a training program	N= 17 studies (9 with midwives only; 8 with midwives and other health providers)	Health providers had a moderate level of knowledge about PND. Most of them had correct recognition of PND but lack of knowledge about prevalence, risk factors, symptoms, screening tools and treatment options for PND Nurses had higher mean knowledge score about PND than midwives and obstetricians/gynecologists had a higher self-reported knowledge of PND Most health providers had a negative attitude toward PND and toward their role in screening and treatment of PND All the 4 studies including a training program shown a significant improvement of providers’ levels of general knowledge	Strong quality: n=31 studies Moderate quality: n=12 studies Low response rate and high rate of loss to follow-up, lack of validated instruments, no power calculation, no blind participants Tools used: - for qualitative studies: Critical Appraisal Skills Program - for quantitative cross-sectional studies: Quality Assessment Tool for Observational Cohort and Cross-Sectional - for quantitative pretest-posttest studies Quality Assessment Tool for Before-After (Pre-Post) Studies With No Control Group -for mixed methods studies: Mixed Methods Appraisal Tool	- exclusion of articles published in other languages than English -exclusion of the grey literature -use of various tools to assess risk of bias -most studies were cross sectional
Legere et al., 2017 (10)	Systematic review (7 electronic databases: CINAHL, Medline, Medline In Process, Cochrane Library (Cochrane database of Systematic Reviews and Cochrane Central Registry of Controlled Trials), Embase, ERIC, and PsycInfo)	- articles related to perinatal depression education and professional development for health providers - articles on midwives’ or nurses’ practices (screening, etc.) and related to perinatal mental health or articles related to assessment of existing experience or knowledge about perinatal mental health - quantitative, qualitative or mixed method studies, systematic reviews, meta-analyses or meta-syntheses studies -studies published between January 2006 and2015 -studies published in English	- N= 12 studies included published between 2006 and 2014 (n=2818 participants; 2 studies had unspecified sample) - Perinatal depression (PND; from pregnancy to the first year after childbirth) - 5 studies related to training needs and 7 studies relates to educational or professional development strategies to advance knowledge and skills in PND	N=8 studies (5 with midwives only; 3 with midwives and other health providers)	Providers reported a lack of training about PND. The existing programs were focused on PND screening with a moderate efficacy on parents outcomes (depressive symptoms)		-limitations associated to the studies included (low quality, small sample sizes, heterogeneity of providers -self reported scales -exclusion of articles publish before 2006 - exclusion of articles published in other languages than English -exclusion of the grey literature
Noonan et al., 2017 (7)	Integrative review (7 electronic databases: Cochrane Database of Systematic reviews, Medline, CINAHL, PsycInfo, Embase, SCOPUS, and Web of Science)	-studies related to midwives’ perceptions and experiences in care of parents with perinatal mental health problems -studies including mostly midwives working at hospital or in community centers -quantitative, qualitative or mixed method studies -studies published between January 2006 and 2016 -Studies from Europe, North America, Australia and New Zealand -studies published in English	- N=22 articles included(no detail of number of participants) - Perinatal mental health problems (anxiety, mood disorders or psychotic disorders) from pregnancy to the first year after childbirth - n=15 quantitative studies, n=6 qualitative studies and n=1 mixed method study	100% (15 with midwives only; 6 with midwives and other health providers; 1 with midwives and birthing persons)	Midwives reported personal (knowledge, skills, and attitude) and professional factors (continuous professional development, organization of care, referral, and support) influencing their engagement in perinatal mental health care Personal engagement covered knowledge, skills, decision making and attitude in perinatal mental health Professional engagement covered continuous professional development, organization of care, referral, and support	Good quality for most of the included studies Limitations of qualitative studies: data saturation, acknowledgment of the researcher/participant relationship, explicit statement of ethical approval and informed consent. Limitations of quantitative studies: Convenience sample and low response rate, no validated measure unclear validity and reliability of measures Tool used: Critical Appraisal Skills Program	-exclusion of articles publish before 2006 - limitations associated with computerized databases - methodology limitations - exclusion of articles published in other languages than English -exclusion of studies from low and moderate income countries
Wang et al., 2022 (14)	Systematic review and meta analysis (6 databases: PubMed, MEDLINE, Cochrane Library, EMBASE, Web of Science, and CINAHL)	-studies including midwives, nurses or health visitors or parents without preexisting mental health problems or with moderate- to high-risk PND in the absence of psychiatric symptoms -Studies related to psychological training programs -Studies using the Edinburgh Postnatal Depression Scale (EPDS) to report depressive symptoms (in this review, the cutoff scores were ≥12 and ≥10 - randomized controlled trials (RCTs) or quasi-experimental studies with at least one control group (no training or standard treatment training for PND). Exclusion of studies with a one-group pretest– posttest design, case reports, qualitative studies, conference abstracts, and studies including the administration of psychotherapy).	- N=13 articles included published between and (n=246 providers and 4381 perinatal persons) -Perinatal depression (from pregnancy to the first year after childbirth) -5 cluster RCT, 4 RCT and 4 non RCT	N=4 studies (3 with midwives only; 1 with midwives and other health providers)	Face-to-face and digital training decreased significantly PND symptoms Significant improvement of PND symptoms were observed after 3- to 5-day and 8-day training (no significant effect of 2-day or less training) Compared with studies with low intervention fidelity, those with high intervention fidelity reduced more effectively the risk of depressive symptoms	Good quality for most studies Tool used: fidelity review checklist	-Lack of appropriate blinding -self-reported questionnaires -attrition bias -lack of precisions in the duration of training programs

GP, General Practitioners; PND, Perinatal Depression; RCTs, randomized controlled trials.

The present review primarily aims to identify and review: 1) midwives’ educational/training needs in PMH (i.e. beyond perinatal depression or anxiety to include PMHPs, SMI, substance use disorder, and autism); 2) the existing interventions and their effectiveness in improving detection and management of PMHPs.

## 2 Methods

### 2.1 Search strategy

The protocol for this systematic review was reported according to PRISMA guidelines (15). The search strategy was designed by a biomedical information specialist (WMB) from the Medical Library of Erasmus MC, University Medical Center Rotterdam (16). We searched Embase, MEDLINE, Web of Science, Cochrane Central Register of Controlled Trials, CINAHL and, PsycINFO for published, peer reviewed original articles. The search combined terms for (1) perinatal mental health problems, serious mental illness (i.e. schizophrenia, mood disorders, personality disorders, anxiety), eating disorders, substance use disorders or autism, and (2) midwives’ knowledge, attitudes, skills or training needs, as well as existing training programs for midwives on PMH. We included only published articles in English or French. No time restriction was set. The search was updated prior to publication on 21 June 2023. We hand-searched the reference list of three systematic literature reviews (7, 10, 11) for additional relevant articles. The full search strategy, search terms and syntax are presented in online **Supplementary Table 1**.

### 2.2 Inclusion/exclusion criteria

To be included, articles had to meet all the following criteria: 1) focus on midwives (included midwives, nurse-midwives, registered midwives, registered midwives tutors, registered midwives prescribers and registered advanced midwives practitioners - referred as “midwives” in this review); 2) reporting on midwives’ training needs in PMH, PMHPs or related conditions or existing training programs that focus on the use of screening tools to detect PMHPs, on PMH in general or specific aspects of PMH; 3) using quantitative, qualitative or mixed-methods design. For training programs, we included uncontrolled and controlled studies (placebo, TAU or active comparators).

Our exclusion criteria were: 1) no full text available or studies published in languages other than English or French; 2) grey literature because the aim of this systematic review was to guide the development of future interventions; 3) training programs on psychological interventions (e.g. cognitive behavior therapy) because this review focused on interventions aiming at improving midwives’ training on essential competencies related to PMH (e.g. PMH assessment, detection, referral and support of parents with PMHPs).

### 2.3 Selection and coding

The screening process was conducted in two separate stages: 1) Two authors (M.D. and J.D) independently screened the title and abstracts of all non-duplicated papers excluding those not relevant. Potential discrepancies were resolved by consensus; 2) Two authors (M.D. and J.D) independently applied eligibility criteria and screened the full-text papers to select the included studies. Disputed items were solved discussing together and reading further the paper to reach a final decision. **Supplementary Tables 2**, **3** present the list of included/excluded studies. Inter-rater reliability was calculated (kappa=0.90).

### 2.4 Data extraction

Two authors (MD and JD) performed independently the data extraction. For each study, we extracted the following information: general information (author, year of publication, country, design, type of study, population considered, period), assessment tools or methods, cultural aspects, the main findings and variables relating to quality assessment. For studies reporting on training programs, we also extracted information about the intervention (nature, type, length, targeted skills or outcomes, format), outcome measures and effectiveness on midwives’ knowledge, attitude, skills or routine use of screening tools to detect PMHPs or parents’ outcomes (e.g. depressive symptoms). **Tables 2**–**6** present the factors associated with knowledge, skills, confidence and decisions about screening, referral or support. **Supplementary Tables 4**, **5** present the detailed characteristics of the included studies.

**Table 2:** Factors influencing the level of knowledge and skills.

	Influencing factors	Knowledge/skills	
		Significance (for quantitative studies only)	Direction of the relationship
**Provider level**	***Background***		
	Age	-Jones 2011 (17) (younger)	-Jones 2011 (17) (+) (younger)
	Sex	-Jones 2011 (17) (NS)	
	Education level	-Jones 2011 (17)	-Jones 2011 (17) (+)
	Years of experience	-Magdalena 2020 (9) (shorter experience/postpartum depression) -Buist 2006 (18) (NS) -Hauck 2015 (8) (NS) - Işık 2010 (19) (NS) (PPD) -Jones 2011 (17) (NS) -Magdalena 2020 (9) (NS) (antenatal depression)	-Salomonsson 2011 (+) (20) -Magdalena 2020 (9) (–) -Savory et al., 2022 (21) (–)
	Mental health nursing experience/experience in psychiatry	-Hauck 2015 (8) (NS)	
	Type of practice/work context (community vs. hospital)	-Salomonsson 2011 (20) -De Vries 2020 (22) (NS) (about FOC or PTSD) -Jones 2012, 2011 (17) (NS)	-Salomonsson 2011 (20) (+)
	Personal interest in PMH		-Andersen 2023 (23) (+) (in undertaking psychosocial assessment and asking sensitive questions)
	Previous training in PMH	-Carroll 2018 (24) -Higgins 2017 (25), 2018 (26) -Işık 2010 (19) -Hauck 2015 (8) (NS)	-Carroll 2018 (24) (+) -Higgins 2017 (25); 2018 (26) (+) -Işık 2010 (19) (+) -Salomonsson 2011 (20) (+) -Savory et al., 2022 (21) (–) -Keng 2005 (27) (-)
	Frequent work with parents with mental health problems	-Isik 2010 (19) -Hauck 2015 (8) (NS)	-Isik 2010 (19) (+)
	***Knowledge***		
	Signs and symptoms	-Buist 2006 (18) (NS)	-Higgins 2018 (26) (-)
	Referral pathways/available resources		-Higgins 2018 (26) (-)
	Assessment skills		-Higgins 2018 (26) (-)
	***Attitudes***		
	Perceived role in PMHC		-Higgins 2018 (26) (+)
	***Correct case identification***	-Magdalena et al., 2020 (9)	-Magdalena et al., 2020 (9)
	***Experience in conducting psychosocial assessment***		- Andersen 2023 (23) (+) (on verbal and non verbal relational skills)
	***Self-efficacy in providing PMHC***	-Noonanet al. 2018 (28) -Noonan et al., 2019 (29) -Hauck 2015 (8) (NS)	-Noonan et al., 2018 (28) -Noonan et al., 2019 (29)
**Organizational level**	***Local policy***		
	Routine use of screening tools		-Williams 2016 (5) (not change their ability to detect antenatal depression)
	***Continuity of care***		
	Available resources		-Higgins 2018 (26) (-)
	Clear referral pathway		-Higgins 2018 (26) (-)
	***Barriers to access care***		
	Workload/lack of time		-Higgins 2018 (26) (-)
	***Enablers to access care***		
	Specialist team		-Stewart 2002 (30) (-)
**Parents level**	Ethnicity		-Edge 2010 (31)
	***Lack of privacy***		-Higgins 2018 (26) (-)

FOC, Fear of Childbirth; NS, non significant; PMH, Perinatal Mental Health; PMHC, Perinatal Mental Health Care; SUD, Substance Use Disorder.

**(+)**, positive relationship; **(-)**, negative relationship.

**Table 3:** Factors influencing confidence and the perception of being well-equipped.

	Influencing factors	Confidence/Perception of being well-equipped	
		Significance (for quantitative studies only)	Direction of the relationship
**Provider level**	***Background***		
	Age	-Noonan 2019 (29) (NS)	-Whitehead 2019 (32) (+) (SUD)
	Education level	-Noonan 2019 (29) (NS)	
	Years of experience	-Cunningham 2019 (33) -Salomonsson 2011 (20)	-Cunningham 2019 (33) (+) -Salomonsson 2011 (20) (+) -Whitehead 2019 (32) (+) (SUD) -Savory 2022 (21) (–)
	Mental health nursing experience/experience in psychiatry	-Hauck 2015 (8)	-Hauck 2015 (8) (+) -Williams 2016 (5) (+) (in asking question)
	Type of practice/work context	-Salomonsson 2011 (20) (+)	-Salomonsson 2011 (20) (+)
	Personal interest in PMH		-Andersen 2023 (23) (+)
	Personal/family experience of mental health problems	-Noonan 2019 (29) (NS)	
	Previous training in PMH	-Carroll 2018 (24) -Magdalena 2020 (9) (training about screening and management) -Higgins 2017 (25) -Cunningham 2019 (33) (NS) -Noonan 2019 (29) (NS)	-Carroll 2018 (24) (+) - Dubreucq 2019 (34) (+) -Higgins 2017 (25) (+) -Magdalena 2020 (9) (training about screening and management) -Phillips 2015 (35) (+) -Whitehead 2019 (32) (+) (SUD) -Jones 2011 (17) (–) -Savory 2022 (21) (–)
	Frequent work with parents with mental health problems	-Noonan 2019 (29) (NS) (on confidence to manage PMHD)	
	***Knowledge***		
	Signs and symptoms	- Hauck 2015 (8) (correct identification) - Noonan 2018 (28) - Noonan 2019 (29)	-Bye et al., 2018 (36) (+) -Dubreucq 2019 (34) (+) -Hauck 2015 (8) (+) -Edge 2010 (31) -Işık & Bilgili, 2010 (19) -Jones 2011 (17) -Magdalena & Tamara 2020 (9) -Whitehead 2019 (32) (+) (SUD) -Noonan 2018 (28) (+) -Noonan 2019 (29) (+)
	Screening tools		-Jones 2011 (17) -Phillips 2015 (35) (+)
	Referral pathways/available resources		-Phillips 2015 (35) (+)
	Treatment options		-Jones 2011 (17) -Jones 2012b (37)
	Interviewing skills		-Dubreucq 2019 (34) (+) -Oni 2020 (38) (+) (SUD)
	Consequences		-Bye et al., 2018 (36)
	***Skills***		
	Assessment skills		-Noonan 2018 (28)
	***Attitudes***		
	Perceived role in PMHC		-Whitehead 2019 (32) (+) (SUD) -McCauley (39) (–)
	Stigma		
	- Perceived dangerousness		-Phillips 2015 (35) (+)
	- Perceived inability to provide adequate childcare		-Phillips 2015 (35) (+)
	***Correct case identification***	-Hauck et al., 2015 (8) (bipolar disorder)	-Hauck et al., 2015 (8) (+) (bipolar disorder)
	***Experience in conducting psychosocial assessment***		- Andersen 2023 (23) (+) (on confidence in asking sensitive questions)
**Organizational level**	***Continuity of care***		
	Clear referral pathway		-Oni 2020 (38) (+) (SUD)
	***Enablers to access care***		
	Access to reactive specialist care		- Dubreucq 2019 (34) (+)
	Specialist team	-Salomonsson 2011 (20) (+)	-Salomonsson 2011 (20) (+)
	Support from team members		-Andersen 2023 (23) (+) -Whitehead 2019 (32) (+) (SUD)
	Supervision		-Andersen 2023 (23) (+)
**Parents level**	Ethnicity		-Edge 2010 (31)
**Interaction level**	***Person-centered care***		- Dubreucq 2019 (34) (+)

FOC, Fear of Childbirth; NS, non significant; PMH, Perinatal Mental Health; PMHC, Perinatal Mental Health Care; SUD, Substance Use Disorder.

**(+)**, positive relationship; (–), negative relationship.

**Table 4:** Factors influencing decisions about screening.

	Influencing factors	Screening	
		Significance (for quantitative studies only)	Direction of the relationship
**Provider level**	***Background***		
	Age	-Lau 2015 (40) (NS)	
	Education level	-Sanders 2006 (41) (+) (DNP) -Lau 2015 (40) (NS)	-Sanders 2006 (41) (+) (DNP)
	Years of experience	-Fontein-Kuipers 2014 (42) (shorter)	-Fontein-Kuipers 2014 (42) (+) (shorter)
	Mental health nursing experience	-Lau 2015 (40)	-Lau 2015 (40) (+) (suicide risk assessment)
	Type of practice/work context		-Keng 2005 (27) (+) (maternity vs. labor ward) -Salomonsson 2011 (20) (+)
	Personal interest in PMH	-Fontein-Kuipers 2014 (42) (+)	-Fontein-Kuipers 2014 (42) (+) -Jarrett 2015 (43) (+)
	Personal attitudes toward PMHD		-Shahid Ali 2023 (44) (+)
	Personal/family experience of mental health problems		-Noonan 2019 (29) (+) -Fletcher 2021 (45) (–)
	Previous training in PMH		-Andersen 2023 (23) (+) -Bye 2018 (36) (+) -Oni 2020 (38) (+) (SUD) -Savory 2022 (21) (+) -Whitehead 2019 (32) (+) (SUD)
	Frequent work with parents with mental health problems		-Carroll 2018 (24) -Gibbs 2007 (46) (+) -Higgins 2017 (25) (+) -Jarrett 2015 (43) (+) -McCauley 2011 (39) (+) -Oni 2020 (38) (+) (SUD) -Sanders 2006 (41) (+) -Savory 2022 (21) (+) -Stewart 2002 (30) (+)
	***Knowledge***		
	Prevalence		-Jones 2011 (17) (+)
	Signs and symptoms	-Magdalena 2020 (9) (case identification) -Sanders 2006 (41) (DNP)	-Asare 2022 (47) (+) -Bye 2018 (36) (+) (ED) -Edge 2010 (31) (+) -Higgins 2018 (26) (+) -Jomeen 2009 (48) (–) (for antenatal depression) -Jones 2011 (17) (+) -Magdalena 2020 (9) (+) -McGlone 2016 (49) (+) -McGookin 2017 (50) (+) -Ross-Davie 2006 (51) (+) -Shahid Ali 2023 (44) (+) -Sanders 2006 (41) (+) (DPN) -Schouten 2021 (52) (+) (DPN) -Whitehead 2019 (32) (+) (SUD)
	Risk factors		-Jones 2011 (17) (+) -Noonan 2018 (28)
	Consequences		-Jones 2011 (17) (+) -Oni 2020 (38) (+) -Whitehead 2019 (32) (+) (SUD)
	Screening tools		-Andersen 2023 (23) (+) -Higgins 2017 (25) (+) -Jones 2011 (17) (+) -Madden 2018 (53) (+) (need to discuss the meaning of PMH, guidance about the use of the Whooley questions) -Magdalena 2020 (9) -McGlone 2016 (49) (+) -McGookin 2017 (50) -Oni 2020 (38) -Philips 2015 (35) -Savory 2022 (21) -Hauck 2015 (8) (–)
	Treatment options		-Bye 2018 (36) (+) -Higgins 2017 (25) -Higgins 2018 (26) -Jones 2011 (17) (+) -Jones 2012b (37) -McGookin 2017 (50) (+) - Savory 2022 (21) - Stewart 2022 (30) -Williams 2016 (5) (+) -Whitehead 2019 (32) (+) (SUD)
	Local/national/guidance/guidelines		-McGookin 2017 (50) (+) -Noonan 2018 (28) (+) -Noonan 2019 (29) (+) -Savory 2022 (21) (+) -Stewart 2002 (30) (+) -Sanders 2006 (41) (–)
	Available resources		-Bye 2018 (36) (+) -McGookin 2017 (50) (+) -Oni 2020 (38) (+) (SUD)
	Referral pathways		-Andersen 2023 (23) (+) -Bye 2018 (36) (+) -McGlone 2016 (49) (+) -McGookin 2017 (50) (+) -Stewart 2002 (30) -Williams 2016 (5) (+) -Whitehead 2019 (32) (+) (SUD)
	Cultural aspects		-Noonan 2019 (29) (+) -Schouten 2021 (52) (+)
	***Skills***		-Edge 2010 (31) (+) -Ross-Davie 2006 (51) (+) -Shahid Ali 2023 (44) (+)
	Interviewing skills		-Bye 2018 (36) (+) (ED) -Carroll 2018 (24) -Cunningham 2019 (33) (+) -Dubreucq 2019 (34) -Fletcher 2021 (45) (+) -Gibbs 2007 (46) (+) -Higgins 2017 (25) -Higgins 2018 (26) (+) (fear of negative reaction) -Jarrett 2014 (54) (+) -Jones 2012a (55) (+) -Madden 2018 (53) (+) -McCauley 2011 (39) -McGlone 2016 (49) (+) -McGookin 2017 (50) (+) -Noonan 2018 (28) (+) -Oni 2020 (38) (+) (SUD) -Phillips 2015 (35) -Savory 2022 (21) (+) -Sanders 2006 (41) (+) (DNP) -Willey 2020 (56) (+) -Whitehead 2019 (32) (+) (SUD)
	Communication skills		-McGlone 2016 (49) (+) -McGookin 2017 (50) (+)
	Distress management		-Asare 2022 (47) (+) -Carroll 2018 (24) (+) -Higgins 2018 (26) (+) -McGlone 2016 (49) (+) -Ross-Davie 2006 (51) (+) -Noonan 2018 (28) (+) -Savory 2022 (21) (+)
	Listening/Non-judgmental and supportive approach		-Asare 2022 (47) (+) -Stewart 2002 (30)
	***Attitudes***		
	Toward screening	-Fontein-Kuipers 2014 (42) -Sanders 2006 (41) (DPN)	-Asare 2022 (47) (+) -Edge 2010 (31) (+) -Fletcher 2021 (45) (+) -Fontein-Kuipers 2014 (42) (+) -McGookin (50) 2017 (+) -Oni 2020 (38) -Sanders 2006 (41) (DPN) (+) -Jarrett 2015 (43) (–)
	Toward screening tools		-Edge 2010 (31) (+) -Fletcher 2021 (45) (+) -Gibbs 2007 (46) (+) -Jarrett 2014 (54) (+) -Jones 2012b (37) (+) -McGlone 2016 (49) (+) (no clear understand of purpose) -Noonan 2019 (29) (+) -Phillips 2015 (35) (+) -Sanders 2006 (41) (+) (DPN) -Savory 2022 (21) (+) -Williams 2016 (5) (+) -Whitehead 2019 (32) (+) (SUD) -Jarrett 2015 (43) (–)
	Perceived role in PMHC		-Andersen 2023 (23) (+) -Fletcher 2021 (45) (+) -Fontein-Kuipers 2014 (42) (+) -Jarrett 2015 (43) (+) -Jones 2012a (55) (+) (depression and anxiety) -McGookin 2017 (50) (+) (role in managing perpetuating factors) -Ross-Davie 2006 (51) (+) -Rothera 2011 (57) (+) -Savory 2022 (21) (+) -Schouten 2021 (52) (+) (DPN) -Willey 2020 (56) (+) -Williams 2016 (5) (+)
	Stigma		
	- Perceived dangerousness		-Higgins 2018 (26) (–) -Jarrett 2014 (54) (–) -Jarrett 2015 (43) (–) -Ross-Davie 2006 (51) (–)
	- Perceived inability to provide adequate childcare		-Jarrett 2014 (54) (–)
	- Perceived inability to engage in antenatal care		-Shahid Ali 2023 (44) (–) -Whitehead 2019 (32) (–) (SUD)
	- Cultural bias		-Asare 2022 (47) (–) -Edge 2010 (31) (–) -Jarrett 2014 (54) (–) - Schouten 2021 (52) (–)
	Attitude toward referral		-Fletcher 2021 (45) (+)
	- Sharing sensitive information with other health providers/Trust in other HPs		-Bye 2018 (36) (–)
	Attitude toward support/management		-Jomeen 2009 (48) (–) (for antenatal depression)
	***Self-efficacy in providing PMHC***	-Fontein-Kuipers 2014 (42) -Sanders 2006 (32) (DPN)	-Andersen 2023 (23) (+) -Bye 2018 (36) (+) -Edge 2010 (31) (+) -Fontein-Kuipers 2014 (42) (+) -Jarrett 2015 (43) (+) -McGlone 2016 (49) (+) -McGookin 2017 (50) (+) -Ross-Davie 2006 (51) (+) -Sanders 2006 (41) (+) (DNP) -Whitehead 2019 (32) (+) (SUD)
	***Emotional impact on the midwife/discomfort***		-Bye 2018 (36) (–) -Oni 2020 (38) (–) (SUD)
**Organizational level**	***Local policy***		-Asare 2022 (47) (+)
	Routine use of screening tools		-Asare 2022 (47) (+) -Cunningham 2019 (33) (+) -Higgins 2017 (25) -Oni 2020 (38) (+) (SUD) -Whitehead 2019 (32) (+) (SUD)
	Compulsory screening/computerized notes		-Jarrett 2015 (43) (+) -Williams 2016 (5) (+)
	Lack of culturally sensitive screening tools		-Schouten 2021 (52) (+)
	***Continuity of care***		-Bye 2018 (36) (+) -Cunningham 2019 (33) (+) -Edge 2010 (31) (+) -Fletcher 2021 (45) (+) -Higgins 2018 (26) (+) -Oni 2020 (38) (+) (SUD) -Savory 2022 (21) (+) -Willey 2020 (56) (+) -Whitehead 2019 (32) (+) (SUD)
	Available resources		-Asare 2022 (47) (+) -Oni 2020 (38) (+) (SUD) -Whitehead 2019 (32) (+) (SUD)
	Clear referral pathway		-Asare 2022 (47) (+) -Edge 2010 (31) (+) -Fletcher 2021 (45) (+) -Higgins 2018 (26) (+) -Madden 2018 (53) (+) -Willey 2020 (56) (+)
	Home visits		Cunningham 2019 (33) (+)
	***Barriers to access care***		
	Lack of time/workload		-Andersen 2023 (23) (–) -Asare 2022 (47) (–) -Bye 2018 (36) (–) -Cunningham 2019 (33) (–) -Edge 2010 (31) (–) -Fletcher 2021 (45) (–) -Higgins 2018 (26) (–) -Jones 2012a (55) (–) -Madden 2018 (53) (–) -McGlone 2016 (49) (–) -McGookin 2017 (50) (–) -Noonan 2018 (28) (–) -Oni 2020 (38) (–) (SUD) -Ross-Davie 2006 (51) (–) -Savory 2022 (21) (–) -Schouten 2021 (52) (–) -Willey 2020 (56) (–) -Williams 2016 (5) (–) -Whitehead 2019 (32) (–) (SUD)
	Lack of communication between providers in their services		-Whitehead 2019 (32) (–) (SUD)
	Long waiting times for a visit (insufficient number of staff members)		-Asare 2022 (47) (–)
	Lack of confidentiality		-Asare 2022 (47) (–)
	***Enablers to access care***		
	Multidisciplinary work		-Bye 2018 (36) (+) -Oni 2020 (38) (+)
	Access to reactive specialist care		-Edge 2010 (31) (+)
	Specialist midwives		-McGookin 2017 (50) (+) -Jarret 2015 (43) (–)
	Specialist team		-Higgins 2018 (26) (+) -Oni 2020 (38) (+)
	Support from team members		-Andersen 2023 (23) (+) -Willey 2020 (56) (+) -Whitehead 2019 (32) (+) (SUD)
	Supervision		-Andersen 2023 (23) (+) -Fletcher 2021 (45) (+)
**Parents level**	***Stigma***		
	In the media		-Whitehead 2019 (32) (–) (SUD)
	Public stigma		-Schouten 2021 (52) (–) -Whitehead 2019 (32) (–) (SUD)
	Negative attitudes toward help-seeking		-Bye 2018 (36) (–) -Oni 2020 (38) (–) (SUD) -Schouten 2021 (52) (–) -Whitehead 2019 (32) (–) (SUD)
	Anticipated stigma		-Bye 2018 (36) (–) -Cunningham 2019 (33) (–) -Oni 2020 (38) (–) (SUD) -Schouten (52) (–) 2021 -Williams 2016 (5) (–) -Whitehead 2019 (32) (–) (SUD)
	Experienced stigma		-Bye 2018 (36) (–) -Shahid Ali 2023 (44) (–) -Williams 2016 (5) (–)
	***Negative experiences with HPs***		-Edge 2010 (31) (–) -Schouten 2021 (52) (–) -Williams 2016 (5) (–)
	***Lack of trust in health providers***		-Schouten 2021 (52) (–) -Williams 2016 (5) (–)
	***Cultural aspects (taboo, religious coping)***		-Schouten 2021 (52) (–)
	***Insufficient language proficiency/learning difficulties***		-Schouten 2021 (52) (–) -Willey 2020 (56) (–) -Williams 2016 (5) (–)
	***Presence of the partner***		-Higgins 2018 (26) (–) -Schouten 2021 (52) (–) -Williams 2016 (5) (–)
**Interaction level**	***Established relationship***		-Gibbs 2007 (46) (+) -Savory 2022 (21) (+) -Williams 2016 (5) (+) -Whitehead 2019 (32) (+) (SUD - on disclosure)
	***Building a trusting relationship***		-Willey 2020 (56) (+) -Williams 2016 (5) (+) -Whitehead 2019 (32) (+) (SUD - on disclosure)
	***Empathy***		-Whitehead 2019 (32) (+) (SUD - on disclosure)
	***Person-centered care***		-Bye 2018 (36) (+) -Cunningham 2019 (33) (+) -Oni 2020 (38) (+) (SUD)

FOC, Fear of Childbitrh; NS, non significant; PMH, Perinatal Mental Health; PMHC, Perinatal Mental Health Care; SUD, Substance Use Disorder.

**(+)**, positive relationship; (–), negative relationship.

**Table 5:** Factors influencing decisions about referral.

	Influencing factors	Referral	
		Significance	Direction of the relationship
**Provider level**	***Background***		
	Years of experience	-Fontein-Kuipers 2014 (42) (+)	-Fontein-Kuipers 2014 (42) (+)
	Type of practice/work context		-De Vries 2020 (22) (+)
	Personal interest in PMH	-Fontein-Kuipers 2014 (42) (+)	-Fontein-Kuipers 2014 (42) (+)
	Previous training in PMH		-Oni 2020 (38) (+) (SUD)
	Frequent work with parents with mental health problems		-Jomeen 2009 (48) (–) (for antenatal depression) -Oni 2020 (38) (+) (SUD)
	***Knowledge***		
	Signs and symptoms	-Magdalena 2020 (9) (positive attitude toward referral to mental health specialist)	-Asare 2022 (47) (+) (over-referral related to lack of knowledge) -Magdalena 2020 (9) (+) (positive attitude toward referral to mental health specialist) -McCauley 2011 (39) (+) -Rothera 2011 (57) (+)
	Consequences		-Oni 2020 (38) (+) (SUD)
	Available resources		-McCauley 2011 (39) (+) -Oni 2020 (38) (+) (SUD)
	Referral pathways		-Williams 2016 (5) -Whitehead 2019 (32) (+) (SUD)
	Role of other health providers		-McCauley 2011 (39) (+)
	Cultural aspects		-Noonan 2019 (29) (+)
	***Skills***		-Asare 2022 (47) (+) (over-referral related to lack of skills) -Rothera 2011 (57) (+)
	***Attitudes***		
	Perceived role in PMHC		-Rothera 2011 (57) (–) (role in management)
	Attitude toward referral		-McCann & Clark 2010 (58) (+) -McCauley 2011 (39) (fear of labeling) -Savory 2022 (21) (+)
	- Negative attitude toward sharing sensitive information with other health providers		-Bye 2018 (36) (–)
	- Trust in other HPs/intention to collaborate		-Fontein-Kuipers 2014 (42) -Jones 2012b (37) (+) -McCann & Clark 2010 (58) (+) -McCauley 2011 (39) (+) -Savory 2022 (21)
	- Parents’ preferences in decision-making about referral		-Madden 2018 (53) (+)
	Attitude toward support/management		-McCann & Clark 2010 (58) (+)
	- Pharmacological treatment		-McCann & Clark 2010 (58) (+)
	- Support groups		-McCann & Clark 2010 (58) (+)
	***Self-efficacy in providing PMHC***		-Jarrett 2015 (43) (+) -Jones 2012a (55) (+) -Madden 2018 (53) (+) -Jones 2012b (37) (–)
**Organizational level**	***Local policy***		
	Routine use of screening tools		-Oni 2020 (38) (+) (SUD)
	***Continuity of care***		-Edge 2010 (31) (+) -Oni 2020 (38) (+) (SUD)
	Available resources		-McCauley 2011 (39) (+) -Oni 2020 (38) (–) (SUD)
	Clear referral pathway		-Edge 2010 (31) (+) -Madden 2018 (53) (+) -McGlone (49) (+) -Noonan 2019 (29) (+) -Oni 2020 (38) (+) (SUD) -Philips 2015 (35) (+) -Rothera 2011 (57) (+) -Savory 2022 (21) (+)
	***Barriers to access care***		
	Lack of time/workload		-Edge 2010 (31) (–) -Noonan 2019 (29) -Oni 2020 (38) (–) (SUD)
	***Enablers to access care***		
	Multidisciplinary work		-McCauley 2011 (39) (+) -Oni 2020 (38) (+)
	Access to reactive specialist care		-Edge 2010 (31) (+)
	Specialist team		-Oni 2020 (38) (+) -Ross-Davie 2006 (51) (+) (attitude toward referral)
	Supervision		-McCauley 2011 (39) (+)
**Parents level**	***Stigma***		
	Negative attitudes toward help-seeking		-Oni 2020 (38) (–) (SUD)
	Anticipated stigma		-Oni 2020 (38) (–) (SUD)
	***Insufficient language proficiency/learning difficulties***		-Savory 2022 (21) (–)
**Interaction level**	***Person-centered care***		-Oni 2020 (38) (+) (SUD)

FOC, Fear of Childbirth; NS, non significant; PMH, Perinatal Mental Health; PMHC, Perinatal Mental Health Care; SUD, Substance Use Disorder.

**(+)**, positive relationship; (–), negative relationship.

**Table 6:** Factors influencing decisions about support.

	Influencing factors	Support	
		Significance	Direction of the relationship
**Provider level**	***Background***		
	Age	-Magdalena 2020 (9) (attitudes toward usefulness of support groups)	-Magdalena 2020 (9) (–) (attitudes toward usefulness of support groups)
	Education level	-Jones 2012b (37) (NS)	
	Years of experience	-Magdalena 2020 (9) (attitudes toward usefulness of support groups) - Jones 2012b (37) (NS)	-Magdalena 2020 (9) (–) (attitudes toward usefulness of support groups
	Type of practice/work context	- Jones 2012b (37) (NS)	
	Personal interest in PMH	-Fontein-Kuipers, 2014 (42)	-Fontein-Kuipers, 2014 (42) (+) -Jarrett 2015 (43) (+) -Phillips 2015 (35) (+)
	Frequent work with parents with mental health problems		-McCauley 2011 (39) (+)
	Positive experience with parents with mental health problems		-McCauley 2011 (39) (+)
	***Knowledge***		
	Signs and symptoms	-Noonan 2018 (28) (+) -Noonan 2019 (29) (+).	-Asare 2022 (47) (+) -McCauley 2011 (39) (+) -Noonan 2018 (28) (+) -Noonan 2019 (29) (+) -Ross-Davie 2006 (51) (+) -Shahid Ali 2023 (44) (+) -Rothera 2011 (57) (+) -Savory 2022 (21) (+) (SMI)
	Risk factors		-Salomonsson 2010 (59) (+) (FoC)
	Consequences		-Nyberg 2010 (60) (+) (PTSD after childbirth) -Salomonsson 2010 (59) (+) (FoC)
	Treatment options		-Bye 2018 (36) (+) -Jones 2012b (37) (+)
	Available resources		-Bye 2018 (36) (+)
	Referral pathways		-Bye 2018 (36) (+) -Phillips 2015 (35) (+)
	Cultural aspects		-Phillips 2015 (35) (+)
	***Skills***		-Edge 2010 (31) (+) -Ross-Davie 2006 (51) (+) -Shahid Ali 2023 (44) (+) -Stewart 2002 (30) (+)
	Interviewing skills		-McCauley 2011 (39) (+)
	Communication skills		-Nyberg 2010 (60) (+) (PTSD after childbirth)
	Distress management		-Asare 2022 (47) (+)
	Listening/Non-judgmental and supportive approach		-Asare 2022 (47) (+) -McCauley 2011 (39) (+) -Nyberg 2010 (60) (+) (PTSD after childbirth) -Whitehead 2019 (32) (+) (SUD)
	***Attitudes***		
	Negative attitudes toward PMHP		-Savory 2022 (21) (SMI)
	Toward screening		-Asare 2022 (47) (+) -Jarrett 2015 (43) (–)
	Toward screening tools		
	Perceived role in PMHC		-Jarrett 2015 (43) (+) (for SMI) -McCauley 2011 (39) (+) -Rothera 2011 (57) (+) (attitudes toward referral and management) -Ross-Davie 2006 (51) (+) -Stewart 2002 (30) (+)
	Stigma		
	- Perceived dangerousness		-Jarrett 2015 (43) (+) -McCauley 2011 (39) (–) -Philips, 2015 (35) (–) -Ross-Davie 2006 (51) (–)
	- Perceived inability to provide adequate childcare		-Philips, 2015 (35) (–)
	- Perceived inability to engage in antenatal care		-Shahid Ali 2023 (44) (–)
	- Cultural bias		-Asare 2022 (47) (–)
	Attitude toward support/management	-Fontein-Kuipers, 2014 (42) (+)	-Fontein-Kuipers, 2014 (42) (+)
	- Pharmacological treatment		-Jones 2012b (37) (–)
	***Self-efficacy in providing PMHC***		-Edge 2010 (31) (+) -Jarrett 2015 (43) (+) (SMI) -Jones 2012a (55) (+) -Savory 2022 (21) (+) -Stewart 2002 (30) (+)
	***Emotional impact on the midwife/discomfort***		-Salomonsson 2010 (59) (–) (FoC)
**Organizational level**	***Local policy***		-Asare 2022 (47) (+)
	Routine use of screening tools		-Asare 2022 (47) (+)
	***Continuity of care***		-Edge 2010 (31) (+)
	Available resources		-Asare 2022 (47) (+)
	Clear referral pathway		-Asare 2022 (47) (+) -Edge 2010 (31) (+) -Rothera & Oates 2011 (57)
	***Barriers to access care***		
	Lack of time/workload		-Asare 2022 (47) (–) -Edge 2010 (31) (–) -Jones 2012b (37) (–) -McCauley 2011 (39) (–) -Ross-Davie 2006 (51) (–)
	Long waiting times for a visit (insufficient number of staff members)		-Asare 2022 (47) (–)
	Lack of confidentiality		-Asare 2022 (47) (–)
	***Enablers to access care***		
	Access to reactive specialist care		-Dubreucq 2019 (34) (+) -Edge 2010 (31) (+)
	Support from team members		-Jones 2012b (37) (+)
	Supervision		-Nyberg 2010 (60) (+) (PTSD after childbirth)
**Parents level**	***Stigma***		
	Negative attitudes toward help-seeking		-Jones 2012b (37) (–)
	Experienced stigma		-Edge 2010 (31) (–) -Nyberg 2010 (60) (+) (PTSD after childbirth)
	***Negative experiences with HPs***		-Edge, 2010 (31) (–) -Nyberg 2010 (60) (+) (PTSD after childbirth)
**Interaction level**	***Building a trusting relationship***		-Edge 2010 (31) (+)
	***Person-centered care***		-Edge 2010 (31) (+)

FOC, Fear of Childbirth; NS, non significant; PMH, Perinatal Mental Health; PMHC, Perinatal Mental Health Care; PTSD, Post Traumatic Stress Disorder; SMI, Serious Mental Illness; SUD, Substance Use Disorder.

**(+)**, positive relationship; (–), negative relationship.

### 2.5 Quality assessment

Quality assessment was realized using the Mixed Methods Appraisal Tool (MMAT) (61). MMAT is a validated instrument to assess the methodological quality of qualitative, randomized controlled trials, non-randomized trials, descriptive studies, and mixed methods studies. It is comprised of five 5-item subscales assessing different aspects of quality (e.g. appropriateness of the selected design/methods/measurements, integration of quantitative and qualitative parts for mixed-methods studies). Two researchers (MD and JD) independently assessed methodological quality using the MMAT and extracted MMAT scores for each article. Discrepancies were resolved through consensus. The MMAT overall quality score and detailed scores are provided in **Supplementary Tables 4**, **5**. The study protocol was registered on PROSPERO on November 1, 2021 (CRD42021285926).

## 3 Results

**Figure 1:**
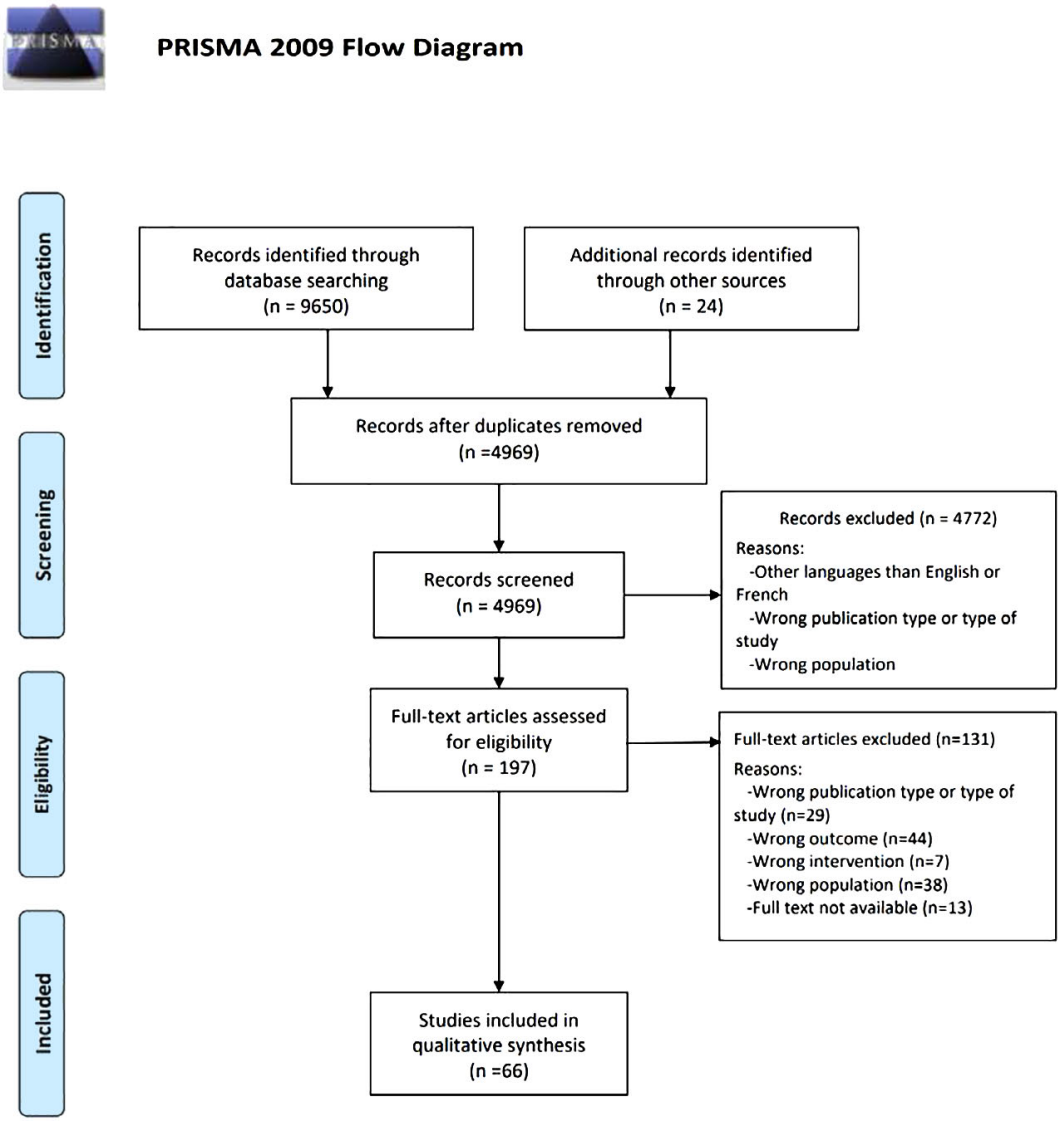
PRISMA diagram.

Of the 9650 articles found during searches from inception to June 26^th^ 2023, 4969 references remained after removing all duplicates. Based on titles and abstracts, 4772 papers were excluded for lack of relevance. Our search strategy yielded 197 full-text articles. After conducting a full-text analysis of all these papers, we ended up with 66 relevant papers (47 on knowledge, skills or attitudes and 19 on training programs; PRISMA diagram on **Figure 1**).

### 3.1 Study characteristics

The characteristics of the 66 included studies are presented on **Tables 7**, **8**. Most studies were conducted in high-income countries (89.4%) and published after 2015 (50%). Study designs were quantitative (n=33; 50%), qualitative (n=22; 33.3%) or mixed-methods (n=11; 16.7%). Samples included qualified midwives (n=37; 56.0%), qualified midwives and other perinatal health providers (n=17; 25.8%) and student midwives (n=11; 16.7%). Qualified midwives had a variable level of training in PMH ranging from none to 90% (specified in 24 studies; most covered topics: general information about PMH and PMHPs; least covered topics: interviewing/counseling skills, psychopharmacology and suicide risk assessment). Eight studies (12.1%) reported on midwives’ mental health nursing experience (ranging from 0.8% to 30%) or placement experience in a mental health setting or a mother-baby unit during their studies (ranging to 9% to 23.2%). Four studies (6%) mentioned family or personal experience of mental health problems ranging from 25% to 66.3%. Most studies covered the entire perinatal period (n=44; 66.7%) and reported on PMHPs (n=32; 48.5%). The definition of PMHPs was highly variable across the studies (e.g. inclusion of conditions usually not considered as PMHPs, such as schizophrenia, bipolar disorder, personality disorders, self-harm, suicide eating disorders or SUD in 16 studies; definition restricted to anxiety, depression, postpartum psychosis and/or posttraumatic stress disorder in 9 studies; unspecified in 7 studies). One third of the included studies used validated instruments to assess outcomes (n=16; 36.4%). Five studies (7.6%) investigated the influence of cultural aspects on the detection and management of PMHPs.

**Table 7:** Research characteristics of the 66 studies included in the review.

Characteristic	Midwives’ knowledge, skills, attitudes (n=47) [n (%)]	Training programs (n=19) [n (%)]	Total (n=66) [n (%)]
Publication date			
1995-2005	2 (4.3%)	3 (15.8%)	5 (7.6%)
2006-2015	22 (46.8%)	6 (31.6%)	28 (42.4%)
2016-2023	23 (48.9%)	10 (52.6%)	33 (50%)
Region of study			
High-income countries	43 (91.5%)		59 (89.4%)
North America	*1 (2.3%)*	*3 (18.7%)*	4 (6.8%)
Western Europe	*30 (69.8%)*	*9 (56.3%)*	39 (66.1%)
Australia	*11 (25.6%)*	*2 (12.5%)*	13 (22.0%)
Others	*1 (2.3%)*	*2 (12.5%)*	3 (5.1%)
Low to middle income countries	4 (8.5%)	3 (15.8%)	7 (10.6%)
Study design			
Cross-sectional	47 (100%)	10 (52.6%)	57 (86.4%)
Longitudinal	0	9 (47.4%)	9 (13.6%)
Study sites			
Single site	20 (42.6%)	16 (84.2%)	36 (54.5%)
Multiple sites	19 (40.4%)	3 (15.8%)	22 (33.3%)
Survey (online or postal)	8 (17.0%)	0	8 (12.1%)
Type of study			
Quantitative study	22 (46.8%)	11 (58.0%)	33 (50%)
Qualitative study	18 (38.3%)	4 (21.0%)	22 (33.3%)
Mixed-method study	7 (14.9%)	4 (21.0%)	11 (16.7%)
***Type of quantitative design***	*n=29*	*n=15*	*n=44*
Randomized Controlled Trials	0	1 (6.7%)	1 (2.2%)
Control group	0	2 (13.3%)	2 (4.5%)
Descriptive	29 (100%)	13 (86.7%)	42 (93.3%)
Type of providers			
Midwives	29 (61.7%)	8 (42.1%)	37 (56.0%)
Student midwives	5 (10.7%)	6 (31.6%)	11 (16.7%)
Midwives and student midwives	1 (2.1%)	0	1 (1.5%)
Midwives and other providers	12 (25.5%)	5 (26.3%)	17 (25.8%)
***Peripartum mental health problems***	*n=26*	*n=6*	*n=32*
Broad definition of perinatal mental health problems**	12 (46.1%)	4 (66.6%)	16 (50%)
Narrow definition of perinatal mental health problems	8 (30.8%)	1 (16.7%)	9 (28.1%)
Unspecified	6 (23.1%)	1 (16.7%)	7 (21.9%)
Condition(s) covered***			
Peripartum mental health problems	26 (55.3%)	6 (31.6%)	32 (48.5%)
Depression	16 (34.0%)	10 (52.6%)	26 (39.4%)
Suicide	1 (2.1%)	2 (10.5%)	3 (4.5%)
Anxiety	3 (6.4%)	4 (21.1%)	7 (24.2%)
Post-traumatic stress disorder	2 (4.3%)	0	2 (3.0%)
Fear of childbirth	3 (6.4%)	0	3 (4.5%)
Serious mental illness	4 (8.5%)	4 (21.1%)	8 (12.1%)
Bipolar Disorder	0	0	0
Schizophrenia	2 (4.3%)	0	2 (3.0%)
Postpartum psychosis	2 (4.3%)	0	2 (3.0%)
Eating disorder	1 (2.1%)	1 (5.3%)	2 (3.0%)
Substance use disorders	5 (10.7%)	4 (21.1%)	9 (13.6%)
Autism	0	0	0
Timing of the peripartum period			
Pregnancy	8 (17.0%)	5 (26.3%)	13 (19.7%)
Postpartum	5 (10.6%)	4 (21.1%)	9 (13.6%)
Both antenatal and postpartum	34 (72.4%)	10 (52.6%)	44 (66.7%)
***Cultural aspects***	5 (10.6%)	0	5 (7.6%)
***Type of questionnaire*** *(quantitative and mixed method study only)*	*n=29*	*n=15*	*n=44*
Validated questionnaire	10 (34.5%)	6 (40.0%)	16 (36.4%)
Self-designed questionnaire	19 (65.5%)	9 (60.0%)	28 (63.6%)
Quality rating			
High	9 (19.1%)	2 (10.5%)	11 (16.7%)
Moderate	21 (44.7%)	4 (21.1%)	25 (37.9%)
Low	17 (36.2%)	13 (68.4%)	30 (45.4%)

** Inclusion of conditions usually not considered as PMHPs.

***total > 100% because some studies covered more than one condition.

**Table 8:** Research characteristics of the training programs included in the review.

Characteristic	Training programs (n=19) [n (%)]
Nature	
Initial training	6 (31.6%)
Continuous education	13 (68.4%)
Compulsory vs. elective training	
Compulsory training	5 (26.3%)
Elective training	3 (15.8%)
Unspecified	11 (57.9%)
Format	
In-person	8 (42.1%)
e-learning	4 (21.1%)
Mixed-format	3 (15.7%)
Unspecified	4 (21.1%)
Duration	
Less than one hour	2 (10.5%)
Less than one day	3 (15.8%)
Less than 3 days	4 (21.1%)
3 days or more	6 (31.5%)
Unspecified	4 (21.1%)
Contact with persons with lived experience*	
Co-construction	2 (10.5%)
Video or written testimonies	7 (36.8%)
None	12 (61.2%)

*total > 100% because some studies used co-construction and testimonies.

Of 15 studies reporting on a training program using a quantitative or a mixed-methods design, three used a waiting-list control group (20%; one randomized controlled trial (RCT)) and 13 (86.7%) were uncontrolled. Sample size was small in most studies (< 50 participants; n=9 studies). Nine studies (47.3%) reported contact with persons with lived experience when designing their training program. The training programs were heterogeneous in nature (initial training, n=6, 31.6%; continuous education, n=13, 68.4%), type, format and duration (ranging from 2 minutes to a fifteen-week module). All studies assessed training outcomes either immediately after (n=15; 79%) or up to 3 months after the intervention is delivered (n=4; 21%).

### 3.2 Quality assessment

The overall assessment score ranged from low (n=30, 45.4%; n=13, 68.4% for training programs) to high (n=11, 16.7%; n=2, 10.5%). For quantitative or mixed-methods studies, the reasons were convenience sampling (n=61 studies, 92.4%), sample size, low response rate (n=18 studies > 60%), limited use of validated outcome measures (36.4%), use of self-reported measures, absence or short duration of the follow-up period, limited integration of the results in mixed-methods studies and lack of controlled/RCT studies to evaluate the effectiveness of training programs. For qualitative studies, the reasons were interpretation bias (e.g. no investigator triangulation, the data being analyzed by only one researcher), absence of data saturation and lack of reflexivity.

### 3.3 Narrative review

Many studies found that midwives felt ill equipped to care for parents with PMHPs [e.g. ranging from 69.2% of 815 midwives in Jones et al., 2011 (17) to 82.2% of 157 midwives in Noonan et al., 2018 (28)]. The reasons included insufficient initial training/continuous education on PMH (n=2 studies), perception that PMH assessment is not part of their role (n=2 studies), lack of knowledge about the detection, referral and management of PMHPs (n=12 studies). Compared with other perinatal health providers (GPs, health visitors, maternal child health nurses; n=11 studies), midwives had lower knowledge on PMH (n=2), felt less confident in the detection, referral or management of PMHPs (n=3) and had more negative attitudes toward their role in perinatal mental healthcare (PMHC) (57) or suicide prevention (40). Self-reported barriers to discuss PMH issues or self-reported interviewing skills did not differ between nurses and midwives (25). Student midwives’ knowledge, skills and attitudes in PMH did not clearly differ from those of qualified midwives (n=5 studies). On the job experience, learning from peers and attending to workshops/conferences were midwives’ main sources of knowledge (n=3 studies).

The factors positively associated with knowledge about PMHPs included the perception to be well equipped to provide PMHC (66.7% significance), previous training in PMH (50% significance), younger age (17), shorter work experience in general and as a midwife (20% significance), frequent contact with parents with PMHPs (50% significance) and type of practice (33.3% significance). Mental health nursing experience was positively associated with the perception to be well equipped to provide PMHC, but not with higher knowledge about PMH (8). No significant association was found between confidence in providing PMHC and other factors [e.g. age, personal experience of mental health problems, frequent contact with parents with PMHPs (29)], except for PMH education and case identification (8). Compared with suicide risk assessment and other conditions (e.g. postpartum psychosis, SMI, eating disorders or posttraumatic stress disorder; n=4 studies), midwives reported higher knowledge, better skills and more confidence in detecting and managing perinatal depression and anxiety. Midwives felt in general ill equipped to care for postpartum psychosis, eating disorders, posttraumatic stress and SMI (n=10 studies) and reported ambivalent or negative attitudes toward parents with these conditions (n=7 studies). Knowledge about PMHPs varied according to the assessment method [i.e. higher self-report knowledge than researcher-rated knowledge (19, 43)] and the timing of perinatal period (i.e. higher in the postpartum than during pregnancy, n=5 studies).

#### 3.3.1 Detection/screening

The practices and policies around screening for PMHPs varied across studies. There was a considerable overlap between the factors influencing the decision to screen, refer and support parents with PMHPs. Midwives’ attitudes toward their role in PMHC (e.g. personal interest in PMHPs and perception that it is part of their role) played a central role in decision-making about opening discussions about PMH (n=12 studies), referral (42, 57) and support parents with PMHPs (n=6). Cultural aspects and stigma toward parents with ethnic minority background (e.g. underestimation of depression and suicide risks) impacted midwives’ ability to detect and manage PMHPs and parents’ maternity care experiences (n=4 studies). Other common factors included lack of knowledge about PMHPs (n=20 studies), referral pathways (n=8) and treatment options (n=10), lack of time/clear referral pathways (n=22) and stigma related to preexisting mental health problems/SMI (n=8).

Midwives considered routine universal screening as useful in two studies (5, 56). Facilitators included self-efficacy in screening (n=10 studies), person-centered care (n=3), the presence of a specialist team (n=2 studies) and mandatory routine screening (n=2). Barriers to screening included longer work experience (42), lack of knowledge about screening tools (n=11 studies), local/national guidelines on screening (ranging from 12.8% to 53%, n=4 studies), and negative attitudes toward the use of formal screening tools (n=12 studies). The relationship between personal/family experience of PMHPs was either positive [e.g. reduces stigma and allows to relate with parents (29)] or negative (45). For student midwives, the presence of specialist midwives was both a facilitator [e.g. provides referral options and placement opportunities (50)] and a barrier to screening [e.g. perception that it is not part of their role (43)]. Of note, specialist midwives reported to lack confidence in opening discussions about PMH and to lack knowledge about SMI (21).

The reasons underlying negative attitudes toward the use of formal screening tools included perceiving the questions as intrusive (n=3 studies), not clearly understanding the purpose of doing so (n=3 studies), inexperience in conducting assessment and feeling compelled to undertake it as a standardized survey (23), the fear of “not doing it right” (n=2) and discomfort when disclosure occurs (n=7 studies). Some studies reported a flexible use of screening tools (e.g. modified wording or timing of the questions; n=4 studies) and one study outlined the importance of person-centered care in conducting assessment (23). Conversely, midwives who lacked clarity about their role in PMHC reported feelings of inadequacy resulting in a non-flexible use of screening tools and a distant and superficial manner of asking questions (23). Midwives reported to feel more comfortable in opening discussions about PMH during follow-up visits compared with the booking appointment (n=5 studies). Alternatives to formal screening included assessing previous psychiatric history/current symptoms (28), using general open-ended questions (n=5 studies), behavioral observation (n=4 studies) and labor debriefing (46). Training needs covered knowledge about PMHPs (n=9 studies), screening tools (n=4 studies) and cultural issues and interviewing/distress management skills (n=10 studies).

#### 3.3.2 Referral/support

Midwives reported to feel confident in their ability to refer parents with PMHPs to other health providers including specialist mental health services (n=7 studies). The opposite was found for parents with postpartum psychosis, eating disorders or SMI. High self-reported confidence in referring parents to other providers did not in practice lead to a higher number of referrals (37). The proportion of midwives indicating to feel confident in supporting parents with PMHPs in self-report questionnaires ranged from 34% to 53% (n=5 studies). Accurate case identification (9), an established diagnosis of PMHP (53) and parents’ preferences (53) influenced decision-making about referral. Other factors included the intention to collaborate with other providers (n=2) or conversely a lack of trust/a reluctance to disclose sensitive information to other providers (n=3 studies).

#### 3.3.3 Training outcomes

All training programs reported improved self-rated knowledge, skills, attitudes and confidence in screening, referring and supporting parents with PMHPs (n=19). Few significant positive training effects were reported due to small-sized samples and lack of controlled/RCT studies. Results included positive effects on empathic communication skills (62, 63), case identification (64, 65) and the detection of PMHPs in maternity wards (66–68). Contrasted results were found on the number of referrals [n=2 studies; 50% significance; positive effect on self-reported referrals in Pearson et al. (2019) (69) and no significant effect in Wickberg et al. (2005, 70)]. No significant effects were found on depressive symptoms (70) and attitudes toward providing psychological support to parents with PMHPs (63). Participants’ satisfaction rates were high, the insight provided by parents with lived experience of PMHP being determinant for student midwives (n=4 studies). Barriers included an excessive workload (71) and for student midwives, elective participation and late delivery within midwifery studies (72). No difference related to the format of the intervention was reported.

## 4 Discussion

To our knowledge, this systematic review of 66 studies is one of the first exploring both the training needs in PMH identified by student midwives and midwives and the training programs designed for this population. Overall, a main finding of this systematic review is that although detection, referral and support of parents with PMHPs are part of the essential competencies for midwifery practice according to the ICM (2019) (6), their effective translation into routine clinical practice may depend on midwives’ understanding of their role in PMHC, i.e. finding meaning in opening discussions about PMH with all parents and the perception that this is part of their routine clinical duties. This suggests that this factor should be targeted by raining interventions aiming at improving detection and management of PMHPs, above and beyond knowledge, confidence, and skills.

Extending the findings of previous reviews (7, 10, 11), we found that although most midwives consider they have a role in PMHC (this aligning with ICM essential competencies for midwifery practice; 2019 (6)), their understanding of that role remains often unclear. Several potential explaining factors have been identified. First, while this topic may be central for a meaningful engagement into providing PMHC, only a few training programs explored the role of midwives in PMHC (71, 73). Second, there is a view - in particular in student midwives - that addressing PMH needs is less a priority than addressing physical health needs and that other providers should assume this responsibility (31, 35, 39, 43, 50, 52). The interaction between this view, mental illness stigma and racism toward parents with ethnic minority background contributed to poorer maternity experiences and under-detection of PMHPs (19, 35, 73).

Third, some midwives consider their role as limited to assessing PMH and wellbeing and as appropriate, referring to other health providers (9, 18, 55, 57, 58), whereas others have a broad perception of their role that include providing support, psychoeducation and with adequate training counseling interventions (21, 24, 25, 42). Recent meta-analyses showed positive effects of midwife-led counseling on anxiety and depressive symptoms after at least 3 days of training (14, 74). This concurs with recent calls for a better integration of mental health and perinatal health care and an extension of the scope of midwifery practice to include strengths-based case management and psychological interventions for parents with PMHPs (50, 75–77). Given there is some degree of difference between midwives’ perception of their role in PMHC and what is required as essential competencies for midwifery practice (ICM, 2019) (6), an explicit focus on midwives’ role in PMHC should be made in initial and continuous midwifery education (72, 73, 76, 78). Fourth, most student midwives, midwives and specialist midwives reported negative attitudes toward parents with suicide ideations, postpartum psychosis and SMI (21, 35, 40, 43, 57, 79). Aligning with this, Hawthorne et al. (2020) (79) found that student midwives had more negative attitudes toward persons with mental illness compared with mental health nursing students. However, other studies reported that midwives consider caring for parents with these conditions as part of their role but felt ill equipped to do so and expressed the need for additional training (8, 28, 29, 34, 39, 49).

### 4.1 Implications for training interventions

While the need to improve midwives’ initial and continuous education in PMH is now well established (7, 10), student midwives, midwives and even specialist midwives continue reporting to feel ill prepared to care for parents with PMHP in particular in case of co-occurring SMI (9, 21, 24, 33, 34, 36). Moreover, the proportion of midwives who received education in PMH - in particular in topics such as mental health/suicide risk assessment - remains consistently low. Given suicide is the leading cause of maternal mortality in the 1^st^ year postpartum in high-income countries, this is concerning (1, 80).

Aligning with previous research (7, 10, 11), this systematic review found that education/training programs had positive effects on proximal outcomes (e.g. midwives’ knowledge, skills, attitudes and confidence in providing PMHC) and contrasted effects on distal outcomes (e.g. screening in maternity wards, the number of referrals or depressive symptoms). This could be related to methodological bias (e.g. lack of RCT or quasi-experimental studies). There is a need for high-quality studies on interventions designed following the Medical Research Council framework for complex interventions (81), which proposes among other core elements to: 1) take into account the context of delivery; 2) use a clear theoretical basis (e.g. how the intervention is expected to produce positive effects and under which conditions) and; 3) promote a meaningful engagement of persons with lived experience among other relevant stakeholders.

According to Wadephul et al. (2018) (82) framework for assessing midwifery practice in PMH, knowledge, confidence, attitudes and organizational factors influence midwives’ ability to detect and manage PMHPs. However, higher knowledge about PMH does not necessarily translate into higher confidence in providing PMHC and the opposite (8). As reported in one of the articles included in this review (42) and aligning with the theory of planned behavior (82), additional factors such as individual values, e.g. personal interest in PMH, and behavioral intent (e.g. the intention to open discussions about PMH) could influence detection and decision-making about referral and support in PMHPs and thus be relevant for midwifery education.

To improve midwives’ engagement into PMHC, training programs should put PMH in context (e.g. the positive outcomes that could be achieved with appropriate support) before covering topics related to specific knowledge or skills (5, 38, 49, 50, 53, 54). Instead of focusing only on biomedical aspects (e.g. the signs, risk factors, consequences and treatments of PMHPs), programs should propose a continuum approach of PMH that covers the positive aspects of the person’s life including wellbeing and personal recovery (83–86).

Extending the findings of previous reviews (7, 10, 11), training programs should target student midwives, midwives and specialist midwives and cover interviewing and distress management skills with a focus on specific aspects (e.g. opening discussions without feeling intrusive, using flexibly screening tools and reacting in case of a positive answer) (5, 21, 38, 45, 49, 50, 53, 54). In addition, training programs should include clinical supervision by mental health providers during and after intervention delivery (14). Future studies should include a longer follow-up period, as the embedding of practice change requires a minimum of nine months after the intervention is delivered (87).

Finally, while contact with persons with lived experience is one of the most effective strategies to reduce mental illness stigma in the general public and in frontline health providers (88, 89), this review found a very low proportion of training programs that engaged persons with lived experience in the conception and delivery of the intervention. Initial and continuous midwifery education curriculums on PMH should involve persons with lived experience - co-design and co-intervention - and include content about personal recovery/person-centered care (72, 73, 81, 84, 90–92).

### 4.2 Limitations

There are limitations. First, despite a growing number of published studies on midwives’ training needs in PMH and training interventions designed for this population (n=66 studies in this review vs. n=22 (7); n=17 (10); n=43 (11);), the quality of the included studies remains low to moderate, a concerning finding given the clinical relevance of this topic that is also a considerable limitation. Among other methodological bias, the absence of a clear theoretical basis for designing interventions (81), the small or unjustified sample sizes, the lack of RCT/quasi-experimental studies, the absence of control groups (or active comparators in controlled studies) and the absence or short duration of follow-up makes unclear whether interventions have positive effects on proximal or distal outcomes. Future high-quality studies on this topic are therefore needed. Despite these limitations, the inclusion of quantitative, qualitative and mixed-methods studies provided a complete synthesis of the available evidence and consistent messages emerged across studies. Second, relevant studies may have been missed since we excluded studies published in other languages than English or French and did not include the grey literature in our searches.

## 5 Conclusion

This review generated novel insights to inform initial and continuous midwifery education curriculums on PMH (e.g. co-design with persons with lived experience, focus on midwives’ understanding on their role in PMHC or inclusion on content on person-centered care).

## Author contributions

MD: Conceptualization, Formal analysis, Writing – original draft. CD: Writing – review & editing. ML: Conceptualization, Writing – review & editing. WB: Conceptualization, Data curation, Methodology, Writing – review & editing. CM: Writing – review & editing. JD: Conceptualization, Formal analysis, Project administration, Supervision, Validation, Writing – original draft.

## Supplementary material

Supplementary Table 1 Search strategy, search terms and syntax.

Supplementary Table 2 List of excluded studies.

Supplementary Table 3 List of included studies.

Supplementary Table 4 Characteristics of included studies about midwives' knowledge, skills and attitude.

Supplementary Table 5 Characteristics of included studies about midwives' needs for peripartum mental health training program.

Supplementary Table 6 List of abbreviations.
