# Supplementary material for: A systematic review of midwives’ training needs in perinatal mental health and related interventions
Source: Front Psychiatry. 2024 Apr 22;15:1345738. doi: 10.3389/fpsyt.2024.1345738 (PMC11071341; doi:10.3389/fpsyt.2024.1345738)
Supplement: Supplementary Table 1 — Search strategy, search terms and syntax. [file Table_1.docx]

**Supplementary table 1. Search strategy**

| **Database searched** | **Platform** | **Years of coverage** | **Records** | **Records after duplicates removed** |
| --- | --- | --- | --- | --- |
| Medline ALL | Ovid | 1946 - Present | 2283 | 2269 |
| Embase | Embase.com | 1971 - Present | 2770 | 1362 |
| Web of Science Core Collection* | Web of Knowledge | 1975 - Present | 1891 | 529 |
| Cochrane Central Register of Controlled Trials ** | Wiley | 1992 - Present | 419 | 40 |
| CINAHL*** | EBSCOhost | 1982 - Present | 1789 | 644 |
| PsycINFO | Ovid | 1806 - Present | 498 | 101 |
| **Total** | | | **9650** | **4945** |

*Science Citation Index Expanded (1975-present) ; Social Sciences Citation Index (1975-present) ; Arts & Humanities Citation Index (1975-present) ; Conference Proceedings Citation Index- Science (1990-present) ; Conference Proceedings Citation Index- Social Science & Humanities (1990-present) ; Emerging Sources Citation Index (2005-present)

** Manually deleted abstracts from trial registries

***Limited to Academic Journals

No other database limits were used than those specified in the search strategies

The search strategies for Embase Medline, CINAHL and PsycINFO used relevant thesaurus terms from their respective thesauri (for example Emtree and Medical Subject Headings)

**embase.com**

(midwife/exp OR 'midwifery student'/de OR 'obstetrical nursing'/exp OR obstetrician/de OR obstetrics/de OR (midwif* OR midwive* OR birth NEAR/2 attendant* OR obstetrical* OR obstetric*):ab,ti,kw) AND ('mental health'/exp OR 'mental disease'/exp OR depression/exp OR psychiatry/exp OR psychoeducation/de OR 'mental health service'/exp OR 'mental health care'/exp OR (((mental* OR psycholog*) NEAR/3 (health OR care OR healthcare OR well-being OR wellbeing OR disease* OR disorder*)) OR ((behav* OR anxiety) NEAR/3 (disease* OR disorder*)) OR (eating NEAR/3 disorder*) OR depression* OR psychosis OR psychiatr* OR psychoeducat* OR psycho-educat* OR psychsomatic* OR autism* OR adhd OR (attention NEAR/3 deficit*) OR pdd-nos OR Asperger* OR suicid* OR automutilat* OR self-harm* OR phobia OR (fear NEAR/3 (childbirth* OR birth*)) OR tocophobi* OR tokophobi*):ab,ti,kw) AND (education/de OR 'continuing education'/de OR curriculum/de OR 'curriculum development'/de OR 'education program'/de OR 'in service training'/de OR 'interprofessional education'/de OR 'interdisciplinary education'/de OR 'vocational education'/de OR skill/de OR competence/exp OR 'midwifery student'/de OR 'program evaluation'/de OR (education* OR training OR curricul* OR skill* OR competenc* OR student* OR (program* NEAR/6 (evaluat* OR develop* OR implement*))):Ab,ti,kw) NOT [conference abstract]/lim AND [english]/lim NOT ([animals]/lim NOT [humans]/lim)

**Medline**

(Midwifery/ OR Nurse Midwives/ OR Obstetric Nursing / OR exp Delivery, Obstetric/ OR Obstetrics/ OR (midwif* OR midwive* OR birth ADJ2 attendant* OR obstetrical* OR obstetric*).ab,ti,kf.) AND (Mental Health/ OR exp Mental Disorders/ OR Depression/ OR exp Depressive Disorder/ OR Adjustment Disorders/ OR exp Mental Health Services/ OR (((mental* OR psycholog*) ADJ3 (health OR care OR healthcare OR well-being OR wellbeing OR disease* OR disorder*)) OR ((behav* OR anxiety or depressive) ADJ3 (disease* OR disorder*)) OR (eating ADJ3 disorder*) OR depression* OR psychosis OR psychiatr* OR psychoeducat* OR psycho-educat* OR psychsomatic* OR autism* OR adhd OR (attention ADJ3 deficit*) OR pdd-nos OR Asperger* OR suicid* OR automutilat* OR self-harm* OR phobia OR (fear ADJ3 (childbirth* OR birth*)) OR tocophobi* OR tokophobi*).ab,ti,kf.) AND (education.fs.OR exp Education, Continuing/ OR exp Curriculum/ OR exp Program Evaluation/ OR Inservice Training/ OR Interprofessional Education/ OR exp Professional Competence/ OR (education* OR training OR curricul* OR skill* OR competenc* OR student* OR (program* ADJ6 (evaluat* OR develop* OR implement*))).ab,ti,kf.) NOT (news OR congres* OR abstract* OR book* OR chapter* OR dissertation abstract*).pt.AND english.la. NOT (exp animals/ NOT humans/)

**Cochrane**

((midwif* OR midwive* OR birth NEAR/2 attendant* OR obstetrical* OR obstetric*):ab,ti) AND ( (((mental* OR psycholog*) NEAR/3 (health OR care OR healthcare OR well-being OR wellbeing OR disease* OR disorder*)) OR ((behav* OR anxiety) NEAR/3 (disease* OR disorder*)) OR (eating NEAR/3 disorder*) OR depression* OR psychosis OR psychiatr* OR psychoeducat* OR psycho-educat* OR psychsomatic* OR autism* OR adhd OR (attention NEAR/3 deficit*) OR pdd-nos OR Asperger* OR suicid* OR automutilat* OR self-harm* OR phobia OR (fear NEAR/3 (childbirth* OR birth*)) OR tocophobi* OR tokophobi*):ab,ti) AND ((education* OR training OR curricul* OR skill* OR competenc* OR student* OR (program* NEAR/6 (evaluat* OR develop* OR implement*))):Ab,ti) NOT "conference abstract":pt

**Web of Science**

TS=(((midwif* OR midwive* OR birth NEAR/2 attendant* OR obstetrical* OR obstetric*)) AND ( (((mental* OR psycholog*) NEAR/2 (health OR care OR healthcare OR well-being OR wellbeing OR disease* OR disorder*)) OR ((behav* OR anxiety) NEAR/2 (disease* OR disorder*)) OR (eating NEAR/2 disorder*) OR depression* OR psychosis OR psychiatr* OR psychoeducat* OR psycho-educat* OR psychsomatic* OR autism* OR adhd OR (attention NEAR/2 deficit*) OR pdd-nos OR Asperger* OR suicid* OR automutilat* OR self-harm* OR phobia OR (fear NEAR/2 (childbirth* OR birth*)) OR tocophobi* OR tokophobi*)) AND ((education* OR training OR curricul* OR skill* OR competenc* OR student* OR (program* NEAR/5 (evaluat* OR develop* OR implement*))))NOT ((animal* OR rat OR rats OR mouse OR mice OR murine OR dog OR dogs OR canine OR cat OR cats OR feline OR rabbit OR cow OR cows OR bovine OR rodent* OR sheep OR ovine OR pig OR swine OR porcine OR veterinar* OR chick* OR zebrafish* OR baboon* OR nonhuman* OR primate* OR cattle* OR goose OR geese OR duck OR macaque* OR avian* OR bird* OR fish*) NOT (human* OR patient* OR women OR woman OR men OR man))) NOT DT=(Meeting Abstract OR Meeting Summary) AND LA=(English)

**Cinahl**

(MH Midwifery+ OR MH Nurse Midwives OR MH Obstetric Nursing OR MH Delivery, Obstetric+ OR MH Obstetrics ORTI(midwif* OR midwive* OR birth N2 attendant* OR obstetrical* OR obstetric*)ORAB(midwif* OR midwive* OR birth N2 attendant* OR obstetrical* OR obstetric*)) AND (MH Mental Health OR MH Mental Disorders+ OR MH Depression OR MH Depressive Disorder+ OR MH Adjustment Disorders OR MH Mental Health Services+ OR TI(((mental* OR psycholog*) N2 (health OR care OR healthcare OR well-being OR wellbeing OR disease* OR disorder*)) OR ((behav* OR anxiety or depressive) N2 (disease* OR disorder*)) OR (eating N2 disorder*) OR depression* OR psychosis OR psychiatr* OR psychoeducat* OR psycho-educat* OR psychsomatic* OR autism* OR adhd OR (attention N2 deficit*) OR pdd-nos OR Asperger* OR suicid* OR automutilat* OR self-harm* OR phobia OR (fear N2 (childbirth* OR birth*)) OR tocophobi* OR tokophobi*)OR AB(((mental* OR psycholog*) N2 (health OR care OR healthcare OR well-being OR wellbeing OR disease* OR disorder*)) OR ((behav* OR anxiety or depressive) N2 (disease* OR disorder*)) OR (eating N2 disorder*) OR depression* OR psychosis OR psychiatr* OR psychoeducat* OR psycho-educat* OR psychsomatic* OR autism* OR adhd OR (attention N2 deficit*) OR pdd-nos OR Asperger* OR suicid* OR automutilat* OR self-harm* OR phobia OR (fear N2 (childbirth* OR birth*)) OR tocophobi* OR tokophobi*)) AND (MH Education, Continuing+ OR MH Curriculum+ OR MH Program Evaluation+ OR MH Inservice Training OR MH Interprofessional Education OR MH Professional Competence+ OR TI(education* OR training OR curricul* OR skill* OR competenc* OR student* OR (program* N5 (evaluat* OR develop* OR implement*)))OR AB(education* OR training OR curricul* OR skill* OR competenc* OR student* OR (program* N5 (evaluat* OR develop* OR implement*)))) AND LA (English) NOT (MH animals+ NOT MH humans+)NOT (MH News OR MH Abstracts OR MH Books+)

**Psycinfo**

(Midwifery/ or exp "Labor (Childbirth)"/ or Obstetrics/ or (((midwif* or midwive* or birth) adj2 attendant*) or obstetrical* or obstetric*).ab,ti.) and (Mental Health/ or exp Mental Disorders/ or Depression/ or exp Major depression/ or Adjustment Disorders/ or exp Mental Health Services/ or (((mental* or psycholog*) adj3 (health or care or healthcare or well-being or wellbeing or disease* or disorder*)) or ((behav* or anxiety or depressive) adj3 (disease* or disorder*)) or (eating adj3 disorder*) or depression* or psychosis or psychiatr* or psychoeducat* or psycho-educat* or psychsomatic* or autism* or adhd or (attention adj3 deficit*) or pdd-nos or Asperger* or suicid* or automutilat* or self-harm* or phobia or (fear adj3 (childbirth* or birth*)) or tocophobi* or tokophobi*).ab,ti.) and (exp Continuing Education/ or exp Curriculum/ or exp Program Evaluation/ or Inservice Training/ or Interprofessional Education/ or exp Professional Competence/ or (education* or training or curricul* or skill* or competenc* or student* or (program* adj6 (evaluat* or develop* or implement*))).ab,ti.) and english.la.NOT (news OR congres* OR abstract* OR book* OR chapter* OR dissertation abstract*).pt.
