# Supplementary material for: A systematic review of midwives’ training needs in perinatal mental health and related interventions
Source: Front Psychiatry. 2024 Apr 22;15:1345738. doi: 10.3389/fpsyt.2024.1345738 (PMC11071341; doi:10.3389/fpsyt.2024.1345738)
Supplement: Supplementary Table 2 — List of excluded studies. [file Table_2.docx]

**Supplementary Table 2. List of excluded studies**

Adjorlolo S, Aziato L, Akorli VV. Promoting maternal mental health in Ghana: An examination of the involvement and professional development needs of nurses and midwives. Nurse Educ Pract. 2019;39:105-110. doi: 10.1016/j.nepr.2019.08.008. Epub 2019 Aug 20. PMID: 31446219.

Al-Abri K, Armitage CJ, Edge D. Views of healthcare professionals and service users regarding anti-, peri- and post-natal depression in Oman. J Psychiatr Ment Health Nurs. 2023 Aug;30(4):795-812. doi: 10.1111/jpm.12908. Epub 2023 Feb 13. PMID: 36719270.

Allen R, Festle L. Education to Identify and Reduce Nurse Stigma and Bias Toward Pregnant Women With Substance Use Disorder. *Journal of Obstetric, Gynecologic & Neonatal Nursing*, 2022;*51*(4), S49.

Anderson BL, Dang EP, Floyd RL, Sokol R, Mahoney J, Schulkin J. Knowledge, opinions, and practice patterns of obstetrician-gynecologists regarding their patients' use of alcohol. J Addict Med. 2010;4(2):114-21. doi: 10.1097/ADM.0b013e3181b95015. PMID: 21769028.

Anonymous. Animation to help support women with eating disorders. Community Pract 2018; 91(3): 10-10.

Anonymous. Opioid Use Disorder and Opioid Maintenance Therapy: Considerations for Perinatal Care and Breastfeeding (2019/068). J Midwifery Womens Health 2019. 64(5): 681-682.

Balakrishna R, Teixeira M. Early intervention for increased antenatal anxiety associated with foetal development risk. Community Pract. 2015;88(4):42-6. PMID: 26601435.

Balakrishna R, Teixeira M. Early intervention for increased antenatal anxiety associated with foetal development risk. Community Pract. 2015 Apr;88(4):42-6. PMID: 26601435.

Baston H. Midwifery basics. Antenatal care-monitoring maternal wellbeing. Pract Midwife. 2003;6(3):32-5. PMID: 12677841.

Bilszta J, Ericksen J, Buist A, Milgrom J. A qualitative study of health professionals involved in the care and treatment of women with postnatal emotional distress. *International Journal of Mental Health Promotion*, 2010;*12*(3), 5-13.

Bright D, Gray BJ, Kyle RG, Bolton S, Davies AR. Factors influencing initiation of health behaviour conversations with patients: Cross-sectional study of nurses, midwives, and healthcare support workers in Wales. J Adv Nurs. 2021 Nov;77(11):4427-4438. doi: 10.1111/jan.14926. Epub 2021 Jul 7. PMID: 34235759; PMCID: PMC8518752.

Brugha TS, Smith J, Austin J, Bankart J, Patterson M, Lovett C, Morgan Z, Morrell CJ, Slade P. Can community midwives prevent antenatal depression? An external pilot study to test the feasibility of a cluster randomized controlled universal prevention trial. Psychol Med. 2016 Jan;46(2):345-56. doi: 10.1017/S003329171500183X. Epub 2015 Oct 20. PMID: 26482473; PMCID: PMC4682479.

Byatt N, Biebel K, Lundquist RS, Moore Simas TA, Debordes-Jackson G, Allison J, Ziedonis D. Patient, provider, and system-level barriers and facilitators to addressing perinatal depression. *Journal of Reproductive and Infant Psychology*, 2012;*30*(5), 436-449.

Byatt N, Biebel K, Moore Simas TA, Sarvet B, Ravech M, Allison J, Straus J. Improving perinatal depression care: the Massachusetts Child Psychiatry Access Project for Moms. Gen Hosp Psychiatry. 2016 May-Jun;40:12-7. doi: 10.1016/j.genhosppsych.2016.03.002. Epub 2016 Mar 21. PMID: 27079616.

Byatt N, Brenckle L, Sankaran P, Biebel K , Weinreb L , Allison J, Moore Simas TA. Improving perinatal depression care in obstetric settings: PRogram in Support of Moms (PRISM). Archives of Women's Mental Health (2019) 22:641–707

Byatt N, Masters GA, Twyman J, Hunt A, Hamad C, Maslin M, Moore Simas TA. Building Obstetric Provider Capacity to Address Perinatal Depression Through Online Training. J Womens Health (Larchmt). 2021 Oct;30(10):1386-1394. doi: 10.1089/jwh.2020.8843. Epub 2021 Apr 9. PMID: 33835884; PMCID: PMC8590156.

Carroll JC, Reid AJ, Biringer A, Midmer D, Glazier RH, Wilson L, Permaul JA, Pugh P, Chalmers B, Seddon F, Stewart DE. Effectiveness of the Antenatal Psychosocial Health Assessment (ALPHA) form in detecting psychosocial concerns: a randomized controlled trial. CMAJ. 2005;173(3):253-9. doi: 10.1503/cmaj.1040610. Erratum in: CMAJ. 2005;173(4):345. PMID: 16076821; PMCID: PMC1180654.

Castell E, Stenfert Kroese B. Midwives׳ experiences of caring for women with learning disabilities - A qualitative study. Midwifery. 2016;36:35-42. doi: 10.1016/j.midw.2016.02.001. Epub 2016 Feb 8. PMID: 27106942.

Chrzan-Dętkoś M, Walczak-Kozłowska TZ. How do new mothers perceive screening for perinatal depression?. *Health Psychology Report*, 2021;*9*(3), 207-216.

Cox JL Clinical and research aspects of post-natal depression. Journal of Psychosomatic Obstetrics & Gynecology. 1983; 2:1, 46-53, DOI: 10.3109/01674828309081255

Crepinsek M, Graham I, Van Vorst S. Perinatal mental health and midwifery education. *Australian Midwifery News*, 2018.*18*(2), 32-34.

Cross-Sudworth F, Williams M, Gardosi J. Community midwifery care and social care pathways. *Evidence Based Midwifery* 2015;13(1): 15-21

DiFrisco ES, Amesse L. Maternal Mental Health Assessment During the Prenatal Period, After Birth, and Beyond. *Journal of Obstetric, Gynecologic & Neonatal Nursing*, 2018;*47*(3), S8-S9.

Downes C, Carroll M, Gill A, Monahan M, Higgins A. Practice nurses' knowledge and competence in relation to perinatal mental health care. Practice Nursing 2017; 28(12), 542-551.

Eden C. Midwives' knowledge and management of postnatal depression. Aust J Adv Nurs 1989;7(1): 35-42.

Elshatarat RA, Yacoub MI, Saleh ZT, Ebeid IA, Abu Raddaha AH, Al-Za'areer MS, Maabreh RS. Perinatal Nurses' and Midwives' Knowledge About Assessment and Management of Postpartum Depression. J Psychosoc Nurs Ment Health Serv. 2018;56(12):36-46. doi: 10.3928/02793695-20180612-02. Epub 2018 Jun 20. PMID: 29916523.

Evans K, Moya H, Lambert M, Spiby H. Developing a training programme for midwives and maternity support workers facilitating a novel intervention to support women with anxiety in pregnancy. BMC Pregnancy Childbirth. 2022 Aug 25;22(1):662. doi: 10.1186/s12884-022-04996-2. PMID: 36008799; PMCID: PMC9403963.

Fox D, Solanki MK, Brown MG, Catling C, Scarf V, Sheehy A, ... Baird K. Perinatal mental health: using interactive videos and simulation to develop skills in midwifery practice. *Women and Birth*, 2022;*35*, 57.

Fraser DM. Pre-registration midwifery programmes: a case study evaluation of the non-midwifery placements. Midwifery. 1996;12(1):16-22. doi: 10.1016/s0266-6138(96)90034-0. PMID: 8715932.

Gamble J, Toohill J, Slavin V, Creedy DK, Fenwick J. Identifying Barriers and Enablers as a First Step in the Implementation of a Midwife-Led Psychoeducation Counseling Framework for Women Fearful of Birth. *International Journal of Childbirth*, 2017;*7*(3), 152-168.

Gibson T. Pre-registration midwifery training: including learning disabilities. *British Journal of Midwifery*, 2007;*15*(10), 626-630.

Gillis BD, Holley SL, Leming-Lee TS, Parish AL. Implementation of a Perinatal Depression Care Bundle in a Nurse-Managed Midwifery Practice. Nurs Womens Health. 2019;23(4):288-298. doi: 10.1016/j.nwh.2019.05.007. Epub 2019 Jul 1. PMID: 31271731.

Goldin Evans M, Phillippi S, Gee RE. Examining the Screening Practices of Physicians for Postpartum Depression: Implications for Improving Health Outcomes. Womens Health Issues. 2015;25(6):703-10. doi: 10.1016/j.whi.2015.07.003. Epub 2015 Sep 2. PMID: 26341568.

Gupta J, Kaushal S, Priya T. Knowledge, attitude, and practices of healthcare providers about perinatal depression in Himachal Pradesh-A cross-sectional study. J Family Med Prim Care. 2023 Mar;12(3):478-483. doi: 10.4103/jfmpc.jfmpc_1170_22. Epub 2023 Mar 17. PMID: 37122653; PMCID: PMC10131966.

Hage B, Watson E, Shenai N, Osborne L, Hutner L, Waltner-Toews R, Gopalan P. A Peer-to-Peer, Longitudinal Reproductive Psychiatry Educational Curriculum for Obstetrics/Gynecology Residents. Acad Psychiatry. 2023 Feb;47(1):43-47. doi: 10.1007/s40596-022-01710-4. Epub 2022 Sep 20. PMID: 36127485.

Handmaker NS, Hester RK, Delaney HD. Videotaped training in alcohol counseling for obstetric care practitioners: a randomized controlled trial. Obstet Gynecol. 1999;93(2):213-8. doi: 10.1016/s0029-7844(98)00377-9. PMID: 9932558.

Hawthorne A, Fagan R, Leaver E, Baxter J, Logan P, Snowden A. Undergraduate nursing and midwifery student's attitudes to mental illness. Nurs Open. 2020;7(4):1118-1128. doi: 10.1002/nop2.494. PMID: 32587731; PMCID: PMC7308689.

Herzig K, Huynh D, Gilbert P, Danley DW, Jackson R, Gerbert B. Comparing prenatal providers' approaches to four different risks: alcohol, tobacco, drugs, and domestic violence. Women Health. 2006;43(3):83-101. doi: 10.1300/J013v43n03_05. PMID: 17194679.

Holland CL, Nkumsah MA, Morrison P, Tarr JA, Rubio D, Rodriguez KL, Kraemer KL, Day N, Arnold RM, Chang JC. "Anything above marijuana takes priority": Obstetric providers' attitudes and counseling strategies regarding perinatal marijuana use. Patient Educ Couns. 2016;99(9):1446-51. doi: 10.1016/j.pec.2016.06.003. Epub 2016 Jun 4. PMID: 27316326; PMCID: PMC5007170.

Holland CL, Rubio D, Rodriguez KL, Kraemer KL, Day N, Arnold RM, Tarr JA, Chang JC. Obstetric Health Care Providers' Counseling Responses to Pregnant Patient Disclosures of Marijuana Use. Obstet Gynecol. 2016;127(4):681-687. doi: 10.1097/AOG.0000000000001343. PMID: 26959210; PMCID: PMC4805441.

Horan H, Mobley E, Lavender C, Thompson A, Bryant W, McDaniel J, Robertson E, McIntosh S, Albright DL. "I am busy enough…": Navigating challenges experienced by Medicaid providers serving pregnant people living with substance use disorders in Alabama. J Nurs Scholarsh. 2023 May;55(3):556-565. doi: 10.1111/jnu.12867. Epub 2023 Jan 15. PMID: 36642921.

Howlett H, Mackenzie S, Strehle EM, Rankin J, Gray WK. A Survey of Health Care Professionals' Knowledge and Experience of Foetal Alcohol Spectrum Disorder and Alcohol Use in Pregnancy. Clin Med Insights Reprod Health. 2019;13:1179558119838872. doi: 10.1177/1179558119838872. PMID: 30944523; PMCID: PMC6437318.

Hunt J. Implementing a PMAD Screening Program for Antepartum Inpatients. *Journal of Obstetric, Gynecologic & Neonatal Nursing*, 2023;*52*(4), S44.

Ibrahim FA, Nirisha L, Barikar M, Kumar CN, Chand PK, Manjunatha N, Math SB, Thirthalli J, Manjappa AA, Parthasarathy R, Reddy S, Arora S. Identification of Psychiatric Disorders by Rural Grass-Root Health Workers: Case Series & Implications for the National Mental Health Program of India. Psychiatr Q. 2021;92(1):389-395. doi: 10.1007/s11126-020-09807-5. PMID: 32772306.

Jones CJ, Creedy DK, Gamble JA. Response to: "Detection and management of perinatal depression by midwives". Women Birth. 2013;26(1):e66. doi: 10.1016/j.wombi.2012.07.003. Epub 2012 Aug 17. PMID: 22902208.

Katz CL, Washington FB, Sacco M, Schuetz-Mueller J. A Resident-Based Telepsychiatry Supervision Pilot Program in Liberia. Psychiatr Serv. 2019;70(3):243-246. doi: 10.1176/appi.ps.201800363. Epub 2018 Nov 30. PMID: 30497326.

Keedle H, Stulz V, Conti J, Bentley R, Meade T, Qummouh R, Hay P, Kaye-Smith H, Everitt L, Schmied V. Psychosocial interprofessional perinatal education: Design and evaluation of an interprofessional learning experience to improve students' collaboration skills in perinatal mental health. Women Birth. 2023 Jul;36(4):e379-e387. doi: 10.1016/j.wombi.2023.01.001. Epub 2023 Jan 23. PMID: 36697285.

Kelly J, Birks M. ‘It's the simple things you do first that start the process of help’: Undergraduate nursing and midwifery students’ experiences of the Mental Health First Aid course. *Collegian*, 2017;*24*(3), 275-280.

King L, Pestell S, Farrar S, North N, Brunt C. Screening for antenatal psychological distress. British Journal of Midwifery 2012;20(6), 396-401.

Kohlhoff J, Cibralic S, Tooke S, Hickinbotham R, Knox C, Roach V, Barnett B. Health professional perspectives on an antenatal mental health screening program in a private hospital. Aust N Z J Obstet Gynaecol. 2021;61(6):891-897. doi: 10.1111/ajo.13394. Epub 2021 Jun 13. PMID: 34121178.

Kurtçy A, Gölbaşi Z. Postpartum depression: Knowledge and opinions of nurses and midwives employed in primary health care centers. F.Ü.Sağ.Bil.Tıp Derg. 2014; 28 (3): 93 - 99

Lau R, McCauley K, Moss C, Miles M, Cross W. Evaluation of an advanced perinatal mental health program for midwives. Aust Nurs Midwifery J. 2015;22(11):44. PMID: 26449087.

Lees S, Brown M, Mills N, McCalmont C. Professionals' knowledge of perinatal mental health care: Susan Lees and colleagues report the findings of a survey to evaluate practitioners' knowledge of women's needs and available services, and make recommendations on improving referral pathways and staff training. *Mental Health Practice*, 2009;*13*(4), 24-28.

Lefever-Rhizal D, Collins-Fulea C, Bailey JM. Trauma-Informed Psychosocial Screening and Care Planning: A Patient-Centered Improvement Project in a Midwife Clinic. J Midwifery Womens Health. 2023 Jun 7. doi: 10.1111/jmwh.13512. Epub ahead of print. PMID: 37283369.

Leiferman JA, Dauber SE, Heisler K, Paulson JF. Primary care physicians' beliefs and practices toward maternal depression. J Womens Health (Larchmt). 2008;17(7):1143-50. doi: 10.1089/jwh.2007.0543. PMID: 18657043.

Leiferman JA, Dauber SE, Scott K, Heisler K, Paulson JF. Predictors of Maternal Depression Management among Primary Care Physicians. Depress Res Treat. 2010;2010:671279. doi: 10.1155/2010/671279. Epub 2010 Mar 25. PMID: 21152221; PMCID: PMC2991642.

Lelong N, Kaminski M, Chwalow J, Bean K, Subtil D. Attitudes and behavior of pregnant women and health professionals towards alcohol and tobacco consumption. Patient Educ Couns. 1995;25(1):39-49. doi: 10.1016/0738-3991(94)00695-i. PMID: 7603932.

Lepper HS, DiMatteo MR, Tinsley BJ. Postpartum depression: how much do obstetric nurses and obstetricians know? Birth. 1994 Sep;21(3):149-54. doi: 10.1111/j.1523-536x.1994.tb00514.x. PMID: 7857457.

Logsdon MC, Foltz MP, Scheetz J, Myers JA. Self-efficacy and postpartum depression teaching behaviors of hospital-based perinatal nurses. J Perinat Educ. 2010 Fall;19(4):10-6. doi: 10.1624/105812410X530884. PMID: 21886417; PMCID: PMC2981184.

Lucas VA, Fuentes AB, Lodise J, Gualtieri AW. Postpartum Depression: A Multidisciplinary Initiative for Staff Education and Patient Management. *Journal of Obstetric, Gynecologic & Neonatal Nursing* 2012;*41*, S8.

Lyberg A, Severinsson E. Midwives' supervisory styles and leadership role as experienced by Norwegian mothers in the context of a fear of childbirth. J Nurs Manag. 2010 May;18(4):391-9. doi: 10.1111/j.1365-2834.2010.01083.x. PMID: 20609043.

Mahato PK, van Teijlingen E, Simkhada P, Angell C, Ireland J; THET team. Qualitative evaluation of mental health training of auxiliary nurse midwives in rural Nepal. Nurse Educ Today. 2018;66:44-50. doi: 10.1016/j.nedt.2018.03.025. Epub 2018 Apr 2. PMID: 29665504.

Marnes J, Hall P. Midwifery care: a perinatal mental health case scenario. Women Birth. 2013;26(4):e112-6. doi: 10.1016/j.wombi.2013.07.002. Epub 2013 Sep 27. PMID: 24080179.

Martin-Key NA, Spadaro B, Schei TS, Bahn S. Proof-of-Concept Support for the Development and Implementation of a Digital Assessment for Perinatal Mental Health: Mixed Methods Study. J Med Internet Res. 2021;23(6):e27132. doi: 10.2196/27132. PMID: 34033582; PMCID: PMC8183599.

Masters GA, Xu L, Cooper KM, Moore Simas TA, Brenckle L, Mackie TI, Schaefer AJ, Straus J, Byatt N. Perspectives on addressing bipolar disorder in the obstetric setting. Gen Hosp Psychiatry. 2022 Jul-Aug;77:130-140. doi: 10.1016/j.genhosppsych.2022.05.009. Epub 2022 May 25. PMID: 35640435.

Mathibe-Neke J M, Makobe KE. Drawing a Line between Hope and Accountability: Midwives’ Response to a “Psychosocial Antenatal Care” Pilot Project in Gauteng, South Africa. *Africa Journal of Nursing and Midwifery*, 2020;*22*(2), 24-pages.

Mauthner NS. Postnatal depression: how can midwives help? Midwifery. 1997 Dec;13(4):163-71. doi: 10.1016/s0266-6138(97)80002-2. PMID: 9511683.

McCauley K, Miles M, Moss C, Cross W, Lau R, Barnfield J, Newman L. Perinatal mental health education for midwives in Victoria. *Australian Nursing and Midwifery Journal*, 2014;*21*(8), 47.

McCauley M, Brown A, Ofosu B, van den Broek N. "I just wish it becomes part of routine care": healthcare providers' knowledge, attitudes and perceptions of screening for maternal mental health during and after pregnancy: a qualitative study. BMC Psychiatry. 2019;19(1):279. doi: 10.1186/s12888-019-2261-x. Erratum in: BMC Psychiatry. 2019 Oct 18;19(1):304. PMID: 31500606; PMCID: PMC6734443.

McKay K. An exploration of student midwives perceptions of health promotion in contemporary practice. *MIDIRS Midwifery Digest* 2008; *18*(2), 165-174.

McLachlan HL, Forster DA, Collins R, Gunn J, Hegarty K. Identifying and supporting women with psychosocial issues during the postnatal period: evaluating an educational intervention for midwives using a before-and-after survey. Midwifery. 2011;27(5):723-30. doi: 10.1016/j.midw.2010.01.008. Epub 2010 Oct 2. PMID: 20888094.

Meira BDM, Pereira PADS, Silveira MDFA, Gualda DMR, Santos Jr HPO. Challenges for primary healthcare professionals in caring for women with postpartum depression. *Texto & Contexto-Enfermagem*, 2015;*24*, 706-712.

Miles S. Winning the battle: A review of postnatal depression. *British Journal of Midwifery* 2011;*19*(4), 221-227.

Miller LS et al. Improving the identification and treatment of postpartum depression in a managed care organization. J Clin Outcomes Manage. 2004; 11(3): 157-161.

Mitchell JM, Keenan O, Fakhoury A, Fitzgerald D, Mohamad MM, Imcha M. Is perinatal substance abuse falling through the cracks? Ir J Psychol Med. 2023 May 25:1-4. doi: 10.1017/ipm.2023.22. Epub ahead of print. PMID: 37226938.

Mitchell-Foster SM, Emon CE, Brouwer M, Duncan Elder L, King J. Disconnected perspectives: Patient and care provider's experiences of substance use in pregnancy. Int J Gynaecol Obstet. 2021 Nov;155(2):170-178. doi: 10.1002/ijgo.13919. Epub 2021 Sep 24. PMID: 34496061; PMCID: PMC9293468.

Mivsek AP, Hundley V, Kiger A. Slovenian midwives' and nurses' views on post-natal depression: an exploratory study. Int Nurs Rev. 2008;55(3):320-6. doi: 10.1111/j.1466-7657.2008.00620.x. PMID: 19522949.

Morrello R, Cook PA, Coffey M. "Now, with a bit more knowledge, I understand why I'm asking those questions." midwives' perspectives on their role in the Greater Manchester health and social care partnership's programme to reduce alcohol exposed pregnancies. Midwifery. 2022 Jul;110:103335. doi: 10.1016/j.midw.2022.103335. Epub 2022 Apr 4. PMID: 35427883.

Moy M, Bayliss J, Firth C, Leggate J, Wood R. Drug using parents: an exploration of family focused support from health professionals. *Journal of Research in Nursing*, 2007;*12*(5), 551-561.

Mukherjee R, Wray E, Curfs L, Hollins S. Knowledge and opinions of professional groups concerning FASD in the UK. *Adoption & Fostering*, 2015;*39*(3), 212-224.

Mule V, Reilly NM, Schmied V, Kingston D, Austin MPV. Why do some pregnant women not fully disclose at comprehensive psychosocial assessment with their midwife?. *Women and Birth*. 2021

Munoz K, Suchy C, Rutledge DN. Knowledge and Attitudes of Maternity Nurses and Ancillary Team Members about Substance Addiction during Pregnancy and Postpartum. MCN Am J Matern Child Nurs. 2021;46(2):82-87. doi: 10.1097/NMC.0000000000000703. PMID: 33630491.

Nithianandan N, Gibson-Helm M, McBride J, Binny A, Gray KM, East C, Boyle JA. Factors affecting implementation of perinatal mental health screening in women of refugee background. Implement Sci. 2016 Nov 18;11(1):150. doi: 10.1186/s13012-016-0515-2. PMID: 27863498; PMCID: PMC5116191.

O’Leary K, Bromley E. Developing a mental health pathway for midwifery students. *mental health practice*, 2009;*12*(6).

Ordan R, Shor R, Liebergall-Wischnitzer M, Noble L, Noble A. Nurses' professional stigma and attitudes towards postpartum women with severe mental illness. J Clin Nurs. 2018;27(7-8):1543-1551. doi: 10.1111/jocn.14179. Epub 2018 Jan 11. PMID: 29148602.

Ordean A, Forte M, Selby P, Grennell E. Screening, Brief Intervention, and Referral to Treatment for Prenatal Alcohol Use and Cigarette Smoking: A Survey of Academic and Community Health Care Providers. J Addict Med. 2020;14(4):e76-e82. doi: 10.1097/ADM.0000000000000588. PMID: 31703018; PMCID: PMC7413672.

Oser C, Biebel E, Harris M, Klein E, Leukefeld C. Gender differences in provider's use of a standardized screening tool for prenatal substance use. J Addict Med. 2011;5(1):36-42. doi: 10.1097/ADM.0b013e3181ccec2e. PMID: 21359106; PMCID: PMC3045208.

Palladino CL, Fedock GL, Forman JH, Davis MM, Henshaw E, Flynn HA. OB CARES--The Obstetric Clinics and Resources Study: providers' perceptions of addressing perinatal depression--a qualitative study. Gen Hosp Psychiatry. 2011 May-Jun;33(3):267-78. doi: 10.1016/j.genhosppsych.2011.02.001. Epub 2011 Mar 31. PMID: 21601724.

Paluzzi P, Deggins N, Hutchins E, Burkhardt P. The role of midwives in caring for women with substance use disorders: implications for training. Subst Abus. 2002;23(3 Suppl):223-33. doi: 10.1080/08897070209511517. PMID: 23580997.

Patabendige M, Athulathmudali SR, Chandrasinghe SK. Mental Health Problems during Pregnancy and the Postpartum Period: A Multicenter Knowledge Assessment Survey among Healthcare Providers. J Pregnancy. 2020;2020:4926702. doi: 10.1155/2020/4926702. PMID: 32685213; PMCID: PMC7341390.

Payne JM, Watkins RE, Jones HM, Reibel T, Mutch R, Wilkins A, Whitlock J, Bower C. Midwives' knowledge, attitudes and practice about alcohol exposure and the risk of fetal alcohol spectrum disorder. BMC Pregnancy Childbirth. 2014;14:377. doi: 10.1186/s12884-014-0377-z. PMID: 25366388; PMCID: PMC4228156.

Phoosuwan N, Lundberg PC. Knowledge, attitude and self-efficacy program intended to improve public health professionals' ability to identify and manage perinatal depressive symptoms: a quasi-experimental study. BMC Public Health. 2020 Dec 30;20(1):1926. doi: 10.1186/s12889-020-10086-9. PMID: 33380321; PMCID: PMC7774237.

Pinar S, Ersser SJ, Mcmillan D, Bedford H. Support and services for perinatal low mood and depression: A qualitative study exploring women's and healthcare professionals' experiences. Nurs Health Sci. 2022 Dec;24(4):862-870. doi: 10.1111/nhs.12987. Epub 2022 Oct 4. PMID: 36134463.

Place JM, Billings DL, Blake CE, Frongillo EA, Mann JR, deCastro F. Conceptualizations of postpartum depression by public-sector health care providers in Mexico. Qual Health Res. 2015 Apr;25(4):551-68. doi: 10.1177/1049732314552812. Epub 2014 Oct 3. PMID: 25281238.

Place JMS, Allen-Leigh B, Billings DL, Dues KM, de Castro F. Detection and care practices for postpartum depressive symptoms in public-sector obstetric units in Mexico: Qualitative results from a resource-constrained setting. Birth. 2017;44(4):390-396. doi: 10.1111/birt.12304. Epub 2017 Aug 22. PMID: 28833511.

Pope J, Redsell S, Houghton C, Matvienko-Sikar K. Healthcare professionals' experiences and perceptions of providing support for mental health during the period from pregnancy to two years postpartum. Midwifery. 2023 Mar;118:103581. doi: 10.1016/j.midw.2022.103581. Epub 2022 Dec 24. PMID: 36608486.

Psaros C, Geller PA, Sciscione AC, Bonacquisti A. Screening practices for postpartum depression among various health care providers. J Reprod Med. 2010;55(11-12):477-84. PMID: 21291033.

Radcliffe P. Substance-misusing women: Stigma in the maternity setting. *British journal of midwifery*, 2011;*19*(8), 497-506.

Raeside L. Attitudes of staff towards mothers affected by substance abuse. Br J Nurs. 2003;12(5):302-10. doi: 10.12968/bjon.2003.12.5.11176. PMID: 12682598.

Ramirez-Cacho WA, Strickland L, Beraun C, Meng C, Rayburn WF. Medical students' attitudes toward pregnant women with substance use disorders. Am J Obstet Gynecol. 2007;196(1):86.e1-5. doi: 10.1016/j.ajog.2006.06.092. PMID: 17240247.

Reed M, Fenwick J, Hauck Y, Gamble J, Creedy DK. Australian midwives' experience of delivering a counselling intervention for women reporting a traumatic birth. Midwifery. 2014;30(2):269-75. doi: 10.1016/j.midw.2013.07.009. Epub 2013 Jul 25. PMID: 23972795.

Reilly N, Brake E, Kalra H, Austin MP. Insights into implementation of routine depression screening and psychosocial assessment in a private hospital setting: A qualitative study. Aust N Z J Obstet Gynaecol. 2020;60(3):419-424. doi: 10.1111/ajo.13083. Epub 2019 Oct 24. PMID: 31650543.

Rohanachandra YM, Prathapan S, Amarabandu HGI. The knowledge of Public Health Midwives on Autism Spectrum Disorder in two selected districts of the Western Province of Sri Lanka. Asian J Psychiatr. 2020;52:102094. doi: 10.1016/j.ajp.2020.102094. Epub 2020 Apr 18. PMID: 32334398.

Rollans M, Schmied V, Kemp L, Meade T. 'We just ask some questions…' the process of antenatal psychosocial assessment by midwives. Midwifery. 2013 Aug;29(8):935-42. doi: 10.1016/j.midw.2012.11.013. Epub 2013 Feb 15. PMID: 23415365.

Rompala KS, Cirino N, Rosenberg KD, Fu R, Lambert WE. Prenatal Depression Screening by Certified Nurse-Midwives, Oregon. J Midwifery Womens Health. 2016 Sep;61(5):599-605. doi: 10.1111/jmwh.12491. Epub 2016 Aug 19. PMID: 27541435.

Ross-Davie M, Elliott S, Green L. Planning and implementing mental health training. British Journal of Midwifery, 2007;15(4), 199-203.

Rothera, I., & Oates, M. (2008). Managing perinatal mental health disorders effectively: identifying the necessary components of service provision and delivery. *Psychiatric Bulletin*, *32*(4), 131-133.

Rowan C, Bick D. Revising care to reflect CEMACH recommendations: issues for midwives and the maternity services. *Evidence-Based Midwifery* 2007;*5*(3), 80-87.

Safinya-Davies YZ. Managing depression in obstetrics/gynecology practices: A psychology toolkit. Dissertation Abstracts International: Section B: The Sciences and Engineering 2013;73(11-B(E): No Pagination Specified.

Saleh ZT, Elshatarat RA, Ebeid IA, Aljohani MS, Al-Za'areer MS, Alhujaili AD, Al Tarawneh NS, Abu Raddaha AH. Caring for Women With Postpartum Depression in Saudi Arabia: Nurses' and Midwives' Opinions About Their Roles. J Psychosoc Nurs Ment Health Serv. 2020;58(7):42-51. doi: 10.3928/02793695-20200506-05. Epub 2020 May 12. PMID: 32396208.

Santos Junior HP, Rosa Gualda DM, de Fátima Araújo Silveira M, Hall WA. Postpartum depression: the (in) experience of Brazilian primary healthcare professionals. J Adv Nurs. 2013 Jun;69(6):1248-58. doi: 10.1111/j.1365-2648.2012.06112.x. Epub 2012 Aug 8. PMID: 22882356.

Schaar GL. Postpartum Depression: A Community-Based Education and Screening Program. *Journal of Obstetric, Gynecologic & Neonatal Nursing* 2010;*39*, S37-S38.

Schölin L, Watson J, Dyson J, Smith LA. Midwives' views on alcohol guidelines: A qualitative study of barriers and facilitators to implementation in UK antenatal care. Sex Reprod Healthc. 2021;29:100628. doi: 10.1016/j.srhc.2021.100628. Epub 2021 Apr 28. PMID: 33946025.

Shao WA, Williams JW Jr, Lee S, Badgett RG, Aaronson B, Cornell JE. Knowledge and attitudes about depression among non-generalists and generalists. J Fam Pract. 1997;44(2):161-8. PMID: 9040519.

Shaw MR, Lederhos C, Haberman M, Howell D, Fleming S, Roll J. Nurses' Perceptions of Caring for Childbearing Women who Misuse Opioids. MCN Am J Matern Child Nurs. 2016;41(1):37-42. doi: 10.1097/NMC.0000000000000208. PMID: 26658534.

Skocir AP, Hundley V. Are Slovenian midwives and nurses ready to take on a greater role in caring for women with postnatal depression? Midwifery. 2006;22(1):40-55. doi: 10.1016/j.midw.2005.05.001. Epub 2005 Dec 6. PMID: 16337320.

Sleath BL, Thomas N, Jackson E, West SL, Gaynes BN. Physician reported communication about depression and psychosocial issues during postpartum visits. N C Med J. 2007 May-Jun;68(3):151-5. PMID: 17674685.

Smith LA, Dyson J, Watson J, Schölin L. Barriers and enablers of implementation of alcohol guidelines with pregnant women: a cross-sectional survey among UK midwives. BMC Pregnancy Childbirth. 2021 Feb 15;21(1):134. doi: 10.1186/s12884-021-03583-1. PMID: 33588774; PMCID: PMC7885406.

Sofronas M, Feeley N, Zelkowitz P, Sabbagh M. Obstetric and neonatology nurses' attitudes, beliefs, and practices related to the management of symptoms of maternal depression. Issues Ment Health Nurs. 2011;32(12):735-44. doi: 10.3109/01612840.2011.609635. PMID: 22077746.

Stanley N, Borthwick R, Macleod A. Antenatal depression: mothers’ awareness and professional responses. *Primary Health Care Research & Development*, 2006;*7* (3), 257-268.

Stokes ML. Staff Education to Promote Postpartum Depression Screening (Doctoral dissertation, Walden University). 2021

Supina D, Herman BK, Frye CB, Shillington AC. Knowledge of binge eating disorder: a cross-sectional survey of physicians in the United States. Postgrad Med. 2016;128(3):311-6. doi: 10.1080/00325481.2016.1157441. Epub 2016 Mar 9. PMID: 26906042

Taylor P, Zaichkin J, Pilkey D, Leconte J, Johnson BK, Peterson AC. Prenatal screening for substance use and violence: findings from physician focus groups. Matern Child Health J. 2007;11(3):241-7. doi: 10.1007/s10995-006-0169-9. Epub 2006 Dec 5. PMID: 17146726.

Thomason E, Stacks AM, McComish JF. Early intervention and perinatal depression: is there a need for provider training?. *Early Child Development and Care*, 2010;*180*(5), 671-683.

Thome M, Orlygsdottir B, Elvarsson BT. Evaluation of the clinical effect of an on-line course for community nurses on post-partum emotional distress: a community-based longitudinal time-series quasi-experiment. Scand J Caring Sci. 2012;26(3):494-504. doi: 10.1111/j.1471-6712.2011.00954.x. Epub 2011 Nov 28. PMID: 22122594.

Trop J, Gendenjamts B, Bat-Erdene U, Doripurev D, Ganbold S, Bayalag M, Withers M. Postpartum depression in Mongolia: A qualitative exploration of health care providers' perspectives. Midwifery. 2018;65:18-25. doi: 10.1016/j.midw.2018.06.020. Epub 2018 Jun 23. PMID: 30029083.

Tully L, Garcia J, Davidson L, Marchant S. Role of midwives in depression screening. *British Journal of Midwifery*, 2002;*10*(6), 374-378.

van der Wulp NY, Hoving C, de Vries H. A qualitative investigation of alcohol use advice during pregnancy: experiences of Dutch midwives, pregnant women and their partners. Midwifery. 2013;29(11):e89-98. doi: 10.1016/j.midw.2012.11.014. Epub 2013 Feb 20. PMID: 23434309.

Wangberg SC. Norwegian midwives' use of screening for and brief interventions on alcohol use in pregnancy. Sex Reprod Healthc. 2015;6(3):186-90. doi: 10.1016/j.srhc.2015.03.001. Epub 2015 Mar 11. PMID: 26842644.

Wheatley SL, Brugha TS, Shapiro DA, Berryman JC. PATA PATA: midwives ‘experiences of facilitating a psychological intervention to identify and treat mild to moderate antenatal and PND. MIDIRS: Midwifery digest 2003;13(4), 523-30.

Wulcan AC, Nilsson C. Midwives' counselling of women at specialised fear of childbirth clinics: A qualitative study. Sex Reprod Healthc. 2019;19:24-30. doi: 10.1016/j.srhc.2018.12.001. Epub 2018 Dec 3. PMID: 30928131.
