# Supplementary material for: A systematic review of midwives’ training needs in perinatal mental health and related interventions
Source: Front Psychiatry. 2024 Apr 22;15:1345738. doi: 10.3389/fpsyt.2024.1345738 (PMC11071341; doi:10.3389/fpsyt.2024.1345738)
Supplement: Supplementary Table 3 — List of included studies. [file Table_3.docx]

**Supplementary Tabe 3. List of included studies**

Andersen CG, Thomsen LLH, Gram P, Overgaard C. 'It's about developing a trustful relationship': A Realist Evaluation of midwives' relational competencies and confidence in a Danish antenatal psychosocial assessment programme. Midwifery. 2023 Jul;122:103675. doi: 10.1016/j.midw.2023.103675. Epub 2023 Apr 2. PMID: 37043942.

Asare SF, Rodriguez-Muñoz MF. Understanding Healthcare Professionals' Knowledge on Perinatal Depression among Women in a Tertiary Hospital in Ghana: A Qualitative Study. Int J Environ Res Public Health. 2022 Nov 30;19(23):15960. doi: 10.3390/ijerph192315960. PMID: 36498033; PMCID: PMC9740295.

Badiya PK, Siddabattuni S, Dey D, Hiremath AC, Nalam RL, Srinivasan V, Vaitheswaran S, Ganesh A, Yendluri P, Ramamurthy SS. Task-sharing to screen perinatal depression in resource limited setting in India: Comparison of outcomes based on screening by non-expert and expert rater. Asian J Psychiatr. 2021 Aug;62:102738. doi: 10.1016/j.ajp.2021.102738. Epub 2021 Jun 26. PMID: 34216979.

Buist A, Bilszta J, Milgrom J, Barnett B, Hayes B, Austin MP. Health professional's knowledge and awareness of perinatal depression: results of a national survey. Women Birth. 2006 Mar;19(1):11-6. doi: 10.1016/j.wombi.2005.12.001. PMID: 16791999.

Bye A, Shawe J, Bick D, Easter A, Kash-Macdonald M, Micali N. Barriers to identifying eating disorders in pregnancy and in the postnatal period: a qualitative approach. BMC Pregnancy Childbirth. 2018;18(1):114. doi: 10.1186/s12884-018-1745-x. PMID: 29759082; PMCID: PMC5952825.

Carroll M, Downes C, Gill A, Monahan M, Nagle U, Madden D, Higgins A. Knowledge, confidence, skills and practices among midwives in the republic of Ireland in relation to perinatal mental health care: The mind mothers study. Midwifery. 2018;64:29-37. doi: 10.1016/j.midw.2018.05.006. Epub 2018 May 18. PMID: 29864579.

Corse SJ, McHugh MK, Gordon SM. Enhancing provider effectiveness in treating pregnant women with addictions. J Subst Abuse Treat. 1995;12(1):3-12. PMID: 7752295.

Cunningham C, Galloway S. LET'S END THE POSTCODE LOTTERY. *Community Practitioner* 2019;*92*(6), 26-29.

Davies L, Page N, Glover H, Sudbury H. Developing a perinatal mental health module: An integrated care approach. *British Journal of Midwifery* 2016;*24*(2), 118-121.

de Vries NE, Stramrood CAI, Sligter LM, Sluijs AM, van Pampus MG. Midwives' practices and knowledge about fear of childbirth and postpartum posttraumatic stress disorder. Women Birth. 2020 Feb;33(1):e95-e104. doi: 10.1016/j.wombi.2018.11.014. Epub 2018 Dec 19. PMID: 30579925.

Dubreucq M, Jourdan S, Poizat A, Dubreucq J. Ressenti des sages-femmes dans la prise en charge en suites de couche des patientes avec troubles psychiques sévères : une analyse qualitative [Midwives' feelings about the post-partum care of women with severe mental illness: A qualitative analysis]. Encephale. 2020;46(3):226-230. French. doi: 10.1016/j.encep.2019.07.009. Epub 2019 Sep 12. PMID: 31522833.

Edge D. Falling through the net - black and minority ethnic women and perinatal mental healthcare: health professionals' views. Gen Hosp Psychiatry. 2010;32(1):17-25. doi: 10.1016/j.genhosppsych.2009.07.007. Epub 2009 Sep 24. PMID: 20114124.

Elliott S, Ross-Davie M, Sarkar A, Green L. Detection and initial assessment of mental disorder: the midwife's role. *British Journal of Midwifery*, 2007; *15*(12), 759-764.

Fletcher A, Murphy M, Leahy-Warren P. Midwives' experiences of caring for women's emotional and mental well-being during pregnancy. J Clin Nurs. 2021;30(9-10):1403-1416. doi: 10.1111/jocn.15690. Epub 2021 Feb 27. PMID: 33527534.

Fontein-Kuipers YJ, Budé L, Ausems M, de Vries R, Nieuwenhuijze MJ. Dutch midwives' behavioural intentions of antenatal management of maternal distress and factors influencing these intentions: an exploratory survey. Midwifery. 2014;30(2):234-41. doi: 10.1016/j.midw.2013.06.010. Epub 2013 Jul 12. PMID: 23856316.

Forrest E, Poat A. Perinatal mental health education for midwives in Scotland. BR J MIDWIFERY 2010;18(5): 280-284.

Fox D, Solanki K, Brown G, Catling C, Scarf V, Sheehy A, Musgrave L, Margetts J, McEwen T, Abela P, Baird K. Perinatal mental healthcare: Developing skills in midwifery students. Women Birth. 2023 Mar;36(2):167-170. doi: 10.1016/j.wombi.2022.11.005. Epub 2022 Nov 29. PMID: 36460560.

Gibb S, Hundley V. What psychosocial well-being in the postnatal period means to midwives. Midwifery. 2007;23(4):413-24. doi: 10.1016/j.midw.2006.07.005. Epub 2006 Dec 13. PMID: 17169469.

Gunn J, Hegarty K, Nagle C, Forster D, Brown S, Lumley J. Putting woman-centered care into practice: a new (ANEW) approach to psychosocial risk assessment during pregnancy. Birth. 2006 Mar;33(1):46-55. doi: 10.1111/j.0730-7659.2006.00073.x. PMID: 16499531.

Hauck YL, Kelly G, Dragovic M, Butt J, Whittaker P, Badcock JC. Australian midwives knowledge, attitude and perceived learning needs around perinatal mental health. Midwifery. 2015;31(1):247-55. doi: 10.1016/j.midw.2014.09.002. Epub 2014 Sep 16. PMID: 25262025.

Hegarty K, Brown S, Gunn J, Forster D, Nagle C, Grant B, Lumley J. Women's views and outcomes of an educational intervention designed to enhance psychosocial support for women during pregnancy. Birth. 2007;34(2):155-63. doi: 10.1111/j.1523-536X.2007.00163.x. PMID: 17542820.

Higgins A, Carroll M, Sharek D. It opened my mind: student midwives' views of a motherhood and mental health module. *MIDIRS Midwifery Digest* 2012;*22*(3), 287-92.

Higgins A, Carroll M, Sharek D. Impact of perinatal mental health education on student midwives' knowledge, skills and attitudes: A pre/post evaluation of a module of study. Nurse Educ Today. 2016;36:364-9. doi: 10.1016/j.nedt.2015.09.007. Epub 2015 Sep 26. PMID: 26431740.

Higgins A, Downes C, Carroll M, Gill A, Monahan M. There is more to perinatal mental health care than depression: Public health nurses reported engagement and competence in perinatal mental health care. J Clin Nurs. 2018;27(3-4):e476-e487. doi: 10.1111/jocn.13986. Epub 2017 Dec 7. PMID: 28771981.

Higgins A, Downes C, Monahan M, Gill A, Lamb SA, Carroll M. Barriers to midwives and nurses addressing mental health issues with women during the perinatal period: The Mind Mothers study. J Clin Nurs. 2018;27(9-10):1872-1883. doi: 10.1111/jocn.14252. Epub 2018 Mar 13. PMID: 29314366.

Hiremath P, Mohite VR, Salimath G, Wesley DC, Naregal P, Chendake M, ... More UR. A study to assess the effectiveness of the structured teaching program on knowledge of postnatal depression among staff nurses in selected hospital at Tumkur. *Journal of Evolution of Medical and Dental Sciences*, 2016;*5*(62), 4337-4342.

Hooks C. Attitudes toward substance misusing pregnant women following a specialist education programme: An exploratory case study. Midwifery. 2019;76:45-53. doi: 10.1016/j.midw.2019.05.011. Epub 2019 May 27. PMID: 31163295.

Işık SN, Bilgili N. Postnatal depression: Midwives’ and nurses’ knowledge and practices. *Erciyes Medical Journal*, 2010;*32*(4), 265-274.

Jardri R, Maron M, Pelta J, Thomas P, Codaccioni X, Goudemand M, Delion P. Impact of midwives' training on postnatal depression screening in the first week post delivery: a quality improvement report. Midwifery. 2010 Dec;26(6):622-9. doi: 10.1016/j.midw.2008.12.006. Epub 2009 Feb 10. PMID: 19211177.

Jarrett P. Attitudes of student midwives caring for women with perinatal mental health problems. BR J MIDWIFERY 2014;22(10): 718-724.

Jarrett P. Student midwives’ knowledge of perinatal mental health. BR J MIDWIFERY 2015;23(1): 32-39.

Jomeen J, Glover LF, Davies SA. Midwives' illness perceptions of antenatal depression. *British Journal of Midwifery* 2009;*17*(5), 296-303.

Jones CJ, Creedy DK, Gamble JA. Australian midwives' knowledge of antenatal and postpartum depression: a national survey. J Midwifery Womens Health. 2011;56(4):353-361. doi: 10.1111/j.1542-2011.2011.00039.x. PMID: 21733106.

Jones CJ, Creedy DK, Gamble JA. Australian midwives' attitudes towards care for women with emotional distress. Midwifery. 2012;28(2):216-21. doi: 10.1016/j.midw.2010.12.008. Epub 2011 Feb 20. PMID: 21342738.

Jones CJ, Creedy DK, Gamble JA. Australian midwives' awareness and management of antenatal and postpartum depression. Women Birth. 2012 Mar;25(1):23-8. doi: 10.1016/j.wombi.2011.03.001. Epub 2011 Apr 3. PMID: 21459691.

Keng SL. Malaysian midwives' views on postnatal depression. BR J MIDWIFERY 2005;13(2): 78-86.

Larkin V, Flaherty A, Keys C, Yaseen J. Exploring maternal perinatal mental health using a blended learning package. *British Journal of Midwifery* 2014;*22*(3), 210-217.

Lau R, McCauley K, Barnfield J, Moss C, Cross W. Attitudes of midwives and maternal child health nurses towards suicide: A cross-sectional study. Int J Ment Health Nurs. 2015;24(6):561-8. doi: 10.1111/inm.12162. Epub 2015 Sep 8. PMID: 26350295.

Madden D, Sliney A, O'Friel A, McMackin B, O'Callaghan B, Casey K, Courtney L, Fleming V, Brady V. Using action research to develop midwives' skills to support women with perinatal mental health needs. J Clin Nurs. 2018;27(3-4):561-571. doi: 10.1111/jocn.13908. Epub 2017 Oct 4. PMID: 28557236.

Magdalena CD, Tamara WK. Antenatal and postnatal depression - Are Polish midwives really ready for them? Midwifery. 2020;83:102646. doi: 10.1016/j.midw.2020.102646. Epub 2020 Jan 22. PMID: 32004734.

McCann TV, Clark E. Australian Bachelor of Midwifery students' mental health literacy: an exploratory study. Nurs Health Sci. 2010;12(1):14-20. doi: 10.1111/j.1442-2018.2009.00477.x. PMID: 20487320.

McCauley K, Elsom S, Muir-Cochrane E, Lyneham J. Midwives and assessment of perinatal mental health. J Psychiatr Ment Health Nurs. 2011;18(9):786-95. doi: 10.1111/j.1365-2850.2011.01727.x. Epub 2011 May 22. PMID: 21985681.

McGlone C, Hollins Martin CJ, Furber C. Midwives’ experiences of asking the Whooley questions to assess current mental health: a qualitative interpretive study. *Journal of reproductive and infant psychology*, 2016;*34*(4), 383-393.

McGookin A, Furber C, Smith DM. Student midwives' awareness, knowledge, and experiences of antenatal anxiety within clinical practice. J Reprod Infant Psychol. 2017;35(4):380-393. doi: 10.1080/02646838.2017.1337270. Epub 2017 Jun 30. PMID: 29517371.

Noonan M, Jomeen J, Galvin R, Doody O. Survey of midwives' perinatal mental health knowledge, confidence, attitudes and learning needs. Women Birth. 2018;31(6):e358-e366. doi: 10.1016/j.wombi.2018.02.002. Epub 2018 Feb 14. PMID: 29454664.

Noonan M, Galvin R, Jomeen J, Doody O. Public health nurses' perinatal mental health training needs: A cross sectional survey. J Adv Nurs. 2019;75(11):2535-2547. doi: 10.1111/jan.14013. Epub 2019 Aug 27. PMID: 30937923.

Nyberg K, Lindberg I, Öhrling K. Midwives' experience of encountering women with posttraumatic stress symptoms after childbirth. Sex Reprod Healthc. 2010 Apr;1(2):55-60. doi: 10.1016/j.srhc.2010.01.003. Epub 2010 Feb 13. PMID: 21122597.

Oni HT, Buultjens M, Blandthorn J, Davis D, Abdel-Latif M, Islam MM. Barriers and facilitators in antenatal settings to screening and referral of pregnant women who use alcohol or other drugs: A qualitative study of midwives' experience. Midwifery. 2020 Feb;81:102595. doi: 10.1016/j.midw.2019.102595. Epub 2019 Dec 2. PMID: 31838337.

Pearson, P., et al. (2019). Reducing Barriers That Hinder Obstetric Providers From Addressing Perinatal Depression: A Provider Education Module. J Dr Nurs Pract 12(2): 212-224.

Phillips L. Assessing the knowledge of perinatal mental illness among student midwives. Nurse Educ Pract. 2015 Nov;15(6):463-9. doi: 10.1016/j.nepr.2014.09.003. Epub 2014 Sep 28. PMID: 25300675.

Ross-Davie M, Elliott S, Sarkar A, Green L. A public health role in perinatal mental health: are midwives ready?. *British journal of midwifery*, 2006;*14*(6), 330-334.

Rothera I, Oates M. Managing perinatal mental health: A survey of practitioners’ views. *British Journal of Midwifery*, 2011;*19*(5), 304-313.

Salomonsson B, Wijma K, Alehagen S. Swedish midwives' perceptions of fear of childbirth. Midwifery. 2010 Jun;26(3):327-37. doi: 10.1016/j.midw.2008.07.003. Epub 2008 Sep 6. PMID: 18774630.

Salomonsson B, Alehagen S, Wijma K. Swedish midwives' views on severe fear of childbirth. Sex Reprod Healthc. 2011 Nov;2(4):153-9. doi: 10.1016/j.srhc.2011.07.002. Epub 2011 Aug 3. PMID: 22055984.

Sanders LB. Attitudes, perceived ability, and knowledge about depression screening: a survey of certified nurse-midwives/certified midwives. J Midwifery Womens Health. 2006;51(5):340-346. doi: 10.1016/j.jmwh.2006.02.011. PMID: 16945781.

Savory NA, Sanders J, Hannigan B. Midwives' experiences of supporting women's mental health: A mixed-method study. Midwifery. 2022 Aug;111:103368. doi: 10.1016/j.midw.2022.103368. Epub 2022 May 11. PMID: 35617880.

Schouten BC, Westerneng M, Smit AM. Midwives' perceived barriers in communicating about depression with ethnic minority clients. Patient Educ Couns. 2021;104(10):2393-2399. doi: 10.1016/j.pec.2021.07.032. Epub 2021 Jul 24. PMID: 34340845.

Shahid Ali S, Letourneau N, Rajan A, Jaffer S, Adnan F, Asif N, Ali TS. Midwives' perspectives on perinatal mental health: A qualitative exploratory study in a maternity setting in Karachi, Pakistan. Asian J Psychiatr. 2023 Feb;80:103356. doi: 10.1016/j.ajp.2022.103356. Epub 2022 Nov 26. PMID: 36470193.

Shinohara E, Ohashi Y, Hada A, Usui Y. Effects of 1-day e-learning education on perinatal psychological support skills among midwives and perinatal healthcare workers in Japan: a randomised controlled study. BMC Psychol. 2022 May 23;10(1):133. doi: 10.1186/s40359-022-00832-6. PMID: 35606868; PMCID: PMC9125975.

Stewart C, Henshaw C. Midwives and perinatal mental health." BR J MIDWIFERY 2002;10(2): 117-121.

Toler S, Stapleton S, Kertsburg K, Callahan TJ, Hastings-Tolsma M. Screening for postpartum anxiety: A quality improvement project to promote the screening of women suffering in silence. Midwifery. 2018;62:161-170. doi: 10.1016/j.midw.2018.03.016. Epub 2018 Apr 3. PMID: 29684795; PMCID: PMC8040026.

Varotariya JK, Dere SS, Ghildiyal RP. To study knowledge and attitude regarding post-partum depression before and after a structured teaching program intervention among registered staff nurses. *International Journal of Indian Psychȯlogy*, 2019;*7*(4).

Whitehead R, O'Callaghan F, Gamble J, Reid N. Contextual influences experienced by Queensland midwives: a qualitative study focusing on alcohol and other substance use during pregnancy. International Journal of Childbirth 2019;9(2), 80-91.

Wickberg B, Tjus T, Hwang P. Using the EPDS in routine antenatal care in Sweden: a naturalistic study. *Journal of reproductive and infant psychology*, 2005.*23*(1), 33-41.

Willey SM, Gibson-Helm ME, Finch TL, East CE, Khan NN, Boyd LM, Boyle JA. Implementing innovative evidence-based perinatal mental health screening for women of refugee background. Women Birth. 2020;33(3):e245-e255. doi: 10.1016/j.wombi.2019.05.007. Epub 2019 Jun 8. PMID: 31182352.

Williams CJ, Turner KM, Burns A, Evans J, Bennert K. Midwives and women's views on using UK recommended depression case finding questions in antenatal care. Midwifery. 2016 Apr;35:39-46. doi: 10.1016/j.midw.2016.01.015. Epub 2016 Feb 9. PMID: 27060399.

Yamashita H, Ariyoshi A, Uchida H, Tanishima H, Kitamura T, Nakano H. Japanese midwives as psychiatric diagnosticians: application of criteria of DSM-IV mood and anxiety disorders to case vignettes. Psychiatry Clin Neurosci. 2007;61(3):226-33. doi: 10.1111/j.1440-1819.2007.01659.x. PMID: 17472589.
