# Supplementary material for: A systematic review of midwives’ training needs in perinatal mental health and related interventions
Source: Front Psychiatry. 2024 Apr 22;15:1345738. doi: 10.3389/fpsyt.2024.1345738 (PMC11071341; doi:10.3389/fpsyt.2024.1345738)
Supplement: Supplementary Table 4 — Characteristics of included studies about midwives' knowledge, skills and attitude. [file Table_4.docx]

Supplementary Table 4. Characteristics of included studies about midwives' knowledge, skills and attitude

| Source and country | Population (N, specific training about perinatal mental health, specialization) and sampling | Design and type of study | Condition examined and period | Assessment tools or methods (e.g. scales or probes for qualitative studies) | Key finding | Cultural / local specificities | Quality rating (MMAT) |
| --- | --- | --- | --- | --- | --- | --- | --- |
| Andersen et al., 2023  Denmark | N=15 midwives  8 received previous training and 4 worked in vulnerability specialized clinic  Convenience sample | Qualitative study  Multi-site | Perinatal mental health problems | Qualitative realist interviews (aim=identification of contextually grounded explanatory mechanisms)  Theory development trough a literature review  Theory testing / refinement  Online semi-structured interviews combined with 16 observations of 8 midwives conducting a psychosocial assessment  Tape-recording and transcribed verbatim, theory-driven analysis using N-Vivo. Theory refinement during coding. No description of reflexivity.  Data triangulation using field notes | Experience in conducting psychosocial assessment contributes to better verbal and non-verbal relational skills (e.g. telling the parents about the purpose of assessment, symmetry in the relationship, collaborative relationships and person-centered care). Flexible use of psychosocial assessment.  Inexperience in conducting psychosocial assessment results in lack of perceived skills and confidence in asking sensitive questions. Some midwives reported feelings that their personal boundaries were violated. Non-flexible use of psychosocial assessment (feeling compelled to undertake assessment as a standardized survey; institutional demands considered as overwhelming). Distant and superficial manner of asking questions  Personal interest in working with vulnerable populations increases midwives' perceived skills and confidence in undertaking psychosocial assessment and asking sensitive questions (e.g. increased curiosity for meetings the needs of expectant parents and interest to develop their relational skills). Influence on midwives' ability to develop a trustful relationship and to explore opportunities for referral to supportive interventions  Barriers (resulting in discomfort / meaninglessness): lack of formal training on psychosocial assessment (despite regional health policy), lack of clarity about their role (feeling of inadequacy and anxiety), lack of knowledge about how to adequately use the interview and about referral pathways.  Importance of peer support (e.g. informal reflection between peers during lunch breaks) and formal supervision to enhance relational skills and confidence. Need for priorizing peer reflection / supervision in local policies  Continuity of care and flexibility in time use is critical when working with parents in vulnerable positions. Negative impact of workload related pressures on relational skills (e.g. prioritization of other tasks and more distant relationship; feeling compelled to meet the needs of the institution vs. the needs of parents) | NA | (1)  1: Y  2: Y  3: Y  4: N  5: Y  High |
| Asare & Rodiguez-Munoz 2022  Ghana | N=11 health providers (8 midwives, one obstetrician and 2 specialist psychiatrists) working at antenatal and postnatal clinic  Convenience sample | Qualitative study  Single-site | Perinatal depression | Open ended question related to health providers' knowledge about detection and management of perinatal depression (risk factors, symptoms, screening, screening tools, management, treatment options, perceived barriers for help seeking)  Pretest of the interview guide (n=3 midwives)  Face-to-face interviews led by the first author who does not work in the place of the study. Interviews conducted in English (or in English and Twi, the local language; translation from Twi to English and then from English to twi)  Tape-recording and transcribed verbatim. Member checking with 3 participants  Inductive thematic analysis (coding by a single researcher). Use of NVivo 12  Data saturation obtained | Absence of local guidelines on the management of perinatal depression ; lack of clear referral pathways / adequate referral options, long waiting times for an obstetric visit (insufficient number of staff members), lack of confidentiality, workload / time constraints  Negative attitudes towards perinatal parents (e.g. not ready to listen but ready to give her instructions; reactions of being scared / burdened in case of positive answer) ; lack of knowledge / skills, no use of screening tools in the facility  Use of pharmacological options before referral to psychiatry ; over-referral to MH services related to lack of knowledge / skills | Cultural barriers to effective communication with perinatal parents (supernatural causal attributions and negative attitudes towards mental illness and help seeking) | (1)  1: Y  2: Y  3: Y  4: N  5: N  Moderate |
| Buist et al., 2006  Australia | N=569 midwives N=246 general practitioners (GP); N=338 Maternal Child Health Nurses (MCHN)  Convenience sample | Quantitative, descriptive study  Cross-sectional  Multi-site | Perinatal depression | Antenatal and postnatal depression vignettes derived from validated vignettes for depression(questions related to identification, need for support, treatment)  10-item knowledge questionnaire derived from a validated measure | Compared with the other groups, GPs were more likely to correctly identify the vignette (diagnostic accuracy=79%)  Compared with the other groups, midwives were less likely to think that the parent depicted in the vignette needed additional support  GPs were significantly more likely to prescribe antidepressants in the antenatal (compared with midwives and MCHN) and postnatal period (compared with midwives)  Compared with GPs, midwives were more likely to recommend antipsychotics in the postpartum period  No influence of the level of experience / the level of knowledge on case identification and perceived need for support  Limit: low response rate for GPs | Lower scores for GPs in Queensland compared with the other states | (4)  1: Y  2: N  3: N  4: N  5: N  Low |
| Bye et al., 2018  UK | N=33  Qualified providers  n=14 midwives  n=9 health visitors  Students  n=5 student midwives; n=5 student health visitors  71% received training in perinatal mental health  29% received training on eating disorders  2^nd^ part of the study on parents with current or past eating disorder  Convenience sample | Qualitative analysis  Cross-sectional  Multi-site | Eating disorders (ED) during the antenatal and postpartum period (Anorexia Nervosa, Bulimia  Nervosa and Binge-Eating Disorder) | Focus groups  Semi-structured interview addressing knowledge and attitudes towards screening for ED, health providers' role and needs for support  Recording and transcription verbatim  Thematic analysis (independent coding by two researchers, agreement rate=79%) | Lack of training / knowledge on eating disorders (media or personal clinical experience are the main sources of knowledge)  Lack of confidence in enquiring / identifying ED that affected parents' decisions to disclose or not their ED  Lack of knowledge of management plans / available resources / referral pathways  Concerns about sharing sensitive information with colleagues / confidentiality  Fragmented care, infant-centered care, lack of multidisciplinary work and lack of time were barriers to screening for ED.  Stigma-related discomfort when discussing ED with parents ("taboo") | NA | (1)  1: Y  2: Y  3: N  4: N  5: Y  Moderate |
| Carroll et al., 2018  Ireland | N=438 midwives (2 midwives also qualified as mental health nurse)  63.6% received training about perinatal mental health (20% in-service education)  Convenience sample (24.8% of all midwives in Ireland) | Mixed method study  Descriptive study and qualitative content analysis  Cross-sectional  Online survey | Peripartum (pregnancy to the 1^st^ year postpartum)  Perinatal mental health problems (PMHPs): anxiety, depression, bipolar disorders, psychosis, SUD, PTSD, OCD, personality disorders, suicide and self-harm. | Online survey using a self-designed questionnaire that included: contact with parents with PMHPs; 19 items on knowledge (Likert scales ranging from 1 to 5); 35 items on skills, 2 items on overall skill and confidence in PMHC; 23 items on mental health practices; 6 items perinatal mental health services and guidelines  Face validity testing by experts in PMHC  Open-ended questions:  Educational and practice priorities | 90% of the participants cared for parents with PMHPs in their current role  Self-reported knowledge above average for perinatal anxiety or depression  Self-reported knowledge below average for psychosis, bipolar disorder, eating disorder, psychotropic drugs, screening tools, self-injury / suicide, OCD, legal aspects and personality disorders  Self-reported skills above average for opening discussions about mood or anxiety / providing support to parents with childbirth trauma or emotional distress / referring to perinatal mental health services, social workers or GP  Self-reported skills below average for opening discussion about psychosis or sexual violence / developing a care plan for parents with thoughts or harming themselves or their baby or parents hearing voices or having delusions  Midwives considered discussion about perinatal mental health as part of their role (expect for preexisting mental health problems)  Significant influence of perinatal mental health education on knowledge, skills and confidence in PMHC.  Need for training on risk factors, cultural and legal issues, bonding and interviewing skills (opening discussions) | NA | (1)  1: N  2: N  3: N  4: N  5: N  (4)  1: Y  2: N  3: N  4: N  5: Y  (5)  1: Y  2: N  3: N  4: N  5: N  Low |
| Cunningham & Galloway 2019  Northern Ireland | N=332 (130 health visitors and 202 midwives)  80% of them received training in perinatal mental health problems  Convenience sample  (15% of all midwives; 23% of all health visitors) | Quantitative descriptive study  Cross-sectional  Online survey | perinatal mental health problems  (not specified)  Pregnancy to 1^st^ year postpartum | Online survey about views and experiences by identifying and responding to peripartum mental illnesses | Of participants who received training in perinatal mental health problems, 71% said it covered the potential impact on the child.  Compared with health visitors, midwives had lower mean levels of confidence in identifying mental illnesses, in referring parents with perinatal mental health problems to their GP and in managing these parents  Positive impact of the number of years of experience on the level of confidence (none found for training)  2/3 routinely used the EPDS  Enabling factors for identifying perinatal mental health problems: continuity of care, home visits and screening tools  Barriers to disclosure: fear of consequences, fear of labeling and lack of confidence  Challenges to identification: lack of time to provide person-centered care and workload pressures | Northern Ireland has the lower rate of specialist services for perinatal mental health in the UK. | (4)  1: Y  2: N  3: N  4: N  5: N  Low |
| De Vries et al., 2020  The Netherlands | N=257 midwives (full data for 217 midwives)  Community and hospital midwives  Convenience sample (mail sent to all Dutch midwives, 16% response rate) | Quantitative descriptive study  Cross-sectional  Multi-site | Fear of childbirth (FOC) and postpartum posttraumatic stress disorder(PTSD) | 27 item self-designed questionnaire combining multiple choice and open-ended questions. Themes covered: knowledge, organization of care and attitudes  Comparison between hospital and community midwives  Calculation of kappa scores between two research fellows for rating knowledge (> 0.61; not detailed) | No difference between community and hospital midwives in knowledge about FOC or PTSD  Better knowledge about FOC than  postpartum PTSD  No difference between community and hospital midwives in care for parents with FOC  Group differences in care for PTSD: more referral to psychologists or social workers for hospital midwives and more referral to life coaches, midwives with coaching skills or complementary and alternative therapies for community midwives ; more importance given to continuity of care by community midwives  Generally positive emotions and attitudes for both conditions- more negative emotions (guilt, sadness, frustration) related to postpartum PTSD | In the Netherlands,  Healthy parents with low-risk profiles are entirely followed up by community midwives (no supervision by an obstetrician)  If complications arise, parents are referred to hospital midwives (direct or indirect supervision by an obstetrician) | (4)  1: Y  2: N  3: N  4: N  5: Y  Moderate |
| Dubreucq et al., 2019  France | Hospital midwives (n=20)  -One midwife had a family member with SMI  -None of the midwives received specific training  Sampling = all midwives involved in postpartum care of three parents with SMI | Qualitative study  Cross-sectional  Single site | Schizophrenia and bipolar disorder during the postpartum period | Individual interviews (4 open-ended questions related to difficulties, resources, feeling and unmet needs in care of parents with SMI)  Thematic analysis  Independent coding by two researchers  Data saturation obtained | Feeling of discomfort when midwives felt not able to consider the parents as a parent and not only as someone with mental illness  Feeling of discomfort and powerlessness related to lack of knowledge, lack of interviewing skills, lack of training and lack of access to reactive perinatal mental health care  Conversely, midwives reported to feel comfortable if they could provide good quality of care for parents with SMI / if there was access to reactive perinatal mental health care  Importance of training (sources of knowledge = personal clinical experience and academic training) | NA | (1)  1: Y  2: Y  3: Y  4: N  5: N  Moderate |
| Edge, 2010  UK | N=42 healthcare providers (27 midwives; 64.3%)  Nine Black Caribbean parents  Data on 42 Black Caribbean parents with lived experience published in a companion paper (Edge, 2011)  Convenience sample  Purposive recruiting to have various perspectives (midwives, GPs, health visitors, hospital doctors)  This research included parents with lived experience - published in Edge, 2011 | Qualitative study  Cross-sectional  Single-site | Perinatal depression in Black Caribbean parents | Individual interviews and focus groups  Open-ended questions related to healthcare professionals' views about perinatal mental healthcare for Black Caribbean parents  Coding and analysis by one researcher (the 1^st^ author)  Framework analysis method | Missed opportunities in the antenatal care (overshadowing by physical health problems) even for parents with past perinatal depression  Missed opportunities in the postpartum period (negative attitudes towards screening tools and cultural bias affecting the diagnosis rate - "they don't kill themselves")  All providers (in particular midwives and health visitors) reported a lackof knowledge, confidence and competence in identifying and managing perinatal depression - irrespective of ethnic origin. This added to difficulties in referring these parents to perinatal mental health care services  Other barriers: lack of time, lack of clear referral pathways, lack of timely access to specialist services, lack of continuity of care  Completed by Edge, 2011  Negative experiences of maternity care (poor physical care and lack of compassion), poor communication with staff members (disappointment and mistrust) and infant-centered care that were barriers to accessing PMHC | Cultural aspects in Edge, 2010 and Edge, 2011  Negative attitudes towards help-seeking in this population (cultural differences for staff members and consequences of negative experiences of healthcare for parents)  Staff lack of awareness / knowledge of culturally specific issues | (1)  1: Y  2: N  3: Y  4: N  5: N  Moderate |
| Fletcher et al., 2021  Ireland | N=10 midwives (90% public sector)  3 had additional postgraduate training in perinatal mental health  Convenience sample | Qualitative study  Cross-sectional  Single-site | Perinatal mental health problems (i.e. mental health and well-being of parents during pregnancy and up until the first-year post-partum) | Individual interviews  Sample size based on redundancy of interviews rather than data saturation  Open-ended questions (not detailed)  Transcription verbatim and coding by one researcher  Thematic analysis (methods not clearly detailed) | Equal importance of physical and emotional wellbeing - participants considered that was part of their role to identify and refer parents with perinatal mental health problems  Opportunities to discuss emotional wellbeing include the 1^st^ booking appointments and antenatal classes  Negative attitudes towards screening tools (preference for open-ended questions)  Challenges include parents' hesitations / reluctance to discuss emotional and mental health and stigma  Stigma-related negative attitudes towards help seeking in midwives if they were themselves concerned by mental health issues  Importance of interviewing skills  Detection also relies on behavior observation (parent body language, interaction with the baby) and level of social support  Other barriers: lack of time, workload pressures, lack of continuity of care, lack of clear referral pathways  Need for additional training and supervision | NA | (1)  1: N  2: N  3: N  4: N  5: Y  Low |
| Fontein-Kuipers et al., 2014  The Netherlands | N=112 community midwives  Convenience sample | Quantitative descriptive survey  Cross-sectional | Maternal distress (depression, anxiety, stress, worries and fears but not serious mental illness) | Self-designed 50 item questionnaire assessing behavioral intention to screen and support parents with maternal distress and to collaborate with other providers  Internal consistency (CA)= 0.67-0.86 for the 12 subscales  Face validity tested with 14 midwives  No a priori sample size calculation  Multivariable linear regressions for each component (screening, support, collaboration) | Predictors of intention to screen= years of experience, finding maternal distress an interesting topic, attitudes toward screening, and self-efficacy in screening  Predictors of the intention to support =finding maternal distress an interesting topic and attitude toward support.  Predictors of the intention to collaborate with other health-care providers =finding maternal distress an interesting topic and years of work experience | NA | 1: N  2: N  3: Y  4: N  5: Y  Moderate |
| Gibbs and Hundley, 2007  UK (Scotland) | Community midwives (n=13) and student midwives who were qualified nurses completing an 18-month preregistration course (n=8)  Convenience sample from 3 sites (rural-urban / commuters / students) | Qualitative study  Cross-sectional  Multi-site | Psychosocial well-being during the postpartum period | Focus groups with 3 open ended questions  Audio-recorded but not transcribed verbatim (time and financial constraints) - another author listened to the tapes and checked the notes  Thematic analysis based on the audio tapes and observational notes (methods not detailed) | Wellbeing was defined as the extent to which the parent seemed to be coping during the postpartum period (combination of coping abilities and unmet / unrealistic expectations related to childbirth, child's behavior and disruption to the parents' lives)  Wellbeing assessment include: observation, interviewing skills and labour debriefing. Previous contact is a facilitator.  Worrying behaviors include: high expectations, high levels of anxiety or absence of midwife / parent interaction | NA | (1)  1: Y  2: N  3: N  4: N  5: N  Low |
| Hauck et al., 2015  Australia | 238 hospital midwives working in a hospital.  23.8% reported having worked in psychiatric setting.  47% reported a training on mental health within past 2 years.  Convenience sample  Contact with the 475 midwives employed in the hospital (response rate= 50.1%) | Quantitative descriptive study  Cross-sectional  Single-site | Perinatal mental health problems (bipolar disorder, anxiety, depression, schizophrenia, tocophobia, personality disorders and SUD) | Self-designed questionnaire assessing: midwives' professional development needs (6 items), general knowledge (13 items questionnaire), identification of PMHD (response to 4 clinical vignettes depicting depression, schizophrenia, bipolar disorder and anxiety - "is there something wrong with the person in this vignette?"), attitudes toward mental health (midwives' role and beliefs about parents with PMHD)  Face validity tested with 7 midwives not employed in the study setting  A priori sample size calculation= 213 participants | 37.6% of midwives reported being well equipped to support parents with perinatal mental health problems (principal need for education= personality disorders). Other needs included bipolar disorder, SUD, tocophobia, schizophrenia and anxiety.  Main needs: additional training on the impact of childbearing on mental illness and service options available. Other needs include: handling stress and aggression, assessment of mental health, assessment of risk of mental illness and clinical management. Preference for online formats.  51.9% had knowledge below median scores. Knowledge about EPDS below 50%. Higher identification for depression (93.9%) than schizophrenia (65.6%)  No influence of level of comfort, years of experience, previous training or experience in psychiatry and frequent work with parents with mental illness on knowledge scores (questionnaire and vignettes)  Previous work experience in psychiatry and correct identification of bipolar disorder vignette predicted the perception of being well-equipped  Positive self-report attitudes towards serious mental illness (83% likely to recover, 14% should not be encouraged to have children)  Cluster analysis : more negative attitudes towards parents with mental illness compared with parents with physical disability | NA | (4)  1: Y  2: Y  3: Y  4: N  5: Y  High |
| Higgins et al., 2017  Ireland | N=186  79.6% midwives  60% reported training on perinatal mental health (for a majority during their nurse/midwifery training).  Convenience sample Estimation of response rate: 13.6% of national public health nurses  No sample size calculation | Mixed-method study  Cross-sectional Online survey  2 open-ended questions for qualitative analysis | Perinatal mental health problems (perinatal depression, perinatal anxiety, BP, postpartum psychosis, schizophrenia, OCD, SUD, PTSD) and domestic violence  Pregnancy and postpartum period | - Self-designed online questionnaire assessing self-report  knowledge about PMHD (19 items), self-report skills in PMHC (35 items), overall skills and confidence in PMHC (2 items), PMHC practices (23 items), perinatal mental health services and guidelines (6 items)  Descriptive analysis  - 2 open-ended questions related to education and practice priorities.  Content analysis  Coding by two researchers  - Face validity tested with perinatal mental health care experts and specialists (reviewing and feedbacks) | Self-reported knowledge above average only for perinatal depression.  Self-reported knowledge below average for perinatal anxiety, impact of PMHD on parent and baby, risk factors, available support services and screening tools. Lowest scores for personality disorders, OCD, eating disorders, self injury and suicide, bipolar disorder, SUD and legal aspects  Self-report skills above average for asking about depression or anxiety, discussing referral and developing a care plan  Self-report skills below average for asking about / discussing referral or developing a care plan in other conditions (suicide, SUD, eating disorder, psychosis)  Self-reported skills above average for providing support to parents with childbirth trauma or emotional distress  Self-reported skills below average for providing support to parents with concerns about antipsychotics during pregnancy or hereditary nature of mental health problems  Between 80% and 90% of public health nurses reported assessing perinatal mental health. 70 to 80% reported never asking about eating disorders. Half never asked about SUD, and between 30 and 40% never asked about psychosis, suicide or self-injury thoughts, anxiety or OCD  73% self-reported identifying parents at risk of PMHD and 70% used a screening tool.  Higher self-report knowledge, skills and confidence for those who received training compared with others  Educational needs on knowledge of PMHD (types, risk factors, medication issues, etc.), skills (interview, assessment, support and counseling) and regular, up-to-date education from mental health specialists with role-play. | NA | (1)  1: N  2: N  3: N  4: N  5: N  (4)  1: N  2: N  3: N  4: N  5: Y  (5)  1: N  2: N  3: N  4: N  5: N  Low |
| Higgins et al., 2018a  Ireland | N=809  54.1% hospital midwives  53.1% received education on perinatal mental health (mostly during their training).  Convenience sample  Estimation of response rate between 10.3 and 26.5% | Quantitative study  Cross-sectional  Online survey | Perinatal mental health  Pregnancy and postpartum period | Self-designed questionnaire related to 26 providers- and system-level barriers to providing PMHC  Face validity: minor changes after feedbacks of PMH providers  Descriptive study (group differences between midwives and nurses / between providers with training on PMH and those without). | System/ organizational barriers to discuss mental health issues  = lack of time, lack of clear referral pathway, discontinuity of care, unavailability of PMH services  Providers barriers : lack of knowledge to discuss mental health issues, lack of skills in case of a positive answer, fear of negative reaction, risk of self-harm / suicide, discomfort related to the presence of a partner, stigma  No statistical differences between midwives and nurses in self-report barriers to discuss mental health related to knowledge or skills  Compared with those who did not received training, reduced self-report barriers related to: lack of privacy, workload, lack of time, lack of available resources, lack of care pathways, feeling of isolation, no role in PMH care, lack of knowledge, lack of skill and lack of knowledge of available support | NA | (4)  1: N  2: N  3: N  4: N  5: Y  Low |
| Işık & Bilgili, 2010  Turkey | N=302  53.6% community midwives  47% received previous training  Sample size calculation: proportion of all 1270 midwives (target=290, sample included=302; 23.8%) | Quantitative study  Cross-sectional  Multi-site | Postpartum depression | Self-designed questionnaire based on the literature  24 open-ended questions assessing knowledge about postpartum depression (symptoms, risk factors and diagnosis; 5 items) and participants' experiences and opinions regarding postpartum depression (10 items)  Grouping of the answers by similarity for quantitative analyses  No construct and content validity | Contrasted results between participants self-reported knowledge and researcher evaluation from their responses (lack of knowledge about postpartum depression definition, signs and diagnosis)  71.5% reported never encounter parents with postpartum depression.  47% received previous information about postpartum depression (initial training for 36%, own life experience for 27.3%)  31% perceived their training as low and 74.3% reported need of training  No significant association between years of experience and knowledge about postpartum depression. Significant association between previous training / previous contact with parents with postpartum depression and knowledge about postpartum depression. | NA | (4)  1: N  2: N  3: N  4: N  5: Y  Low |
| Jarrett, 2014  UK | Student  midwives (n=7)  Convenience sample  Sampling strategy: contact with 60 student midwives and inclusion of 7 midwives (11.7%)  Reasons for not participating not recorded | Qualitative study  Cross-sectional  Single-site | Mental health problems  Pregnancy and postpartum period | Open-ended question related to student midwives' experiences and attitudes when caring for parents with mental health problems  Focus groups conducted by one of the author and a 2^nd^  Member, both closely involved with teaching student  Verbatim digitally recorded and transcribed  Thematic analysis of focus groups (3 to 4 students) using NVivo  Coding not detailed | Use of informal knowledge / intuitions to identify parents with PMHD (attitudes, behaviors or body language, eye contact, responses to the questions or behavior towards baby)  Negative attitude toward standardized questionnaires and screening tools (viewed as not effective; negative attitudes towards qualified staff who used it). Fear to use the terms "depression" and "anxiety" because they felt that it could alienate parents and prevent them to disclose  Use of everyday language ("feeling down") to open discussion about mental health  Cultural and illness stereotypes dominated student perception of parents's answers (e.g. Black Carribean parents could not acknowledge their symptoms as depression)  Stigmatization of parents with serious mental illness (perceived dangerousness, inability to be a parent, fear of some behaviors perceived as deviant). Negative attitudes towards IVF in this population | Cultural and illness stereotypes dominated student perception of parents's answers (e.g. Black Carribean parents could not acknowledge their symptoms as depression) | (1)  1: N  2: Y  3: N  4: N  5: Y  Moderate |
| Jarrett, 2015  UK | Student  midwives (n=33)  42.4%: experience of care of people with mental health issues.  Preregistered nurses completing a shortened  midwifery program  -students with no previous qualification in nursing (57.6%): had no prior experience of care of people with mental health issues.  Convenience sample (61% of preregistered nurses and 100% of students with no previous qualification in nursing) | Quantitative study  Cross-sectional  Single-site | Perinatal mental health problems  Pregnancy and postpartum period | Modified version of a non validated questionnaire used in a study to evaluate midwives' knowledge in PMH (Ross-Davies 2006)  23-itmes questionnaire assessing knowledge of PMHD, screening and identification of PMHD, and experience of caring for parents with PMHD  Face validity assessed by two experts (a statistician and a professor of maternal and child health)  Descriptive analysis (lack of statistical power for group comparisons) | Systematic assessment of PMH at booking visit. High self-report confidence in asking questions about PMH and referral in case of positive answers  No use of screening tools nor standardized scales  Perception that assessment of PMH is part of their role but also that the care of parents with perinatal mental health problems should be carried out by specialist midwives.  Physical wellbeing perceived as the priority for midwives  Lack of confidence and feeling to be ill-prepared when caring parents with serious mental illness (depression, postpartum psychosis, schizophrenia or bipolar disorders).  Feeling of discomfort when caring for parents with SMI (perceived dangerousness for others)  More than half were able to define  postpartum depression, OCD, PTSD but less than 50% were able to define postpartum psychosis or maniac depression  Good knowledge in the frequency of PMHD. Lack of knowledge for postpartum psychosis | NA | (4)  1: N  2: N  3: N  4: Y  5: N  Low |
| Jomeen et al., 2009  UK | Midwives (n=52):  42.3% community midwives,  55.8% hospital midwives  1.9% community and hospital midwives  90% received training about postpartum depression  42% received training about antenatal depression (45.5% pre-registration training and 36% post registration training).  Length of the training: 1 hour to 6 days  Quality of the training: 55% poor, 0% excellent  Convenience sample  Response rate: 21.6%  90.4% complete data  9.6% (n=5): incomplete data | Quantitative study  Cross-sectional  Multi-site | Antenatal depression | Descriptive study  -Adaptation of the Illness Perception Questionnairerevised version (IPQ-R) based on the literature on antenatal depression  -Adaptation of the Beck Depression Inventory (BDI).  Discussion of the modified items with clinical midwives who did not participate to the study  Responses were used descriptively rather than as scaled scores | Most midwives cared for parents with antenatal depression. 25% of these parents were not referred to other services  Awareness of antenatal depression symptoms but overlap with those commonly reported by parents  40% reported no clear understanding of antenatal depression  Lack of knowledge about evolution and consequences. 90% considered antenatal depression as treatable | NA | (4)  1: N  2: N  3: N  4: N  5: N  Low |
| Jones et al., 2011  Australia | N=815  46.4%: midwives  53.6%: registered nurses and midwives  Convenience sample Response rate = 81.5% (recruitment of current practice midwives members of Australian College of Midwives) | Quantitative study  Cross sectional  Postal survey | Perinatal depression | Self-designed questionnaire based on the Health professional knowledge questionnaire used in the beyondblue's National Baseline Survey, the existing literature and the DSM-IV revised  20 multi-choice items questionnaire assessing knowledge of perinatal depression (onset incidence, co-occurring conditions, symptoms, associated risk factors, assessment, and treatment options)  Critical review of the questionnaire by 2 maternity researchers  Pilot study of reliability and face validity (n=13 student midwives)  Internal consistency: r=0.69  Analysis of variance and multiple regression analysis | Correct answers = 62.9% for antenatal depression and 70.7% for postnatal depression  Younger age and higher education are associated with greater knowledge about antenatal depression and postnatal depression  Lack of knowledge about perinatal depression (proportion, adverse outcomes, risk factors, recommended treatment options) and screening tools  30.8 % reported adequate training  Learning through on the job experience or conferences / workshops. 2/3 considered that their initial training could have prepared more for caring for parents with perinatal depression. | NA | (4)  1: Y  2: Y  3: N  4: Y  5: Y  High |
| Jones et al., 2012a  Australia | N=815 midwives (registered nurse clinician with midwifery qualification)  Convenience sample  Response rate = 74.3% | Quantitative study  Cross sectional  Postal survey | Emotional distress during the perinatal period (i.e depression, anxiety disorders, bipolar disorder and postpartum psychosis in this study) | Modified version of the 17-item REASON questionnaire assessing attitudes towards midwives role in management of parents with mental health problems  -confidence to assess and manage emotional distress (12 items, Cronbach's alpha=0.82)  -perception of workplace and care of parents with emotional distress (5 items; Cronbach's alpha=0.73)  Idem Jones et al. 2011  Scale validation during the study | Scale validation: factor analysis  55.9% of participants reported having time to routinely assess parents's emotional distress  The majority of midwives reported that they had a primary role and could make a difference to parents with depression or anxiety. 72% felt comfortable in questioning parents about emotional disorders and 82% did not consider it as intrusive. 80% felt confident in referral but 1/3 perceived themselves as competent to provide counseling or relaxation interventions  45.8% reported that they felt comfortable treating physical and emotional problems. | Inclusion in the five year national  framework for the National Perinatal Depression Initiative | (4)  1: Y  2: Y  3: Y  4: Y  5: Y  High |
| Jones, Creedy and Gamble, 2012b  Australia | N=743 current practicing midwives  Convenience sample  Response rate = 74.3% | Quantitative study  Cross sectional  Postal survey | Perinatal depression | -Self-design questionnaire based on the Screening Evaluation Questionnaire used in the beyondblue's National Baseline Survey assessing  -rate of perinatal depression  -perceived barriers and impact in care of parents with perinatal depression  -attitudes towards EPDS  -Clinical vignette depicting perinatal depression, based on previous studies (Jorm et al. 1998, Buist et al., 2005) : questionnaire assessing knowledge (symptoms of perinatal depression, therapeutic options, management) and confidence in management of perinatal depression  Descriptive study  Multiple analysis of covariance assessing impact of educational level, years and type of practice on management | Barriers in care of parents with perinatal depression = lack of time, perceived reluctance by parents to seek help, and lack of support service  Positive attitude toward the use of the EPDS  63.3% diagnosed perinatal depression in clinical vignette  Lack of knowledge in treatment option and negative attitudes towards treatment during the perinatal period  Self-reported good confidence in referral parents to other providers, but lack of referral in practice  No significant impact of educational level, years and type of practice in management responses | NA | (4)  1: Y  2: N  3: N  4: Y  5: Y  Moderate |
| Keng 2005  Malaysia | N=57 midwives  Contact with all midwives of the hospital (n=59), inclusion of 57 participants (reasons for non participation recorded) | Quantitative descriptive analysis  Cross sectional  Single-site | Postpartum depression | Self-designed10-item questionnaire assessing knowledge of postpartum depression (definition, duration, prevalence, risk factors), and their views on the quality of their training  One open-ended question related to midwives' view and personal experience of their practice  Face validity estimated by 4 midwives | Training rated as good but lack of knowledge about postpartum depression (e.g. difference between baby blues and postpartum depression | NA | (4)  1: Y  2: Y  3: N  4: Y  5: Y  High |
| Lau et al., 2015  Australia | N=181  n=95midwives  n=86Maternal Child Health Nurses (MCHN)  17.9% of midwives and 23.3% of MCHN had work experience in mental health setting during nurse education.  30% of these MCHN had a qualification in mental health , but none of the midwives  No training in psychiatry for midwives  Convenience sample | Quantitative study  Cross sectional  Multi-site | Suicide during the perinatal period | Attitudes to Suicide Prevention Scale (ASPS)  Cronbach’s alpha = 0.77  Test–retest reliability=0.85  When >20% of missing data: items not included in the analysis | Compared with MCHN, midwives had more negative attitude towards suicide prevention (significant differences for items related to need of suicide prevention, attitudes when asked to do more about suicide, perceived responsibility in suicide risk assessment, attitude about suicide prevention, and discomfort in assessing suicidal risk)  Compared with those without mental health nursing experience, providers with mental health nursing experience had more positive attitudes towards suicide risk assessment  No influence of age or type of qualification (general nursing vs. direct entry to midwifery education) | NA | (3)  1: N  2: Y  3: Y  4: Y  5: Y  High |
| Madden et al., 2018  Ireland | N=7 midwives (mostly antenatal midwives)  N=1 mental health nurse  Convenience sample | Qualitative study  Cross-sectional  Single-site | Perinatal mental health (from SMI to PMHPs)  Conception to the end of the 1^st^ postpartum year | Qualitative action research (involvement of participants as co-researchers during 3 meetings)  Themes addressed: need for a referral pathway, ideas for a referral pathway, redaction of the referral pathway and agreement about screening questions  meetings were recorded and transcribed by one researcher who also led the meetings  Flexible interview guide  Thematic analysis of verbatim and the reflective journal: involvement of co-researchers in the analysis | Uncertainty and anxiety in decision-making about referral to specialist care (in particular for parents without established diagnoses; anxiety about when deciding to refer)  Importance to discuss PMH issues and to consider parents' preferences in decision-making about referral  Barriers to asking questions about PMH: Lack of time, lack of clear referral pathway and fear of being intrusive  Need for midwives to discuss the meaning of PMH before receiving additional education on PMH (e.g. guidance about the use of the Whooley questions) | NA | (1)  1: N  2: Y  3: N  4: N  5: Y  Moderate |
| Magdalena & Tamara 2020  Poland | N=111 midwives  20% received training about perinatal depression  Convenience sample | Quantitative study  Cross-sectional  Multi site | Perinatal depression (pregnancy to the 1^st^ year after childbirth) | Self designed questionnaire assessing perceived knowledge and skills in parents health assessment (including mental health) required by a new Polish standard for perinatal care  20-items Test of Antenatal and Postpartum Depression Knowledge (Jones 2011) assessing knowledge of perinatal depression (onset, prevalence, co-occurring conditions, symptoms, risk factors, assessment and treatment options)  Clinical vignette depicting perinatal depression (Buist 2006) with open ended question about diagnosis and management  Intergroup comparison  Multiple regression analysis | Compared with other duties related to physical health, midwives reported lower perceived competence (knowledge and skills) in the assessment of a parent's mental health.  Significant effect of training about the screening and management of perinatal depression on midwives' perceived competence  Lack of knowledge about perinatal depression (more pronounced for antenatal depression than postpartum depression; in the use of EPDS)  Shorter work experience (in general) was associated with higher knowledge about perinatal depression  Shorter work experience as a midwife was associated with higher knowledge about postpartum depression (not significant for antenatal depression)  Clinical vignette: 60.4% of correct answer for antenatal depression and 64% for postpartum depression. Higher knowledge was associated with case identification and more positive attitudes towards referral to mental health specialists  Age and work experience as midwife were negatively associated with positive attitudes about the usefulness of support groups for young parents | Implementation of a national policy about screening and management of perinatal depression | (4)  1: N  2: N  3: Y  4: Y  5: Y  Moderate |
| McCann & Clark, 2010  Australia | N=38 1^st^ year student midwives (direct-entry preregistration Bachelor of Midwifery)  48% reported caring for patients with schizophrenia at least monthly  Convenience sample  No sample size calculation  Response rate= 100% | Quantitative descriptive study  Cross-sectional  Single-site | Schizophrenia during the postpartum period | “Attitudes and Beliefs about Mental Health Problems: Professional and Public Views” questionnaire (clinical vignette depicting schizophrenia and 56-items questionnaire assessing demographic data (6 items), knowledge and attitudes towards serious mental illnesses (47 items related to prognosis and outcomes) and 3 open-ended questions  Reliability determined in other studies (Jorm et al., 2006. Not detailed) | Positive attitudes towards referral to health providers (GPs, counseling, mental health nurses, psychiatrists and psychologists) and support by family members, friends and social workers. Self-management was perceived as harmful  Antidepressants, antipsychotics, and anti-anxiety agents were perceived as more helpful than vitamins, herbal-related medications, analgesics, hypnotics, and antibiotics  Physical activity, getting out, relaxation, counseling, special diet and avoidance of alcohol perceived as more helpful than self-help books, CBT, psychodynamic therapy, hospitalization in inpatient unit, electroconvulsive therapy, hypnosis, use of alcohol for relaxation  Professional help perceived as recovery factor. No professional help was associated with poor prognosis. Receiving help was associated with less negative outcomes and more positive outcomes | NA | (4)  1: N  2: N  3: Y  4: Y  5: 5  Moderate |
| McCauley et al., 2011  Australia | N=161 midwives  4% had mental health nursing qualification  3% had counseling qualification  45% received training about general counseling  45% received training about interviewing skills  20% received no training about mental health assessment and psychopharmacology  Convenience sample  Response rate = 30% | Mixed-method study  Cross-sectional  Multi-site | Serious mental illnesses (depression, anxiety, personality disorders, bipolar disorders, OCD, schizophrenia, mania)  Pregnancy and postpartum | Victoria Survey of Midwives (VSM)2000 (McCauley 2004) assessing knowledge, skills, attitudes and experience of caring parents with serious mental illnesses during the perinatal period  Face validity by a panel of experts (n=6 mental health nurse / midwives)  Descriptive study  One open-ended question related to midwives' experience of caring of parents with serious mental illnesses during the perinatal period  Thematic analysis (analysis by 2 academics who coded data independently and then jointly) | Midwives reported to frequently encounter depression and anxiety  Four more important skills: communication skills, teamwork, rapport building and grief counseling  Four least important: mandatory reporting, suicide assessment, relationship counseling, and assessment of mental health status  While mental health was part of assessment of a parent's wellbeing, midwives reported to feel neither comfortable nor confident to work with parents with serious mental illness  Lack of knowledge about available resource. Barriers to referral = lack of resource availability, lack of awareness of other providers' role, lack of awareness and/or confidence in the competences of other professionals, desire to protect parents from labeling  50% felt that parents with SMI needs were not always met in maternity services. More than 60% reported negative attitudes towards parents with SMI (e.g. avoiding these parents). 42% reported positive experiences in working with these parents  93% reported needs of training about skills and knowledge (screening, specific care, mental health interventions). This included training about PMHD and available resources but also multidisciplinary work and feedback from MH providers after referral  61 midwives responded to open-ended questions:  Negative experiences: lack of knowledge, skills and understanding of parents's needs, perceived dangerousness for others (the baby, other patients and themselves), lack of time and belief that these parents are difficult to treat and should be treated elsewhere.  Positive experiences = value that listening to these parents had for midwives (e.g. learning from these parents' experience). Possibility to identify parents' concerns and to provide adequate care | NA | (1)  1: Y  2: N  3: N  4: N  5: N  (4)  1: Y  2: N  3: Y  4: N  5: N  (5)  1: Y  2: N  3: N  4: Y  5: N  Moderate |
| McGlone et al., 2016  UK | N=8 midwives (inclusion criteria: conducting antenatal booking visits).  One received a one-day training about Whooley questions  Convenience sample | Qualitative study  Cross-sectional  Single-site | Perinatal depression | 18 open-ended probes related to attitudes and experiences of using the Whooley questions  Interpretive research  Digitally audio-record of interviews and verbatim transcription  Framework analysis by one researcher  Validity checking by a second researcher | Negative attitudes toward the Whooley questions (no clear understanding of their purpose, questions perceived as inappropriate). Discomfort when disclosure occurs  Other barriers: lack of knowledge about perinatal mental health problems and referral pathways, lack of time and dissatisfaction because not being able to fulfill their role  Midwives relied on their professional / personal experiences | NA | (1)  1: N  2: Y  3: N  4: N  5: N  Low |
| McGookin et al., 2017  UK | Quantitative part: n=25 student midwives (16% response rate with complete data)  Qualitative part: n=7 student midwives (2 first-year, 2 second-year and 3 third-year)  Convenience sample | Mixed-method study  Cross-sectional  Single-site | Antenatal anxiety | First part**:** online survey:  17-items questionnaire based on the NICE guidelines(awareness, knowledge and experience)  Face validity by a midwife involved in the midwifery BSc program  Descriptive statistics (lack of statistical power)  2^nd^ part**:** qualitative study  Interviewing guide designed from the results of the 1^st^ part of the study  Open-ended questions related to awareness, knowledge and experience with managing antenatal anxiety. Thematic analysis led by one researcher (independent coding by another researcher). Discussion with the rest of the research team (midwives and psychologists)  No member-checking with participants | First part :  Lack of knowledge about antenatal anxiety (awareness of the existence of NICE guidelines but lack of correct identification and lack of knowledge about treatment options)  Combination of course-based methods and improvement of clinical skills perceived as the best training  2^nd^ part:  Awareness of midwives' roles in managing perpetuating factors (social support, comparison to an ideal, past experiences, medical care)  Barriers to screening= negative attitude towards screening (not a priority; perception that anxiety is normal during pregnanc), lack of time and lack of specialist midwives, lack of confidence in participants' own knowledge and skills  Perceived skills required to assess antenatal anxiety = communication skills (confidentiality, ability to reassure the parent, honesty and use of non-medical language), including family during antenatal classes and knowledge of the referral pathway  Need of integration of mental health on midwifery course (role of midwives in management of antenatal anxiety more than knowledge) and placement with specialist mental health midwives | NA. | (1):  1: N  2: Y  3: Y  4: N  5: N  (4):  1: N  2: N  3: N  4: N  5: N  (5):  1: N  2: Y  3: N  4: N  5: N  Low |
| Noonan et al., 2018  Ireland | N=157 midwives Response rate = 36.7%  74.5% nurses and midwives  25.5% midwives  1.4% registered psychiatric nurse  13.4% received training about perinatal mental health  63.1% known a person with history of mental health  Convenience sample | Quantitative study  Cross sectional  Multi-site | Perinatal mental health problems (perinatal depression and anxiety, OCD, PTSD, eating disorders, bipolar disorders, borderline personality disorders)  From conception to the 1^st^ year after childbirth | Peripartum Mental Health Questionnaire (PMHQ) : 4 subscales on awareness (9 items), causes (17 items), consequences (7 items), professional issues (7 items)  Cronbach'sα ranging from 0.57 to 0.81  -Mental Illness: Clinicians’ Attitudes (MICA-4): 16 items  Cronbach'sα= 0.72  -One question about perceived training needs (derived from Hauck et al., 2015)  5 open-ended questions related to availability of guidelines and referral options, use of screening tools and past and current symptoms of PMHD  Face validity (n=10 midwives)  Descriptive analysis | PMHA:  Self-perception to be knowledgeable on stress, anxiety and depression = 63.2% / very knowledgeable= 8.4%  Self-perception to be confident in identifying stress, anxiety and depression = 59.4% / very confident= 13.5%  Significant association between perceived knowledge and perceived confidence (identification and management of PMHPs)  Lack of knowledge about risk factors of stress during pregnancy ; agreement that  PMHD had major consequences for parents and on maternal fetal attachment  Self reported good knowledge about available resources. Barriers to screening= Lack of time and feeling of discomfort about discussing mental health  MICA-4:  Positive attitudes towards working with a colleague with SMI (negative attitudes about disclosure if concerned - anticipated stigma). Ambivalence about perceived dangerousness: low perceived dangerousness for others but most described the need that the public should be protected from people with mental illness  Personal development:  Most felt ill-equipped to support parents with PMHD.  Lack of knowledge about PMHD (and in particular depression and schizophrenia), lack of skills for mental health assessment and about the assessment of risk of mental illness  Open questions:  Less than half had access to guidelines  Mental health is assessed (previous history, current symptoms) but not with validated screening tools  Referral to psychiatric liaison nurse and to psychiatrist were the most common referral option | NA | (4)  1: N  2: N  3: Y  4: N  5: Y  Moderate |
| Noonan et al., 2019  Ireland | N=105 public health nurses (PHN)  74.3% registered midwives  66.3% reported a personal experience of mental health issues (friends, family or self).  24.8% received continuous professional development courses on perinatal mental health  Convenience sample- 46.2% of the 227 eligible participants | Mixed method  study  Cross sectional  Multi site | Perinatal mental health problems (depression, anxiety, to SMI) | Peripartum Mental Health Questionnaire (PMHQ) : 4 subscales on awareness (9 items), causes (17 items), consequences (7 items), professional issues (7 items)  Cronbach's α ranging from 0.57 to 0.81  -Mental Illness: Clinicians’ Attitudes (MICA-4): 16 items  Cronbach's α= 0.72  -6-items questionnaire about perceived training needs (derived from Hauck et al., 2015)  -5 questions related to PHN access to perinatal mental health guidelines, current screening practices and available referral options  Descriptive statistics  Thematic analysis of a open ended question asking if participants had something to add; independent coding by two researchers  Face validity (n=10 public health nurse) | Self-perception to be knowledgeable/ very knowledgeable in recognition of stress, anxiety and depression = 83.8% / 77.2%  Significant association between perceived knowledge and perceived confidence (identification and management of PMHPs)  No significant association between age, level of education, perinatal mental health education, frequency of contact with parents with PMHD, having personal experience of mental health with the level of confidence to manage parents with PMHD  26% felt well equipped to support parents with PMHD. Lack of training on cultural diversity  Positive attitudes towards parents with serious mental illness  Open questions:  53.3% had access to guidelines  Mental health is assessed (previous history, current symptoms) but not with validated screening tools (only 38%)  Referral to GP, community mental health nurses, and counseling  Thematic analysis  Personal experience of PMHD allow to relate to parents experiencing the same problems and to reduce stigma (2 participants); other themes: need for continuous education, clear referral pathways; engagement in perinatal mental health conversations. | NA | (1)  1: N  2: N  3: N  4: N  5: N  (4)  1: N  2: N  3: Y  4: N  5: Y  (5)  1: N  2: N  3: N  4: Y  5: N  Low |
| Nyberg et al., 2010  Sweden | N=8 midwives with experiences in specialized clinics for parents with posttraumatic stress symptoms.  Some received training in posttraumatic stress, counseling or skills training in cognitive-behavior therapy (number not recorded)  Convenience sample  8 of 15 midwives that were eligible (53.3%; reasons for non participation not recorded) | Qualitative study  Cross-sectional  Single-site | Posttraumatic stress  symptoms after childbirth  Prenatal period | Open-ended probe related to midwives' experiences during an encounter with parents with posttraumatic stress symptoms.  Interviews were tape-recorded and transcribed verbatim. No mention of independent coding  Thematic analysis | Midwives became aware of the consequences of childbirth trauma on parents' lives, parent-baby interaction and on a 2^nd^ pregnancy (bonding and anxiety)  Midwives' role: to make space for the parents to express their emotions (i.e. to be present, to listen, to respect the parent's fear, to confirm their experience, to show empathy and to support parents to give birth again). Need for supervision to manage their own emotions  Importance of support during childbirth to go past previous negative experiences (e.g. feeling of being abandoned, not supported and not treated with respect) | NA | (1)  1: N  2: Y  3: N  4: N  5: N  Low |
| Oni et al., 2020  Australia | N=18 midwives  12 outpatient clinics  6 worked in specialized units  Convenience sample  Reasons for non participation not recorded | Qualitative study  Cross-sectional  Multi-site | SUD  Pregnancy | Open-ended probes related to barriers and facilitators to screening and referral of pregnant parents with substance use disorders  Minor modification after the first interviews.  Face-to-face interviews (n=13) and telephone interviews conducted by the 1^st^ author.  Tape-recorded and transcribed verbatim by the 1^st^ author  Data saturation after 13 interviews  Thematic analysis  Independent coding by two researchers | Barriers to screening and referral:  -lack of validated screening tools  - inadequate support and training (interviewing skills, available resources in particular in rural areas)  - discomfort in screening: fear of being judgmental, fear of offending and embarrassment and lack of clear referral pathways  -lack of time and workload  -parents's reluctance to disclose SUD due to stigma (fear of being judged and social services involvement) ; reluctance and non adherence to referrals  Facilitators:  -parent centered care  -midwives awareness of consequences of substance use on the babies  -experience and training (how to initiate a discussion about SUD)  -continuity of care  -Available resources | NA | (1)  1: Y  2: Y  3: N  4: N  5: N  Moderate |
| Phillips, 2015  UK | Student midwives (n=9)  2 received training about counseling on perinatal mental health or had cared for people with serious mental illness  Convenience sample | Qualitative study  Cross-sectional  Single site | Perinatal mental health problems (PMHPs) | Open ended questions related to students' experiences of working with parents with PMHPs  Digitally recorded and transcribed verbatim  Thematic analysis of 2 focus groups led by 2 researchers  Coding by one researcher. Section on reflexivity and influence of the study setting on the results | Sensitivity towards parents with PMHPs (including parents with depression, schizophrenia, bipolar disorder). Awareness of stigma related to PMHPs and cultural issues (ethnicity issues and social pressures related to the parenting role)  Discomfort when working with parents with PMHD (fear and confusion; lack of training, perceived dangerousness for others and the baby, lack of knowledge about the referral process and Whooley questions)  View that qualified midwives fear of going beyond a medical view of pregnancy and labour (e.g. avoiding asking questions about mental health; lack of sensitivity when discussing mental health issues). Need for more training about mental health during midwives' curriculum : knowledge about mental health problems, referral pathway, and skills (communication and referral) | NA | (1)  1: N  2: N  3: Y  4: N  5: Y  Moderate |
| Ross-Davie et al., 2006  UK | N=187 midwives  60.4% registered nurses and midwives  29% received no training about mental health during their preregistration education, and 13% received post-registration training  19% placement in psychiatric wards or mother-baby unit  69.5% described their training in perinatal mental health as insufficient  Convenience sample  72% worked with parents with mental health problems in the last year | Quantitative study  Cross-sectional  Single-site | Perinatal mental health (perinatal depression, bipolar disorder, postpartum psychosis, schizophrenia, personality disorder) | Self-designed pre-training questionnaire (content of the training in Ross Davies et al., and post training results in Elliott et al.)  Assessment of midwives' knowledge, confidence, experience and attitudes related to perinatal mental health.  Descriptive study (methods not described) | Most midwives asked about mental health history at booking and felt confident doing so. 48% expressed concerns in case of positive answer. Positive change in the feelings about referring parents to mental health services after the implementation of a specialist team.  90% considered psychological care as part of their role (94% as important as physical health) 75% reported limited ability to fulfill this role due to lack of time, knowledge and skills  Lack of knowledge about PMHD (heterogeneous, particularly pronounced for postpartum psychosis)  Midwives felt less confident caring for parents with depression or schizophrenia than caring for parents with physical conditions or HIV. 62% felt not confident about caring for parents with serious mental illness  Low perceived dangerousness for others (7%) | NA | (4)  1: N  2: N  3: N  4: Y  5: N  Low |
| Rothera & Oates, 2011  UK | Midwives (n=468; 61%)  Obstetricians (n=24; 3%)  Health visitors (n=276; 36%)  Training on perinatal mental health: 37% initial; 34.2% post-qualification  Convenience sample  Response rate = 26.7% of the 2872 participants | Quantitative study  Cross-sectional  Multi-site | perinatal depression, schizophrenia and postpartum psychosis  Prenatal and early postpartum period | 4 clinical vignettes depicting mild mental health problems with or without previous history, pre-existing serious mental illness and acute psychotic disorder.  Self-designed questionnaire assessing attitudes towards referral / management of perinatal mental health problems  Descriptive study | 2/3 would refer these parents to another provider (midwives / obstetricians more likely to refer than health visitors) - 96% for serious mental illness  Other options: increased number of visits and providing extra support  Referral to GPs in case of mild mental health problems; Mother baby units (MBU) for acute psychosis (3/4) and community perinatal mental health team for SMI (50%)  Reasons to refer :  Lack of knowledge / skills  Negative attitude towards their role in management of perinatal mental health problems (more in obstetricians compared with midwives / health visitors)  85% need for specialist training  Significant differences in the extra help needed between mild mental health problems and serious mental illness: skills development, clear referral pathways, training to improve recognition | NA | 1: N  2: N  3: Y  4: N  5: Y  Moderate |
| Salomonsson, Wijma & Alehagen, 2010  Sweden | N=21 midwives with more than 2 years of experience as midwives  Response rate = 43.7% (reasons for non participation: shortage of time or no eligible time for the interview) | Qualitative study  Cross-sectional  Multi-site | Severe fear of childbirth (FOC)  Antenatal period | Open-ended question related to midwives' experiences of FOC, consequences of FOC and midwifery care in FOC  At the end of the focus groups: questions related to demographic data and two 5-point-likert-type scales about feeling during the focus group and possible changes in the view of FOC.  Focus groups conducted by the 1^st^ and the last authors (two midwives with no professional or personal relationship with participants.  Tape-recorded and transcription verbatim  Phenomenological interpretative analysis  Coding of the 1^st^ focus group both separately and together by the 1^st^ and the last author and checked by the 2^nd^ author. The other focus groups were analyzed by the 1^st^ author and thereafter read by the 2^nd^ and 3^rd^ authors. | FOC described as a continuum from the reasonable fear that nulliparous parents could feel and unrealistic fear related to personal experience of childbirth. Some midwives perceived increased prevalence of FOC but not all. Reasons: young parents dare to express their fears; midwives have a more accepting attitude; increased amount of information on internet contributing to unrealistic expectations.  Contributing factors: negative birth experiences (obstetrical complication or negative experience with staff), fear of losing control, uncertainty about the baby health, fear of parenthood  Consequences: negative pregnancy experiences, avoidance of childbirth classes, strain in the relationship with the partner, increased requests for C section, more complications during labour, economic consequences, negative effect on breastfeeding and parent-baby attachment, starting point for depression  Emotionally demanding and time consuming for midwives who sometimes express counter-attitudes towards these parents  Identification during antenatal visits but also during labor. Needs for individualized care (preparation for labor, supportive attitude, postpartum follow up) | NA | (1)  1: Y  2: Y  3: Y  4: Y  5: N  High |
| Salomonsson, Alehagen and Wijma 2011.  Sweden | N=726 midwives  Antenatal only: n=188, 26%  Labour ward only: n=287, 40%  Mixed work context: n=117, 16%  neither antenatal nor labour ward: n=134, 18%  Compared with other groups, antenatal midwives were older and had more years of experience  Random selection of 1000 midwives out of 4898 eligible midwives  Response rate = 84% (72.6% responding to inclusion criteria) | Quantitative study  Cross-sectional  Multi-site | Severe fear of childbirth (FOC)  Antenatal period | Self-designed questionnaire assessing midwives' views on severe FOC  (6 items on demographic data; 11 items general statements about severe FOC; 15 items about severe FOC during pregnancy; 16 items on severe FOC during labour  Definition of severe FOC provided in the introduction  Face validity = pretest by 6 midwives. No evaluation of the psychometric properties  Multivariable logistic regression | Positive attitudes towards severe FOC (more than 50%)  Compared with antenatal midwives, labour ward midwives had higher self-perceived knowledge and skills in recognition and management of severe FOC (but lower perceived need for specific training; lower perceived responsibility in identifying parents with severe FOC)  Significant predictors in multivariable regression: work context, work experience and presence of a specialist FOC team. No influence of mixed work context | NA | (4)  1: Y  2: Y  3: N  4: Y  5: Y  High |
| Sanders, 2006  USA | N=378  98.7%certified nurse-midwives  1.3% certified midwives  Convenience sample (annual meeting of the  American College of Nurse Midwives (ACNM))  Sample size calculation: 253 participants for 80% power to detect a small effect size  Response rate = 42.6% (378 out of 887 eligible participants) | Quantitative study  Cross-sectional  Multi-site | Substance abuse and depression during the perinatal period | Self-designed33-item questionnaire assessing knowledge about the ACNM position statement on depression and screening practices ; attitudes towards screening and perceived ability  Face validity tested by 9 experts (midwives, nurses and biostatistician), psychometric properties tested in 10 participants  Overall internal consistencyα= 0.83 (0.73 attitudes and 0.64 perceived ability)  Descriptive statistics / multivariable analysis on screening practices | Positive attitudes towards depression screening (universal routine screening) and use of screening tools ; perceived skills in depression screening (91%) and management of parents with perinatal depression (59.3%); 25.1% reported to always screen for depression (61.1% for substance use disorders)  91.8% of the midwives had authorization for prescribing psychotropic medications (65.9% reported to prescribe psychotropic medications)  Low knowledge about the 2002 ACNM position statement on depression screening (12.2%) / the 2002 US preventive services taskforce recommendations on screening for perinatal depression (13.5%)  Significant predictors of depression screening: positive attitude towards depression screening, high knowledge, high perceived ability and education level | NA | (4)  1: Y  2: N  3: Y  4: N  5: Y  Moderate |
| Savory et al., 2022  UK | Quantitative part  N=145 midwives  9% had placement experience in a mental health setting  Qualitative part  N=15 midwives,  1^st^ focus group: 6 midwives from a specialist community team supporting parents with SMI or SUD  2^nd^ focus group: 5 newly qualified midwives  3^rd^ focus group n=4 self-selected midwives  Convenience sample  Response rate = 58% (145 of 250 eligible midwives) | Mixed-method study  Cross-sectional  Single-site | Perinatal mental health | Quantitative part of the study:  Modified version of the 22 items questionnaire from McCauley et al. 2011 (Victoria Survey of Midwives) assessing knowledge, skills, attitudes and experience of caring parents with serious mental illnesses during the perinatal period  Minor changes to adapt the questionnaire to the UK context (face validity tested by 3 midwives)  Qualitative part of the study:  Open ended questions related to midwives' experience of caring for parents, conversations with parents about perinatal mental health and knowledge about support options / service provision  Tape-recorded and transcription verbatim  Coding by one researcher (independent coding of one transcript by a 2^nd^ researcher)  Thematic analysis | Quantitative part of the study:  Most midwives had experience of caring for parents with depression (87%) or anxiety (95%). Informal screening practices (> 80%) based on intuition or clinical experience. Most midwives could refer parents to the GP, health visitors or community mental health teams  31.7% received training on perinatal mental health (mainly on substance use disorder) - low training rates on mental health assessment (11%), psychopharmacology (2.8%), and suicide risk assessment (3.4%).  80% rated the training as important (21.4% rated it as useful in their clinical practice). Skills acquisition during clinical practice (86.9%) and from peers (39.3%) rather than trough formal education (53.1%). 94.5% reported they could be better prepared and 53% reported to feel confident in supporting parents with mental health problems  Qualitative part of the study:  Agreement with NICE recommendations about routine screening of perinatal mental health at each visit. Fear of not "doing it right" even for specialist midwives - no formal training on how to ask the questions (individual decisions / ways of opening conversations)  Facilitators / barriers to screening: lack of time, continuity of care, relationship with parents  Unclear referral pathways for parents with complex issues; most parents with perinatal mental health problems were supported by midwives (awareness of NICE recommendation about referral to GPs but negative attitudes in some midwives). Midwives reported to prefer supporting parents' mental health themselves rather than referring them to other providers (but lack of confidence in case of disclosure of mental health problems)  Positive attitudes about parents with depression or anxiety. Few experiences in caring for parents with serious mental illness in newly qualified midwives - lack of knowledge and negative attitudes in all midwives including specialist midwives (absence of specific training). Lack of training about antenatal mental health  No clear differences between experienced midwives and newly qualified midwives. Training needs : how to initiate conversations about perinatal mental health and information on medication / side effects | NA | (1)  1: Y  2: Y  3: N  4: N  5: N  (4)  1: N  2: N  3: Y  4: N  5: Y  (5)  1: Y  2: N  3: N  4: N  5: N  Low |
| Schouten et al.,2021  The Netherlands | Qualitative part :  N=8 midwives (with > 20% Moroccan Dutch clients)  N=6 Moroccan-Dutch parents with perinatal depression (80% pregnant) - some were clients of the participating midwives  A priori data saturation set at 6 participants  Convenience sample  Quantitative part  N=60 midwives of 450 eligible participants  Convenience sample(response rate =13.3%); reasons for not participating to the survey = lack of time, insufficient experience with communicating about depression with Moroccan-Dutch and Turkish-Dutch parents | Mixed-method study  Qualitative study  Quantitative study based on the 14 barriers identified in the qualitative study  Cross-sectional  Multi-site | Perinatal depression | Qualitative study  open-ended questions related to perceived communication barriers about perinatal depression in Moroccan-Dutch parents  Interviews were led by 2 research assistants (midwives) and a trained female Moroccan-Dutch research assistant (parents)  Transcription verbatim and independent coding by 2 researchers. 3 midwives gave feedback on their transcripts.  Quantitative study:  Self designed questionnaire assessing midwives' perception ofthe extent to which the 14 barriers identified in the qualitative part of the study were a barrier during consultations / occurred during the consultations  Quality Index Indices (QII) calculation based on the QUOTE methodology (mean barrier rating X proportion of participants who frequently encountered that barrier)  QII scores ≥0.60 indicate a potential barrier  QII scores ≥1.75 indicate a influential barrier | Qualitative study  14 communication barriers  Client-related barriers: insufficient Dutch language proficiency (and need for translation by the partner), cultural barriers (taboo about mental health problems in Moroccan / Turkish culture; religious coping), presence of the partner, feeling of shame and lack of trust in health providers  Midwives-related barriers: lack of time and lack of intercultural communication skills , lack of knowledge about depression, perception that this is not their role / responsibility  Education-related barriers : lack of culturally sensitive screening instruments for perinatal depression / available adequate educational materials on perinatal depression (for parents and partners)  Quantitative part  Most influential barriers according to midwives: lack of culturally sensitive screening instruments (QII=3.04) / educational material about perinatal depression (QII=3.47)  Other influential barriers: stigma, feeling of shame, somatizing symptoms  No midwife related influential barrier (lack of time ; QII=0.93; other barriers were lack of intercultural communication skills and lack of knowledge about perinatal depression) | Cultural barriers (taboo about mental health problems in Moroccan / Turkish culture; religious coping)  Lack of culturally sensitive screening instruments for perinatal depression / available adequate educational materials on perinatal depression (for parents and partners) | (1)  1: N  2: Y  3: N  4: Y  5: Y  (4)  1: N  2: Y  3: Y  4: N  5: Y  (5)  1: Y  2: Y  3: N  4: Y  5: Y  High |
| Shahid Ali et al., 2023  Pakistan | N=10 midwives with diploma in midwifery and public health and at least 2 years of experience  Convenience sample  Sample size estimation: n=10 | Qualitative study  Cross-sectional  Single site | Perinatal mental health problems (e.g. perinatal anxiety or depression) | Semi-structured interview translated in Urdu (topics: midwives' perception, understanding, thoughts and feeling towards PMHP and their experiences and roles in identifying and managing parents with PMHP).  Tape-recorded and transcription verbatim  Content analysis (process not detailed) | Lack of knowledge and skills in screening and managing parents with PMHP. Perinatal mental health considered as important (positive effect of the interviews). Cultural factors influence stigma experienced by parents with PMHPs (barrier to disclosure). Midwives reported that parents with PMHP preferred faith healers than specialist mental health care | Cultural myths about PMHPs: resulting from evil eye / supernatural powers; PMHPs is considered as being insane  Preference for faith healers than specialist mental health care | (1):  1: Y  2: Y  3: N  4: N  5: N  Low |
| Stewart & Henshaw, 2002  UK | N=266 midwives (of 377 eligible midwives from two health districts in UK - one with a perinatal psychiatry service and one without  Convenience sample  Response rate = 70.5% | Quantitative study  Cross sectional  Multi site | Perinatal mental health problems (PMHPs) (postnatal blues, perinatal depression, postpartum psychosis) | Self-designed questionnaire assessing midwives' knowledge (prevalence only) and self-reported experience and skills about PMHD; attitude towards their role and comfort in managing parents with PMHD  No items had more than 6% missing data | Correct answer for the prevalence of baby blues (72.9%). Less than 50% correct answers for other conditions (47% for postpartum psychosis, 30.8% for depression in pregnancy) - underestimation of prevalence (51.8%). No differences between the 2 sites.  Experience with PMHD: perinatal depression (67.2% antenatal depression; 75.5% postpartum depression), postpartum psychosis (41%). 65% reported to lack the necessary skills to care for these parents. 94.3% agreed that midwives had a role in the management of PMHD (34.3% reported to feel confident)  Training: 27.1% assisted to at least an event on PMHD (7.5% > than 1 event). 43% received some training in listening skills or counseling. Training needs: 52.5% knowledge about disorders and treatments, 41.3% listening or counseling skills, 11% about referral pathways. | NA | (4)  1: Y  2: N  3: N  4: Y  5: Y  Moderate |
| Willey and al., 2020  Australia | N=24 providers (71% midwives, 12% bi-cultural workers and 17% administration staff)  2 focus groups (n=13) and 8 semi-structured interviews (n=11)  Completed by a survey (63% response rate)  Past training about mental health: 29% of midwives  Past training about cultural aspects: 35% of midwives  None of the administration staff received complementary training  Convenience sample | Qualitative study  Single-site | Perinatal depression / anxiety in parents with refugee background | Open-ended questions related to the implementation of a screening program in parents with refugee background using the EPDS  Use of normalization process toolkit in the interviews / focus groups (four constructs: 1) coherence / meaning making ; 2) participation (community practice); 3) collective action; 4) reflexive monitoring)  Focus groups for midwives. Data collection by a single author, audio recording, transcribed verbatim. Thematic analysis, independent coding by two researchers. No description of data triangulation / reflexivity processes | Positive attitude towards their role in the screening program and the addition of the EPDS to antenatal psychosocial assessment (more focused discussions about PMH). Perceived usefulness of the screening program according to the community-based refugee health and wellbeing service staff (improvements detection and referral; allows more comprehensive assessment using an evidence based tool according to managers)  Perceived usefulness of the EPDS to open discussions about perinatal anxiety ; Screening perceived as acceptable for parents by midwives  Barriers: language issues, lack of time  Facilitators: clear referral pathways after screening , use of m-health tools (more contemporary, easy to use), continuity of care, trust in each other work, support from team managers  Need for additional training in PMH | Perceived usefulness of the presence of bicultural workers | 1: Y  2: Y  3: Y  4: N  5: N  Moderate |
| Williams et al., 2016  UK | N=15 midwives  One had a mental health qualification.  Purposive recruitment of midwives according to the number of referrals made, the size of team they worked in and the proportion of parents with different background they saw  A priori sample size set at 15 interviews for data saturation  N=20 pregnant parents between 12 and 20 weeks gestation  12 primiparous  14 had a history of depression.  Purposive recruitment based on age, socio-economic background, parity and history of depression.  Convenience sample | Qualitative study  Cross sectional  Single-site | Antenatal depression (NICE screening tool: Whooley questions and an extra question on support needs) | For midwives: Open-ended probes assessing their views about antenatal depression (awareness, experiences of screening, current screening practice, views and use of the NICE three questions, referral options and views on treatment options. Interviews led by one researcher (revised after the 1^st^ interview; face-to-face or by telephone)  Tape-recorded and transcription verbatim  For pregnant parents: Open-ended questions assessing their experience of booking appointment and their views and experiences about the 3 questions, views about mental health screening at booking appointment and their previous experiences of depression  One author led interviews (face-to-face interviews at home)  Thematic analysis: coding by the 1^st^ researcher (independent coding of a selection of transcripts by a 2^nd^ researcher) | Midwives:  Screening during the booking appointment is challenging (lack of time, no relationship established). They asked the 3 questions in different manners (directly or using their own words)  Computerized notes made midwives asking the questions (not possible to submit incomplete data). Contexts in which they did not ask the questions: presence of the partner or a medical student, young age, learning difficulties or insufficient proficiency in English. Mental health qualification increased the confidence in asking the questions  Most considered that it did not change their ability to detect antenatal depression (only few reported that sometimes using the questions led to unexpected responses). Usefulness because that it assured parents that midwives were interested in their mental health / facilitating discussions about antenatal mental health. Uncertainty about the usefulness of the extra help question (lack of knowledge about treatment and referral options).  Pregnant parents :  No expectations about being asked about their mental health during the booking appointment - but found it acceptable and welcomed midwives interest in their wellbeing. Desire to identify any mental health issue as early as possible to avoid negative consequences on the baby. Opportunity to discuss anxieties about pregnancy (normalization)  Reasons to confide  To receive support, to prevent negative consequences on the baby, empathic and non-judgmental attitude of the midwife, trusting relationship  Reasons to not confide: stigma, fear of being judged by providers / questioned about their ability as future parents ; fear of not being taken seriously; presence of the partner; reasons for low mood not related to pregnancy  Attitudes towards the three questions  All felt happy about being asked the 3 questions (opportunity to talk about how they felt); some reported blunt questions coming from nowhere; all reported the need for provider initiation of these discussions. Difficulties in understanding the third question about extra help | NA | (1)  1: Y  2: Y  3: N  4: N  5: N  Moderate |
| Whitehead et al., 2019  Australia | N=11 midwives  Convenience sample  Inclusion until data saturation | Qualitative study  Cross-sectional  Single site | Substance use disorder (SUD) during pregnancy | Semi-structured interview guide based on the literature and a consultation with a panel of midwives  Open ended questions related to public health messages provided during pregnancy before funneling down to alcohol use disorder during pregnancy  Face to face or phone interviews led by the 1^st^ author  Tape-recorded and transcription verbatim. Coding by the 1^st^ researcher (discussion of the identified codes with 2 other authors)  Thematic analysis of individual interviews (6 steps protocol; Braun & Clarke 2006)  Critical realist approach  Reflexivity: 1^st^ author female psychologist completing a Master of clinical psychology. The researcher had an interest in supporting at-risk parents but had limited knowledge about midwifery practices or maternity care. No pre-existing relationships between the researcher and midwifery services. Research journal throughout the analysis for reflexivity (values, assumptions and subjective opinions). | - Patient-level factors:  Parents with complex psychosocial issues are less likely to engage in antenatal care (issues in accessing hospital, fear of involvement of child protection services, fear of the hospital environment, lack of knowledge about continuity of care, stigma and stigma about help-seeking)  Need for community-based antenatal care for parents with complex psychosocial issues  - Provider/patient-level factors  trusting relationship (positive effect on disclosure of sensitive information and help seeking), continuity of care  -Provider-level factors  Non-judgmental and supportive approach (casual and conversation assessment style), feeling confident in their role. Level of confidence influenced by work experience as midwife, age, recognition that it's part of their role, training, support from team members and knowledge about sensitive topics. Most used screening tools for SUD or perinatal depression at the booking assessment. Lack of knowledge about the effects during pregnancy and evidence-based strategies to support parents  -Organizational-level factors  Lack of time, lack of support and lack of training (SUD, interviewing skills, referral options, strategies to support parents). Lack of communication between providers in their services. More limited assessment and support in private hospitals compared with public hospitals.  -System level factors:  Lack of referral options, inconsistent messages regarding SUD during pregnancy in the media | NA | (1)  1: N  2: Y  3: N  4: Y  5: Y  Moderate |

**AND:** antenatal depression; **CME**: continuing medical education; **ED**: eating disorders; **FoC**: Fear of childbirth; **GP**: General Practitioners; **MCHN**: Maternal Child Health Nurses; **NA**: not applicable; **OCD**: obsessive compulsive disorder; **PHN**: public health nurses; **PMH**: Perinatal Mental Health**; PMHC**: Perinatal Mental Health care; **PMHPs**: Perinatal Mental Health Problems; **PPD**: postpartum depression; **PPP**: postpartum psychosis; **(PP-)PTSD**: (postpartum) post-traumatic stress disorder; **SMI**: Serious Mental Illness (including: schizophrenia spectrum disorders, bipolar disorders, borderline personality disorders); **SUD**: substance use disorders
