# Supplementary material for: A systematic review of midwives’ training needs in perinatal mental health and related interventions
Source: Front Psychiatry. 2024 Apr 22;15:1345738. doi: 10.3389/fpsyt.2024.1345738 (PMC11071341; doi:10.3389/fpsyt.2024.1345738)
Supplement: Supplementary Table 5 — Characteristics of included studies about midwives' needs for peripartum mental health training program. [file Table_5.doc]

Supplementary Table 5. Characteristics of included studies about midwives' needs for peripartum mental health training program

| Source and country | Population (N, specific training about perinatal mental health, specialization) and sampling | Design and type of study | Training program (duration, type, condition examined, period examined…) | Target skills (screening, communication skills, referral…) | Assessment tools or methods (e.g. scales or probes for qualitative studies) | Key finding | Quality rating (MMAT) |
| --- | --- | --- | --- | --- | --- | --- | --- |
| Badiya et al., 2021  India | One midwife  N=238 pregnant persons | Quantitative study  Cross-sectional  Single-site | Training by psychiatrists, obstetricians and the research team about EPDS (items, scoring mechanism) followed by 3 days of reflective practice and supervision sessions. | Use of the EPDS | Video-recording of the EPDS  during the prenatal visits (24-28 weeks and 36-38 weeks of gestation) and during the postpartum visit (10-14 days and 3 months postpartum)  Concordance between assessments (midwife vs. psychiatrist) on 56 situations | 96% concordance (ICC=0.99) between the two raters | (4)  1: N  2: N  3: Y  4: N  5: Y  Moderate |
| Corse et al., 1995  USA | Nurse-midwives in antenatal clinic (n=7)  All the nurse-midwives who participated in the training program | Qualitative study  Cross-sectional Single site | Prenatal substance use disorder  Duration of training not mentioned  Didactic training involving addiction specialists | Training centered on the consequences of maternal SUD on the fetus ; other targets: knowledge about addictions and treatment  interviewing skills | Probes (not described)  Audio-recorded individual interviews, transcribed verbatim, checked for accuracy and analyzed (constant comparison method) | Behavior changes in interactions with pregnant parents with SUD that participants relate to training into interviewing skills  Improved knowledge, attitudes and skills (less anxiety, feeling of comfort, clients' openness in reporting SUD). | (1)  1: N  2: Y  3: N  4: N  5:N  Low |
| Davies et al., 2016  UK | Student midwives (n=not mentioned)  Convenience sampling | Qualitative study  Cross-sectional Single site | 6 days module about perinatal mental health problems (depression, posttraumatic stress disorder, postpartum psychosis) including SUD, eating disorders and identified risk factors (domestic violence)  Content about midwives and parents wellbeing  Inclusion of a person with lived experience (midwife with lived experience of perinatal depression) during the training | Knowledge, interviewing skills and attitudes toward parents with perinatal mental health problems.  Learning of coping strategies and relaxation techniques | Online anonymous survey of students' satisfaction  Methods not described (2 boxes illustrating the views of 2 participants) | Increased awareness about perinatal mental health and midwives role in improving maternal wellbeing  Improved knowledge and skills | (1)  1: N  2: N  3: N  4: N  5:N  Low |
| Elliott et al., 2007  UK | Midwives (n=235),  Pre-evaluation=187  Complete data at post evaluation, n=73  Convenience sampling | Quantitative non controlled non randomized study  Longitudinal  baseline and one month after training  Single site | Compulsory one-day training on: clinical and research context, video testimonies, perinatal mental health problems (baby blues, postpartum depression, postpartum psychosis, schizophrenia, bipolar disorder), screening, interviewing skills and decision-making on referrals | Knowledge, interviewing skills (study 1)  Improved detection and referral  (study 2) | **Study 1:**  Pre-post 29-items self-report questionnaire designed for the study  (self-rated knowledge, confidence and attitudes towards screening and caring for parents with perinatal mental health problems)  **Study 2:**  Recording of mental health information in maternity handheld notes at postnatal discharge. | **Study 1:**  Improved self report knowledge, confidence and positive attitudes  **Study 2:**  Increased record of the total number of psychiatric disorders | (4)  1: Y  2: N  3: N  4: N  5:N  Low |
| Forrest and Poat, 2010  Scotland | Qualified midwives and specialist perinatal mental health midwives (sample size not recorded)  Sampling not recorded | Qualitative study  Cross-sectional  Single site | 2 compulsory one-day training based on the Scottish Peripartum Mental Health Curricular Framework (Level B and C - qualified midwives and specialist peripartum mental health midwives)  E-learning between the sessions (case discussions)  Peripartum (preconception to postpartum)  Physical and emotional changes that can affect maternal mental health, self-harm, suicide and addiction | Knowledge (mental health problems, treatment options, clients rights, role of practitioners, legal and professional policies)  Skills (therapeutic relationships, working on a value-based way and risk management) | Modified online Objective Structured Clinical Examination and a portfolio of reflective accounts | Self-report satisfaction (e.g. interesting content) but too much work in a limited timeframe | (1)  1: N  2: N  3: N  4: N  5:N  Low |
| Fox et al., 2023  Australia | N=18 midwifery students participating to an Objective Structured Clinical Examination (OSCE) on screening using Edinburgh postpartum depression scale (EPDS)  Response rate : 30% (70 students assessed with the OSCE)  Focus group feedback from confederates (3^rd^ year students) and assessors  N= 16 (50% midwifery staff members and 50% 3^rd^ years Bachelor of Midwifery students) | Mixed-method study  Cross-sectional  Single-site | Objective Structured Clinical Examination (OSCE) simulating a PMH consultation in an antenatal setting using 8 scenarios (depression, anxiety, postpartum psychosis). EPDS scores provided for each scenario.  One week before the OSCE  Use of written scenarios and video testimonies of persons with lived experience of perinatal mental health problems to stimulate ideas for practicing interviewing skills | Perinatal mental health problems | Survey distributed to students after they received results of the OSCE (methods not detailed).  Self-reported level of confidence before and after OSCE in administrating EPDS and in discussing the findings with the parent.  Focus groups of confederates and assessors' feedbacks about their views of the OSCE and their propositions of improvement (no description of the analysis process) | 77.8% of students perceived preparing / participating in the OSCE as useful (improved self-confidence to use EPDS in future placements) and 83.3% reported that their participations of the OSCE was a positive experience  Staff members reported that the OSCE improved students' knowledge and ability to conduct screening using EPDS. Positive effect on the confederates (reflection on their clinical skills by considering screening from the perspective of parents)  Students reported difficulties in knowing how to respond in case of positive answer | (1)  1: Y  2: N  3: N  4: N  5: N  (4)  1: N  2: N  3: Y  4: N  5: N  (5)  1: N  2: N  3: N  4: N  5: N  low |
| Gunn et al., 2006  Hegarty et al. 2007  Australia | Gunn et al., 2006  N=22 (81.8% midwives and 18.2% medical practitioners)  32% reported previous training in counseling and communication skills  Convenience sample  Response rate: 56.4%  Hegarty et al., 2007  n=584 pregnant persons for Pre-ANEW survey  n=481 pregnant persons for Post ANEW survey  No group differences in the frequency of antenatal depression | Gunn et al., 2006  Quantitative study  Longitudinal  Single-site  Hegarty et al., 2007  2 cross-sectional surveys | Antenatal period - psychosocial issues including anxiety and depression  ANEW education program:  26 weeks of 2-hours-session (four interactive workshops, role-play and feedback with simulated patients). Content: person-centered care, interviewing skills, barriers to disclosure / limitations of screening, barriers to implementation | Knowledge  Interviewing skills  Attitudes towards antenatal psychosocial assessment  Confidence  Hegarty et al 2007  Parent assessment of health providers communication skills / confidence in discussing psychosocial issues with health providers | Gunn et al., 2006  Pre and post test (6 months after starting the program) self reported questionnaire  Self-designed questionnaire related to communication skills, willingness to change, learning style, knowledge and attitudes toward psychosocial issues  Hegarty et al., 2007  Self report questionnaires (distinct participants in 2 cross-sectional surveys) | Gunn et al., 2006  Significant positive effect on attitudes towards psychosocial assessment (preference for dealing with clinical issues rather than psychosocial issues).  No effect on the frequency of provider initiation of discussions about perinatal depression or anxiety or perinatal substance abuse (positive effect on history of trauma and concerns about caring for the baby)  Positive effect on knowledge about psychosocial issues, confidence in the ability to open discussions about psychosocial issues, to identify / support parents with psychosocial issues  High participant satisfaction  Hegarty et al., 2007  No effect on parents assessment of health providers communication skills  No effect on parents confidence in discussing psychosocial issues with providers  Significant positive effect on the proportion of parents reporting that midwives asked questions that helped them to discuss psychosocial issues | Gunn et al., 2006  (4)  1: Y  2: N  3: N  4: N  5: N  Low  Hegarty et al., 2007  1: Y  2: N  3: N  4: N  5: Y |
| Higgins, Carroll and Sharek, 2012  Republic of Ireland | N=105 student midwives  Complete data for 79 participants (75% response rate)  Convenience sampling (elective module) | Mixed methods (quantitative descriptive study and qualitative analysis)  Cross-sectional (post evaluation)  Single site | Co-delivering by midwife and a provider with a mental health background  20h module in the 3^rd^ year of a direct entry midwifery program (didactic input and 5 hours of group discussion)  Requirement to read at least one assigned book written by a person with lived experience of postpartum depression /psychosis  Conditions: perinatal mental distress and specific conditions (anxiety, perinatal depression, bipolar disorders, post-traumatic stress disorder, postpartum psychosis) | Knowledge, attitudes towards screening and caring for parents experiencing mental distress  Skills  being present to parents experiencing mental distress) | Questionnaire at the end of the training (closed and open questions) and group presentations  Thematic analysis of textual data by two authors | High participant satisfaction (content and teaching strategies including reading stories of parents with lived experience). Self-report improved knowledge, attitudes and confidence in caring with parents experiencing acute perinatal mental health distress  Suggestions for improvement: switching from an elective to a core module, delivering the training earlier, extended duration, more insight by parents with lived experience | (1)  1: N  2: N  3: N  4: N  5: N  (4)  1: N  2: N  3: N  4: Y  5: N  (5)  1: N  2: N  3: Y  4: N  5: N  Low |
| Higgins et al., 2016  Republic of Ireland | 3^rd^ year midwife students (n=28 for pre-survey and n=26 for post-survey)  53% had a family member with mental health problem, 80% knew someone with mental health problem and 25% (pre-survey) and 31% (post-survey) had themselves had a mental health problem  Convenience sampling (elective module) | Quantitative non controlled non randomized study  Longitudinal (pre and post surveys)  Multi-site | 30 hours module (cf. Higgins et al. 2012)  Conditions: perinatal mental health problems | Knowledge, skills and attitudes towards screening and caring for parents experiencing mental distress | Pre and post survey evaluating self-report midwives' knowledge, skills and attitudes toward parents with mental health problems (Likert scales ranging from 1 to 5) | Significant changes in perceived knowledge, attitudes and skills  No influence of personal or family experience of mental health problems | (4)  1: N  2: N  3: N  4: Y  5: Y  Moderate |
| Hiremath et al., 2016  India | N=50 qualified nurses (Diploma in General Nursing and Midwifery course).  Convenience sample | Quantitative study  Longitudinal (pre and 7 days post intervention)  Single-site | training program on postpartum depression (lectures and group discussion) | Knowledge about postpartum depression | Self-designed questionnaire related to knowledge about postpartum depression (definition, etiology, clinical features, treatment and nurses care) | Significant improvement in self-report knowledge | (4)  1: N  2: N  3: N  4: N  5: Y  Low |
| Hooks 2019  UK | N=48  3^rd^ year student midwives  N=40 at baseline and 29 at post evaluation for the quantitative study  N=10 for the qualitative study  Convenience sample | Mixed method study (quantitative non controlled non randomized study and qualitative interviews)  Longitudinal (pre post)  Single-site | Optional module on SUD during pregnancy ("Substance Misusing Parents")  15-weeks e-learning module with tutorial support  Module with e-learning and tutorial support (one section per week). Inclusion of testimonies of service users on positive and negative experiences of care | Knowledge about, and attitudes related to SUD during pregnancy | **Phase 1**: Jefferson Scale Physician Empathy (JSPE) and Medical Condition Regard Scale (MCRS) in pre and post module  **Phase 2**: semi structured interviews of 10 students recruited depending of their change in scores for Phase 1. Framework method of analysis  **Phase 3:** qualitative content analysis of the posts on the virtual learning discussion board (before and after training). | **Phase 1:** Improved attitudes towards substance misuse during pregnancy (MCRS). No changes in empathy  **Phase 2**: Increased awareness of the negative stereotypes and improved attitudes towards these parents. Positive influence of stories by persons with lived experience. Improved knowledge on SUD  **Phase 3**: influence of personal and practice experiences (e.g. mentor attitudes) on the attitudes towards parents with SUD | (1)  1: N  2: N  3: N  4: N  5: N  (4)  1: N  2: N  3: Y  4: Y  5: N  (5)  1: N  2: Y  3: N  4: N  5: N  Low |
| Jardri et al., 2010  France | 584 eligible parents period 1, n=472 (80.8%)  121 at risk parents (EPDS >10)  120 matched controls (EPDS < 10)  458 eligible parents period 2, n=343 (74.9%)  112 at risk parents  110 matched controls  No significant difference between parents included in period 1 and period 2 (medical and socio-demographic characteristics and prevalence of postpartum depression)  Convenience sample | Quantitative study (one in period 1, one in period 2)  Cross-sectional Single-site | 3-hour training program for midwives about postpartum depression (knowledge about prevalence, risk factors, results of period 1, local recommendations and appropriate use of EPDS) | Case identification and detection of postpartum depression in maternity units | Evaluation before and after training midwives  Period 1: 10 weeks before training  Period 2: 10 weeks after training  Parents included during these periods completed the EPDS between 3 and 5 day postpartum. The same day, midwives completed a clinical assessment questionnaire without knowledge of the EPDS score.  At 8 weeks postpartum, contact by a psychiatrist by telephone (MINI interview) | After training  Significant improvement in case identification (better correlation with postpartum depression diagnosis by a psychiatrist at 8 weeks postpartum) and early detection of postpartum depression (37% improvement) | (3)  1: Y  2: Y  3: N  4: Y  5: Y  High |
| Larkin et al., 2014  UK | Student midwives before registration  Sampling not recorded | Qualitative study  Cross-sectional  Single-site | Blended learning package on perinatal mental health collaboratively developed with two persons with lived experience (co-development of a video used in the package). Face-to-face introductive session followed by e-learning session, and a face-to-face conclusive session | Providing a service user perspective on perinatal health concerns (risk factors, signs and support needs) | Methods not recorded  Open-ended questions (satisfaction, learning content and service user involvement) | Positive effect of service user involvement (improved attitudes and increased sensitivity to perinatal mental health problems) | (1)  1: N  2: N  3: N  4: N  5: N  Low |
| Pearson et al., 2019  USA | N=16 on the pre-test (62.6% certified nurse midwives) and N=14 on the post-test (50% certified nurse midwives)  Convenience sample | Quantitative non controlled non randomized study  Longitudinal  Pre and post evaluation at 3 month follow up (no control group)  Single-site | 30-minute PowerPoint presentation including discussion (assessment, identification and management of perinatal depression) | Knowledge and confidence levels in assessing and managing PND | Pre- and post-training 6-items self-designed questionnaire (Likert scales) : knowledge about available resources and confidence levels in using screening tools and prescribing treatment or referring to other services | Improved confidence and higher number of referrals / prescriptions | (4)  1: N  2: N  3: N  4: Y  5: N  Low |
| Shinohara et al., 2022  Japan | N=115 participants (midwives (n=105), nurses (n=5) and public health nurses (n=5) randomized to the intervention and control groups (n=58 and n=57 respectively)  Convenience sample (sampling by sending an information letter to midwives associations, birth centers and hospitals) | Quantitative study (parallel-arm randomized controlled  trial)  Longitudinal  Single-site  All participants received the same training but with evaluation at different timings  Intervention = post-evaluation after training on interviewing skills  Control=  post-evaluation before training on interviewing skills | 1-day e-learning training (morning on knowledge on perinatal mental health problems and afternoon on interviewing skills) | Empathy towards parents with perinatal mental health problems  Knowledge of perinatal mental health assessment  Attitudes towards providing psychological support | Empathic communication skills:  - vignette and 5 Likert questions  - video displaying a simulated interview by a midwife and qualitative question (analysis by the two researchers to determine whether the answer is empathic or not, using the Empathic Understanding in Interpersonal Processes scale)  Self-designed 30 items  knowledge of perinatal mental health assessment questionnaire  Counselor Response Form for attitudes towards providing psychological support | Significant improvement in empathic communication skills in the intervention group  Improvement in knowledge in perinatal mental health assessment in both groups (no group differences)  No effect on attitudes towards providing psychological support  . | (2)  1: Y  2: N  3: Y  4: N  5: Y  Moderate |
| Toler et al., 2018  USA | N=54 midwives (96% certified nurse-midwives and 4% certified professional midwives) across 11 sites  Midwives had an average experience of 10 years  Convenience sample | Mixed methods study (quantitative descriptive and qualitative)  Cross-sectional Multi-site | -2-min video about risk factors, symptoms of postpartum anxiety and use of standardized screening tool (EDPS-3A)  -Toolkit for counseling, treatment and referral for screen positive parents | Implementation of a screening program | Description of screening rate for postpartum anxiety  Midwives' feedbacks about issues related to implementation of the screening program (no description of the analysis methods) | 89.6% of eligible parents were screened across the 9 sites (12.5% of parents had a positive EPDS-3A score)  Increased awareness of postpartum anxiety in midwives who found useful to have a toolkit | (1)  1: N  2: N  3: N  4: N  5: N  (4)  1: N  2: N  3: Y  4: Y  5: Y  (5)  1: N  2: N  3: N  4: N  5: Y  Low |
| Varotariya et al., 2019  India | N=31 registered nurses working in obstetrics and gynecology setting (64.51% were general nurses midwives)  Convenience sample | Quantitative non controlled non randomized study  Longitudinal (pre and post test)  Single-site | Training program on postpartum depression (not detailed) | Knowledge on postpartum depression and attitudes (nurse role and barriers to screening) | Pre and post test self-designed questionnaire:    -30 items related to knowledge about PPD (epidemiology and etiology; clinical features; and management and prognosis)  -10 items related to attitudes towards perceived barriers for postpartum screening and nurse role | Significant improvement in knowledge about etiology, epidemiology, clinical features and diagnosis  No changes in knowledge about management and prognosis domains.  Qualitative improvement in perceived barriers to screening and nurse role in screening | (4)  1: N  2: N  3: N  4: Y  5: N  Low |
| Wickberg et al., 2005  Sweden | N=32 midwives  (16 experimental group; 16 control group)  Convenience sample | Quantitative study  Controlled non randomized  Longitudinal  Multi-site | One-afternoon information session about perinatal depression (symptoms, etiology, effects, management) and about the value of listening and support | Influence of midwives' training / awareness of EPDS scores on the evolution of perinatal depressive symptoms | EPDS in gestational week 25 and in gestational week 36. Unlike midwives in the control group, those in the study group were informed when a parent had a score 12 or more at gestational week 25. | Significant within-group reduction in EPDS mean score from week 25 to week 36 in the study group  No group differences in depressive symptoms, healthcare utilization and referral to mental health providers | (3)  1: N  2: N  3: Y  4: Y  5: Y  High |
| Yamashita et al., 2007  Japan | Midwives (n=12; one with experience in inpatient psychiatric services)  No complementary training in psychiatric diagnosis.  Convenience sample | Quantitative descriptive study  Cross-sectional  Single-site | Introduction about perinatal mental health problems and DSM IV. 2 half-day lectures on DSM IV structured interview | Diagnostic accuracy using DSM IV criteria | 29 case-vignettes: diagnosis accuracy (concordance between diagnoses made by midwives and those made by specialized psychiatrist) | Good diagnostic accuracy (kappa between 0.6 and 0.79) for panic disorder, major depression, manic episode and specific phobia. | (4)  1: N  2: N  3: Y  4: N  5: Y  Moderate |

**EPDS:** Edinburgh Postnatal Depression Scale; **FOC:** fear of childbirth; **MMAT**: Mixed Methods Appraisal Tool; **OSCE**: Objective Structured Clinical Examination; **PMHC**: Perinatal Mental Health Care; **PMHPs**: Perinatal Mental Health problems; **PND:** perinatal depression, **PPD:** postpartum depression, **SUD**: substance use disorders
