# Supplementary material for: A systematic review of midwives’ training needs in perinatal mental health and related interventions
Source: Front Psychiatry. 2024 Apr 22;15:1345738. doi: 10.3389/fpsyt.2024.1345738 (PMC11071341; doi:10.3389/fpsyt.2024.1345738)
Supplement: Supplementary Table 6 — List of abbreviations. [file Table_6.docx]

**Supplementary table 6. List of abbreviations**

MMAT: Mixed Methods Appraisal Tool

PMH: peripartum mental health

PMHC: peripartum mental health care

PMHPs: Peripartum mental health problems

RCT: randomized controlled trial

SMI: serious mental illness
